# Metabolomics and Transcriptomics Analyses of Two Contrasting Cherry Rootstocks in Response to Drought Stress

**DOI:** 10.3390/biology10030201

**Published:** 2021-03-06

**Authors:** Tian Wan, Ying Feng, Chenglin Liang, Liuyi Pan, Ling He, Yuliang Cai

**Affiliations:** 1 College of Horticulture, Northwest Agriculture & Forestry University, No.3 Taicheng Road, Yangling 712100, China; wtian2012@nwsuaf.edu.cn (T.W.); xtdyuer@163.com (Y.F.); panliuyi314@126.com (L.P.); 2 Haidu College, Qingdao Agricultural University, Laiyang 265200, China; ljl2015lcl@163.com

**Keywords:** *Prunus mahaleb* CDR-1, *P.cerasus* × *P. canescens* Gisela 5, water deficiency, central metabolites, biosynthesis pathways, transcriptome

## Abstract

**Simple Summary:**

Drought is one of the main factors affecting sweet cherry yields, and cherry rootstocks can provide a range of tree vigor levels to better match sweet cherries with the characteristics of the soil. To investigate the molecular responses of the cherry rootstocks to water deficiency, we performed transcriptomic and metabolomic analyses of two contrasting cherry rootstocks—Mahaleb CDR-1 and Gisela 5. The results revealed that differentially expressed metabolites related to the pathways of cyanoamino acid metabolism and phenylpropanoid biosynthesis may be key factors in the difference in drought resistance in the two rootstocks. Moreover, six central metabolites—3-cyanoalanine, phenylalanine, quinic acid, asparagine, p-benzoquinone, and phytosphingosine—were identified as potential biological markers of the drought response in cherries and may be key factors in the difference in drought resistance, along with caffeic acid and chlorogenic acid. Furthermore, we selected 17 differentially expressed genes as core candidate genes and the mechanism of a drought-tolerant cherry rootstock (DT) in response to drought is summarized. This study can provide a valuable insight into the molecular mechanisms behind drought resistance and will be beneficial to those aiming to breed promising new cherry cultivars.

**Abstract:**

Drought is one of the main factors affecting sweet cherry yields, and cherry rootstocks can provide a range of tree vigor levels to better match sweet cherries with the characteristics of the soil. To investigate the molecular events of the cherry to water deficiency, we performed transcriptomic and metabolomic analyses of *Prunus mahaleb* CDR-1 (drought-tolerant cherry rootstock (DT)) and *P. cerasus* × *P. canescens* Gisela 5 (drought-susceptible cherry rootstock (DS)), respectively. The results revealed 253 common drought-responsive genes in leaves and roots in DT and 17 in DS; 59 upregulated metabolites were explored in leaves in DT and 19 were explored in DS. Differentially expressed metabolites related to the cyanoamino acid metabolism pathway and phenylpropanoid biosynthesis pathway may be key factors in the difference in drought resistance in the two rootstocks. Moreover, six central metabolites—3-cyanoalanine, phenylalanine, quinic acid, asparagine, p-benzoquinone, and phytosphingosine—were identified as potential biological markers of drought response in cherries and may be key factors in the difference in drought resistance, along with caffeic acid and chlorogenic acid. We also selected 17 differentially expressed genes as core candidate genes and the mechanism of DT in response to drought is summarized.

## 1. Introduction

Drought is among the most serious challenges to crop production in the world today. As a major abiotic stress, drought can severely affect the yield and quality of agricultural production systems [1,2,3]. The drought resistance of the sweet cherry (*Prunus avium*) rootstock remains an important tool for extending the site adaptability of cultivars in China, especially in the Loess Plateau region of northwest China [4,5]. Although knowledge of the physiological responses of *Prunus* species to drought is increasing [6,7,8], little is known about the link between drought tolerance and the associated foundations of metabolomes and transcriptomes. It is of great importance for breeders to understand the molecular responses of cherry or *Prunus* fruit trees to drought stress and to develop novel molecular approaches to enhance their tolerance to drought.

Different species respond differently to drought stress. Most cherry rootstocks are susceptible to water deficits, and drought tends to interfere with plant growth, reproduction, and the absorption and transport of nutrients [9]. Germplasms with contrasting drought tolerance are ideal materials, and the drought resistance of *Prunus* rootstocks is closely related to their genetic background [10]. In cherry production, the Mahaleb (*P. mahaleb*) and Gisela (hybrids of *P. cerasus* and *P. canescens*) series are both great potential rootstocks, with different genetic backgrounds and contrasting responses to drought [11]. The former is a drought-tolerant species that can survive in extremely dry conditions and a native species to Europe and Western-Asia in thickets on dry karst areas, which shows great potential for cherry rootstock breeding [11,12,13]; Mahaleb CDR-1 (*P. mahaleb*) is an important rootstock variety and is most widely used in northern China [4]. The latter is largely known as a drought-susceptible species; owing to its good properties, such as early fruit-bearing and dwarfing, it is an important rootstock for breeding cherry seedlings and is popular with farmers in the world, especially Gisela 5 (*P. cerasus* × *P. canescens*) and Gisela 6 (*P. cerasus* × *P. canescens*) [4,11,14].

From the point of view of system biology, performing omics joint analysis can be a good way of understanding the mechanism behind drought regulation in plants; such analyses have been widely used in modern biology to characterize the molecular responses of plants to abiotic stress [15]. Metabolomics can unravel these complex mechanisms by measuring the metabolites that participate in various biochemical processes. In response to abiotic stress, plants can regulate their metabolic networks and synthesize a series of metabolites that can help them repair the damage [16]. It is of great significance to reveal the mechanisms behind the plants’ responses to stress. High-throughput transcriptome analysis, which focuses on transcripts with functional information in the plant genome, is widely used in the field of plant stress research [17] on plants such as Mongolian almond [18], *Ginkgo biloba* L. [19], and wheat [20]. Combining transcriptomics and metabolomics is an effective means of exploring the responses of plants to stress. You et al. [21] found that amino acid metabolism and abscisic acid metabolism and signaling play important roles in drought tolerance in sesame, and Pan et al. [22] underscored the significance of 23 core metabolic processes in annual ryegrass. In addition, multiomics has been used to study other abiotic stress responses related to temperature [23,24], nutrition [25], salinity [26], light [27,28], and so on.

Our objective is to present a comprehensive overview of the metabolomes and transcriptomes of contrasting cherry rootstocks and to infer the core regulatory networks to reveal the relationships among metabolites and transcript pathways. This study can provide a valuable insight into the molecular mechanisms behind drought resistance and will be beneficial to those aiming to breed promising new cherry cultivars.

## 2. Materials and Methods

### 2.1. Plant Material, Stress Treatment, and Sampling

The experiments were conducted at Northwest A&F University, Yangling, China (34°20′ N, 108°24′ E). *P. mahaleb* CDR-1 (drought-tolerant cherry rootstock (DT)) and *P. cerasus × P. canescens* Gisela 5 (drought-susceptible cherry rootstock (DS)) seedlings were obtained through cutting propagation, and the obtained cuttings were used as test materials with the same growth status and robust growth without pests and diseases. Drought resistance was assessed in a preliminary experiment that was consistent with the reported conclusions on the two rootstocks [11,12]. Biennial cutting seedlings were grown in a glasshouse with a day/night temperature of 28 °C/18 °C. Each genotype contained one stress group (SG) and one control group (CG): SG was used for drought stress treatment, and CG was used as a well-watered control. All genotypes were irrigated every 3 days to field capacity as needed, fertilized weekly with Hoagland’s solution, and irrigated in the same manner before treatment. In this period, the soil moisture in the pots was maintained at about 75% of the field capacity. Then irrigation was stopped in SG, but watering continued in CG as before. Drought stress was monitored according to the leaf relative water content. Material was sampled at the first appearance of leaf wilting 10 days after drought stress. The leaf relative water content of drought-stressed plants was 65% in DT (vs. 89% in control) and 67% in DS (vs. 87% in control). Leaves and root tip tissues (about 1 to 2 cm, including root cap, meristematic zone and elongation zone) were collected in three biological replicates for the transcript analysis, and leaf samples were harvested in six biological replicates for the metabolite analysis (Table 1). The harvested samples were immediately frozen in liquid nitrogen and stored at −80 °C until needed for further analyses.

### 2.2. RNA Sequencing and Functional Annotation

Total RNA was isolated with TRIzol reagent (Tiangen Biotech, Beijing, China) according to the manufacturer’s protocol. The RNA quality was controlled using NanoPhotometer spectrophotometer (IMPLEN, Westlake Village, CA, USA), Qubit 2.0 Flurometer (Life Technologies, CA, USA) and Agilent Bioanalyzer 2100 system (Agilent Technologies, CA, USA). A total amount of 1.5 µg RNA per sample was used as input material for the RNA sample preparations. Sequencing libraries were generated using NEBNext Ultra RNA Library Prep Kit for Illumina (NEB, Ipswich, MA, USA) following the manufacturer’s recommendations and index codes were added to attribute sequences to each sample. Briefly, mRNA was purified from total RNA using poly-T oligo-attached magnetic beads. Fragmentation was carried out using divalent cations under elevated temperature in NEBNext First Strand Synthesis Reaction Buffer (5X). First strand cDNA was synthesized using random hexamer primer and M-MuLV Reverse Transcriptase (RNase H^−^). Second strand cDNA synthesis was subsequently performed using DNA Polymerase I and RNase H. Remaining overhangs were converted into blunt ends via exonuclease/polymerase activities. After adenylation of 3′ ends of DNA fragments, NEBNext Adaptor with hairpin loop structure were ligated to prepare for hybridization. In order to select cDNA fragments of preferentially 150~200 bp in length, the library fragments were purified with AMPure XP system (Beckman Coulter, Beverly, LA, USA). Then 3 µl USER Enzyme (NEB, Boston, MA, USA) was used with size-selected, adaptor-ligated cDNA at 37 °C for 15 min followed by 5 min at 95 °C before PCR. Then PCR was performed with Phusion High-Fidelity DNA polymerase, Universal PCR primers and Index (X) Primer. At last, PCR products were purified (AMPure XP system, Brea, CA, USA) and library quality was assessed on the Agilent Bioanalyzer 2100 system.

cDNA libraries were built, and the qualified libraries were subjected to paired-end sequencing using the Illumina 4000 sequencer by Novogene (Beijing, China). Raw data reads were filtered to remove adapter sequences, reads containing more than 10% unknown bases, and low-quality sequences to generate clean data. The clean data were mapped to the reference genome [29] with HISAT. The expression of each gene was calculated and normalized by the corresponding fragments per kilobase of transcript per million mapped fragments (FPKM) with cufflinks. We selected differentially expressed genes (DEGs) by performing the negative binomial test in the DESeq package [30]. DEGs were annotated through comparison with previously annotated genes in public databases, the National Center for Biotechnology Information nonredundant database, the Swiss-Prot database, Gene Ontology (GO), and euKaryotic 87tssOrthologous Groups/Clusters of Orthologous Groups. Pathway analysis was performed with the Kyoto Encyclopedia of Genes and Genomes (KEGG) database.

### 2.3. Quantitative Real-Time PCR (qRT-PCR)

The qRT-PCR was performed with a Life Technologies QuantStudio 5 using SYBR Premix Ex Taq Kit (Takara Biotechnology Co., Ltd., Dalian, China). Primers used for the qRT-PCR are listed in Table S1. The RT-PCR was performed under the following conditions: 95 °C for 10 min, followed by 35 cycles of 95 °C for 3 s, and 60 °C for 30 s. Each sample was analyzed in triplicate. *ACTIN* was used as a reference gene to normalize the relative expression of selected genes. The relative expression (fold changes) of 10 candidate genes was calculated with the 2^−ΔΔCt^ method and translated to log2 fold changes to compare with the RNA-seq results.

### 2.4. Metabolomics Data Processing and Analysis

The mixed samples were subjected to gas chromatography–mass spectrometry (GC-MS). Chroma TOF 4.3X by LECO Corporation and the LECO-Fiehn Rtx5 database were used to exact raw peaks, filter the data baselines, calibrate the baselines and peaks, perform deconvolution analysis, and integrate the peak areas. The retention time index method was used in the peak identification, and the retention time index tolerance was 5000. Principal component analysis (PCA) and orthogonal projections to latent structures—discriminant analysis (OPLS-DA) were performed with SIMCA (V14.1; MKS Data Analytics Solutions, Umea, Sweden). Central metabolites were selected based on the variable importance in the projection (VIP) and *p* value. Metabolites with VIP > 1.0 and *p* < 0.05 were selected as central metabolites. Related pathways analysis was performed with KEGG (http//www.genome.jp/kegg/, accessed on 10 April 2018), NIST (http//www.nist.gov/index.html, accessed on 10 April 2018), and MetaboAnalyst (http//www.metaboanalyst.ca, accessed on 10 April 2018). Quantitative values of central metabolites were used to calculate the Euclidean distance matrix (Euclidean short matrix). The full-chain approach was used to cluster the central metabolites, and the results were displayed as a heat map. An interaction analysis of central metabolites was performed using MetaScape (Cytoscape3.6.1).

### 2.5. DEGs and Conjoint Analysis of Central Metabolites

Data on DEGs and differentially expressed metabolites were used for conjoint analysis and mapping to the KEGG database to identify the pathways in which genes and metabolites participated. The analysis also explored the common and different metabolic processes upstream and downstream involving genes and metabolites of the two genotypes. The results were used to infer the mechanisms behind their differential drought resistance.

### 2.6. Screening of Core Candidate Genes and Bioinformatics Analysis

Candidate genes for the drought response (mainly focusing on upregulated genes) were screened based on three factors: fold change, abundance of gene expression, and DEGs between the two genotypes. The DEGs were filtered by ta logarithm two-fold change |log2FC| ≥ 1 and padj < 0.05 as screening standards. GO terms and KEGG pathways fulfilling the criterion of a Bonferroni-corrected *p*-value ≤ 0.05 were defined as significantly enriched in DEGs. BLAST E-value ≤ 10^−5^ and HMMER E-value ≤ 10^−10^ were set as select parameters. DEGs protein−protein interaction analysis was performed by STRING (confidence limits ≥ 700) and visualized on Cytoscape3.6.1 software. GO classification, KEGG classification, interaction analysis of DEGs, and conjoint analysis of central metabolites and DEGs were used to further screen the core set of candidate genes. The bioinformation of the core candidate genes was analyzed, including the coding sequence, the amino acid sequence, protein properties, and subcellular localization prediction. Using the National Center for Biotechnology Information ORF finder, we obtained the coding sequence of each candidate transcription sequence. Protein subcellular localization prediction was done online (https://wolfpsort.hgc.jp/, accessed on 10 April 2018), and the physical and chemical properties of predicted proteins were identified online as well (https://web.expasy.org/protparam/, accessed on 10 April 2018).

## 3. Results

### 3.1. Transcriptional Characteristics of the Responses of Cherry Rootstock to Drought Stress

**Figure 1:**
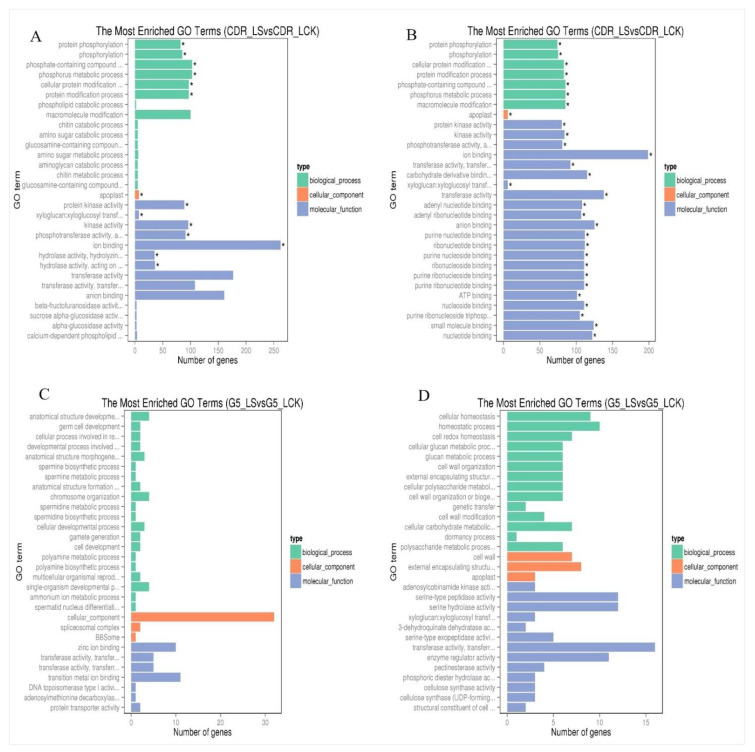
Functional annotation of drought-responsive genes upregulated and downregulated in CDR-1 and Gisela 5 leaves based on Gene Ontology (GO) categorization. (**A**) DEGs upregulated in CDR-1 leaves; (**B**) DEGs downregulated in CDR-1 leaves; (**C**) DEGs upregulated in Gisela 5 leaves; (**D**) DEGs downregulated in Gisela 5 leaves. Asterisks represent significantly enriched genes.

**Figure 2:**
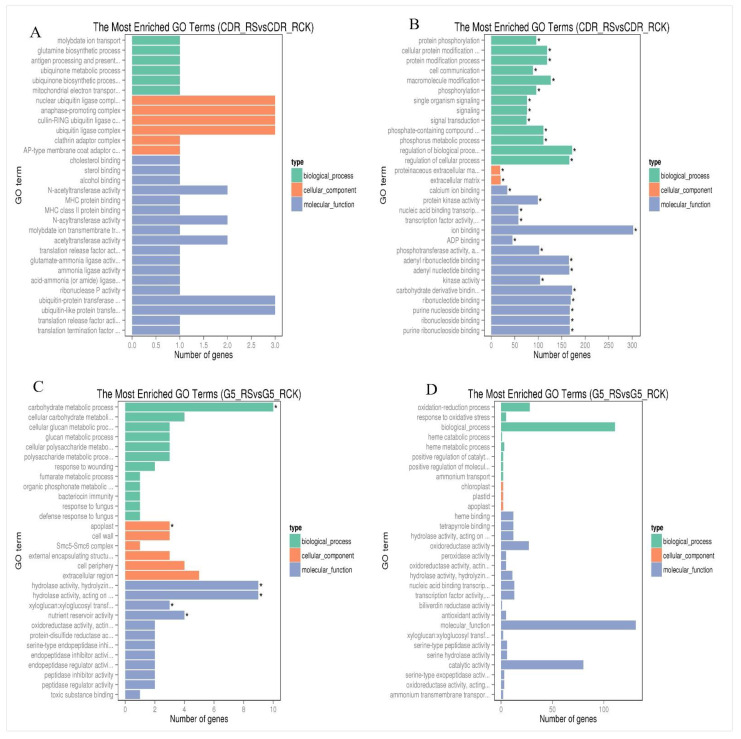
Functional annotation of drought-responsive genes up- and downregulated in CDR-1 and Gisela 5 root tips based on Gene Ontology (GO) categorization. (**A**) DEGs upregulated in CDR-1 roots; (**B**) DEGs downregulated in CDR-1 roots; (**C**) DEGs upregulated in Gisela 5 roots; (**D**) DEGs downregulated in Gisela 5 roots. Asterisks represent significantly enriched genes.

The resulting set of 24 samples yielded more than 1.11 billion clean reads, and more than 75.3% of map rates were mapped to the cherry genome (Table S2). A total of 2070 DEGs were identified in DT, and 746 DEGs were identified in DS. Of these DEGs, 73 and 49 were explored in both rootstocks, in leaves and root tissues, respectively (Figure S1). The 365 upregulated DEGs (281 in leaves and 84 in roots) and 1705 downregulated DEGs (626 in leaves and 1079 in roots) were detected in CDR-1, while 150 DEGs (79 in leaves and 71 in roots) were upregulated and 605 (400 in leaves and 205 in roots) were downregulated in Gisela 5, respectively. Moreover, 253 and 17 DEGs were detected between leaves and root tissues, respectively, in CDR-1 and Gisela 5. Many transcription factors (TFs) and genes related to enzymes were downregulated in both genotypes. Only some TFs were upregulated, including IFH, ninja-family protein AFP3, CCR4-associated homolog, and COL domain in leaf tissues and extensin-3-like, U-box domain-containing protein 21-like, and chitotriosidase-1-like in roots (Table S3). Most DEGs were enriched in about 150 and 440 GO terms in leaf and root tissues in DT, respectively, and about 13 and 70 GO terms in DS, respectively. The top 30 GO enrichment terms are displayed in Figure 1 and Figure 2.

In addition, highly expressed and repressed (|log2FC| > 3) unique transcripts were assembled (Table S3), specifically in DT (Table S4). The abundance of expression of most DEGs was much higher in roots than in leaves. Four common transcripts upregulated between leaves and roots were selected: glutaredoxins, serine/threonine-protein kinase, a seed maturation protein, and one hypothetical protein. Nine commonly repressed transcripts were detected, including ERFs, brassinosteroid-regulated proteins, LRR receptors, putative receptor proteins, cytochrome P450 94C1, and MYB-related proteins. The extent of the decrease of downregulated DEGs was much more in roots than in leaves.

In DT, specific transcripts highly expressed in leaves included low-temperature-induced protein, galactinol–sucrose galactosyltransferase, stachyose synthase-like, and so on. Specific repressed transcripts included dehydration-responsive protein RD22, alpha-trehalose-phosphate synthase, receptor-like protein kinase, and so on (Table S4). Roots mainly involved TFs, translocator protein homologs, septum-promoting GTP-binding protein, late embryogenesis abundant protein, and sodium-potassium-calcium exchanger; repressed transcripts were related to TFs, protein phosphatase 2C, serine/threonine-protein kinase, disease resistance protein, and so on (Table S5). In DS, specific induced transcripts in leaves included protein phosphatase 2C, zinc finger proteins, 2-aminoethanethiol dioxygenase, and others (Table S6). In DS roots, the transcripts included TF NAC4, U-box domain-containing protein, protein phosphatase 2C, and others (Table S7).

### 3.2. Interaction Networks of DEGs between DT and DS

**Figure 3:**
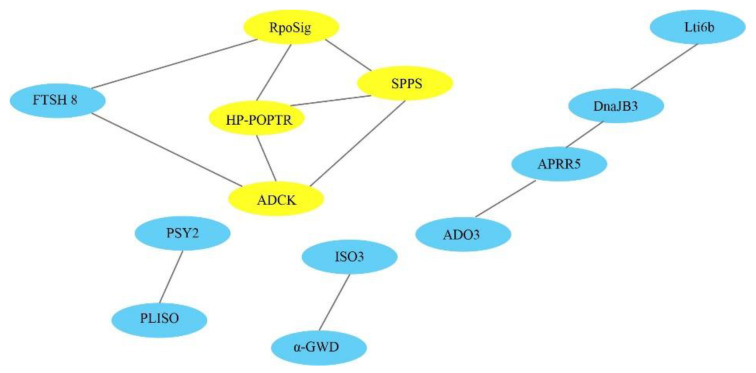
Interaction networks of upregulated DEGs in the drought-tolerant species CDR-1. Yellow ellipses show that genes interacted with at least three other genes. Blue ellipses show that genes interacted with fewer than three other genes. RpoSig, RNA polymerase sigma factor (AT5G24120.1); SPPS, solanesyl-diphosphate synthase (AT1G17050); HP-POPTR, hypothetical protein POPTR_0019s08420g (AT3G56290.1-P); ADCK, aarF domain-containing protein kinase (AT3G24190.1); FTSH 8, ATP-dependent zinc metalloprotease FTSH 8 (AT1G06430.1); Lti6b, PM-YC3.6-Lti6b (AT3G05890.1); DnaJB3, DnaJ homolog subfamily B member 3 (AT1G56300.1); APRR5, two-component response regulator-like APRR5 (AT5G24470.1); ADO3, adagio protein 3 (AT1G68050.1); PSY2, phytoene synthase 2, chloroplastic-like (AT5G17230.3); PLISO, prolycopene isomerase, chloroplastic (AT1G57770.1); ISO3, isoamylase 3, chloroplastic (AT4G09020.1); α-GWD, alpha-glucan water dikinase, chloroplastic (AT1G10760).

DEG interaction networks were built to show the relationships between these genes in biological systems and to understand how these response genes interact with one another under drought stress. From upregulated DEGs in DT leaves, we obtained two interaction networks related to terpenoid biosynthesis and the regulation of plant circadian rhythm. In the first, solanesyl-diphosphate synthase interacted with RNA polymerase sigma factor, aarF domain-containing protein kinase, and ATP-dependent zinc metalloprotease FTSH 8 as part of terpenoid biosynthesis. In the second, PM-YC3.6-Lti6b interacted with DnaJ homolog subfamily B member 3, two-component response regulator-like APRR5, two-component response regulator-like APRR9, and adagio protein 3 as part of the regulation of the plant circadian rhythm in leaves (Figure 3).

Moreover, downregulated transcripts in the DT leaves interacted intensively around plant hormone signal transduction, plant–pathogen interaction, starch and sucrose metabolism, linoleic acid, stilbenoid, diarylheptanoid, and gingerol metabolism. Downregulated transcripts in the DT roots interacted around vesicular transport, the biosynthesis of secondary metabolites, starch and sucrose metabolism, cysteine and methionine metabolism, RNA degradation, glycerophospholipid metabolism, linoleic acid metabolism, and amino sugar and nucleotide sugar metabolism. However, interaction among upregulated DEGs was not detected in our tests in either leaves or roots of the DS samples. However, ribosome biogenesis and starch, sucrose, amino sugar, and nucleotide sugar metabolism were obtained among downregulated DEGs in leaves, and cell energy metabolism was found among downregulated DEGs in roots.

**Figure 4:**
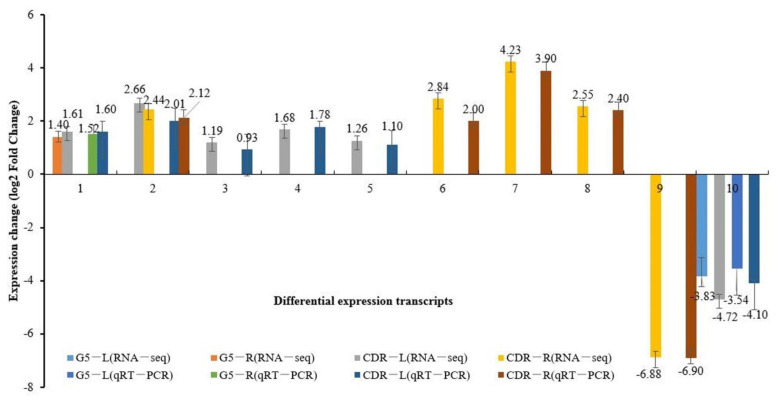
Changes in gene expression levels were confirmed using qRT-PCR. G5-L, leaves of Gisela 5; G5-R, root tissues of Gisela 5; CDR-L, leaves of Mahaleb CDR-1; CDR-R, root tissues of Mahaleb CDR-1. Transcripts ID: **1**, Pav_sc0001339.1_g200.1.mk; **2**, Pav_sc0002493.1_g100.1.mk; **3**, Pav_sc0000131.1_g130.1.mk, **4**, Pav_sc0000004.1_g040.1.mk; **5**, Pav_sc0000311.1_g710.1.mk; **6**, Pav_sc0000638.1_g820.1.mk; **7**, Pav_sc0001335.1_g050.1.mk; **8**, Pav_sc0001479.1_g020.1.mk; **9**, Pav_sc0009842.1_g030.1.mk; **10**, Pav_sc0000893.1_g020.1.mk.

To verify the reliability of the RNA-seq results, we randomly selected 10 DEGs, detected their expression by qRT-PCR, and compared the results to the RNA-seq data. Two upregulated DEGs (PAV_SC00001339.1_g2001.mk and PAV_SC0002493.1_g1001.mk) and eight downregulated DEGs (pav_SC0000893.1_g020.mk and others) were selected for qRT-PCR detection. The comparison showed that the RNA-seq expression was consistent with the qRT-PCR expression (Figure 4).

### 3.3. Metabolome Analysis and Screening of Central Metabolites

From 24 test samples, namely, six quality controls in each group (Table 1), a total of 517 peaks were detected, and after data preprocessing, 461 effective peaks were evident (Figure S2). To compare differences in metabolic profiles among SG and CG, we first used PCA to visualize the impact of drought on the DT and DS metabolomes. A PCA model was created with four groups: QC (quality control samples), TOTAL (total samples), CCL-CSL (CG of DT leaves vs. SG of DT leaves), and GCL-GSL (CG of DS leaves vs. SG of DS leaves). The model showed the distribution of the origin data, and all sample data lay inside the 95% confidence region (Hotelling’s T^2^ ellipse). The results indicated an obvious separation between the two genotypes and between SG and CG of CDR-1 (Figure S3A); no distinct boundary was observed between SG and CG of Gisela 5 (Figure S3C).

To obtain a higher level separation and a better understanding of the variables responsible for the classification, we then performed supervised OPLS-DA, which revealed the contributions of variables to the difference between the two groups and improved the classification. Finally, more satisfactory modeling and prediction results were obtained. Both SG and CG of the two genotypes were clearly separated from the control along PC1 (Figure S3B,D). The R^2^Y and Q^2^ values were 0.997 and 0.767 in DT and 0.961 and 0.509 in DS, respectively (Table S8), which indicated that the metabolites of SG had changed significantly compared to CG in both genotypes.

### 3.4. Identification and Cluster Analyses of Cherry Metabolites in Response to Drought

Differentially expressed metabolites were selected based on OPLS-DA model VIP values (>1) and significant changes between SG and CG (Student’s *T* test, *p* < 0.05). A total of 234 differentially expressed metabolites were obtained in both genotypes, and 92 metabolites were upregulated. There were 14 common central metabolites in the two genotypes, including proline, asparagine, and quinic acid (Table 2), whose accumulations were generally higher than those of other metabolites. For example, 3-cyanoalanine increased by 4,111,229- and 2.1-fold in DT and DS, respectively. Moreover, 59 specific upregulated metabolites were detected in DT, including p-benzoquinone (p-BQ), melibiose, flavin adenine degrad product, salicin, serine, and citrulline (Table S9), which were mainly carbohydrate conjugated compounds, organic acids, and amino acids. A total of 19 specific upregulated metabolites were detected in DS, including maleic acid alanine, D-glyceric acid, and malonic acid (Table S10).

### 3.5. Analysis of Metabolic Pathways and DEGs

Differentially expressed metabolites and DEGs were mapped together into the KEGG database to mine candidate genes, associated with the enrichment of central metabolites, in the same metabolic pathways. Eight amino acids, 16 organic acids, and other secondary metabolites and related genes were enriched in 16 metabolic pathways, including cyanoamino acid metabolism and phenylpropanoid biosynthesis (Table S11).

Cyanoamino acid metabolism was the most notable pathway that enriched 3-cyanoalanine, phenylalanine, asparagine, glycine, serine, alanine, valine, and isoleucine (Figure S4). 3-cyanoalanine, phenylalanine, and asparagine were the common central metabolites in both genotypes, but with great differences in the amounts of their increases. Serine and glycine were specifically upregulated in DT and interacted directly in this pathway. In contrast, alanine, valine, and isoleucine were specifically upregulated in DS (Figure S4). The genes involved in cyanoamino acid metabolism in DT were three β-glucosidase genes, one amygdalin hydrolase precursor gene, one amino acid transferase gene, and one mannitol lyase gene. Those in DS were one β-glucosidase gene and several genes encoding unknown functional proteins (Table S11).

The phenylpropanoid biosynthesis pathway was related to quinic acid, citric acid, 4-hydroxy-3-methoxycinnamaldehyde, and chlorogenic acid. Quinic acid was upstream and phenylalanine was downstream. Chlorogenic acid hydrolysis produced quinic acid and caffeic acid (reaction ID: RO299). Chlorogenic acid (reaction ID: RO194) was produced by quinic acid and caffeoyl-coenzyme A (CoA), whereas caffeoyl-CoA was produced by caffeic acid, ATP, and CoA. Quinic acid, chlorogenic acid, and caffeic acid were upregulated and specifically accumulated in DS (Figure S5). In DT, quinic acid, phenylalanine, and p-coumaric acid increased significantly, and from upstream to downstream there was quinic acid, phenylalanine, and p-coumaric acid. In the phenylpropanoid biosynthesis pathway, the upregulated DEGs were mainly coumarin-CoA ligase, β-glucoside enzyme, and laetrile hydrolase precursor genes in DT and the β-glucoside enzyme gene and peroxidase genes in DS (Table S11).

### 3.6. Selection of Core Candidate Genes

Key pathways and candidate genes were acquired by GO analysis, KEGG analysis, and interaction analysis of protein–protein interaction networks of DEGs. After conjoint analysis of central metabolites, we focused on upregulated genes involved in differential metabolites or metabolic pathways (Table S11), such β-glucosidase, 4-coumarate-CoA ligase and so on. A total of 17 DEGs were selected as core candidate genes with high abundance, significant differences, and participation in several important metabolic pathways. Among them, four genes were involved in energy metabolism: β-amylase, β-glucosidase, ATP-binding cassette transporter G family member 22 and sugar transporter ERD6-like 16. The β-glucosidase gene was involved in many metabolic pathways, such as cyanoalanine metabolism and phenylpropane synthesis, and encoded a protein with 508 amino acids (Table 3). In addition, three ubiquitin ligase genes (E3 ubiquitin-protein ligase, protein gene with U-box, and protein gene with F-box) were selected with specific upregulation in DS. One TF involved in the drought response was screened, namely, zinc finger protein gene. It was upregulated and differentially expressed in leaves and root tips in DT and downregulated in leaves in DS; it was not expressed in roots in DS. The zinc finger gene was predicted to encode a protein with 111 amino acids, and subcellular localization was predicted in the nucleus, extracellular membrane, and chloroplasts (Table 3).

## 4. Discussion

We used metabolomic and transcriptomic analyses to analyze metabolites and related genes involved in the drought response of two contrasting cherry rootstocks, *P. mahaleb* CDR-1 and *P. cerasus* × *P. canescens* Gisela 5 [11,12]. When cherry rootstocks were exposed to severe drought, a large number of genes were downregulated, and relatively fewer genes were upregulated (Figure S3). This may be due to a decrease in metabolic activity following the stress. Phytohormones regulate plant response to drought stress by integrating external stimuli with complex regulatory networks. In this study, a large number of hormone-related genes were significantly upregulated or downregulated in the roots and leaves of the two cherry rootstocks after exposure to drought. The plant hormone pathways involved in regulation included the abscisic acid, ethylene, cytokinin, salicylic acid, brassinosteroid, and jasmonic acid signaling pathways, which were mainly focused on DT in our previous studies [31]. In contrast, upregulated genes and upregulated metabolites are likely closely related to improvement in plant resistance and survival and correspond to the increased activity of soluble sugar, proline, and peroxidase in the physiological response [32,33,34,35], which was also a focus of this study.

Under water deficit, the plants’ metabolic balance is readjusted and metabolic responses to drought stress in plants have attracted more attention. Ferulic acid was reported to provide protection to photosynthesis during drought stress [36], and 4-hydroxycinnamic acid and ferulic acid were considered as key metabolites for rice drought-tolerance [37]. We identified six central metabolites—3-cyanoalanine, phenylalanine, quinic acid, asparagine, p-benzoquinone, and phytosphingosine as potential biological markers of the drought response in cherries. Moreover, metabolites involved in plant stress response—the accumulation of sugar (such as fructan) or amino acids (such as proline)—serve as osmoprotectant under drought stress [21]. In this study, a large number of organic acids were correlated with drought resistance and we divided them into three groups.

The first group included most amino acids: asparagine, arginine, cysteine, glycine, serine, alanine, valine, isoleucine, and so on. These amino acids play key roles in regulating cell osmosis, reducing active oxygen damage, and keeping enzymes and proteins stable [38,39,40]. Asparagine, a nitrogen-transportable amino acid in plants that is associated with resistance to disease and adversity, accumulated in large amounts in both genotypes. Asparagine synthase genes improve stress resistance [41,42]. However, serine and glycine accumulated specifically in DT, whereas alanine, valine, and isoleucine accumulated specifically in DS (Figure S4). In both genotypes, different amino acids accumulated under drought stress, as mentioned above.

The second group, proline and linolenic acid have been reported widely in plant abiotic stress responses as regulation compounds. The proline biosynthesis gene *P5CS* was upregulated in both genotypes. Linolenic acid accumulated in DT as the main ingredient in galactolipid. Linolenic acid belongs to the cell membrane lipids and is involved in responses to multiple stresses, such as drought, cold, and high temperature [43,44,45,46]. It increased in the drought-tolerant cultivar, in accordance with a study on grape seedlings under drought stress [47]. However, linolenic acid remained relatively unchanged in the drought-susceptible cultivar. It may work as a regulation lipid on the cell membrane to reduce the damage caused by drought stress.

The third group included cyanoalanine, phenylalanine, quinic acid, coumaric acid, citric acid, caffeic acid, chlorogenic acid, and others. Cyanoalanine, phenylalanine, and quinic acid were common central response metabolites in DT and DS. Cyanoalanine synthase can convert toxic cyanide into nontoxic cyanide alanine and further into asparagine in plants [48,49]. Cyanoalanine derivatives are generated by HCN conjugates, being a coproduct of ethylene synthesis [50]. Cyanoalanine increased by million folds in DT under water deficit but was almost undetectable in the well-watered treatment. In contrast, it increased 1.5 times in DS. This result is in keeping with research on *Caragana korshinskii*, in which cyanoalanine increased 6.9 times under drought stress [51]. Cyanoalanine may be the product of cyanide detoxification. At the same time, we found that asparagine, its downstream product, increased 7.0 and 2.6 times in DT and DS, respectively. This further confirms that cyanide turns into a harmless substance after detoxification and synthesizes into metabolites that are beneficial to plant growth. The detoxification of cyanide was more efficient in DT than in DS; in other words, the former suffered less under the same drought conditions. The efficiency of detoxification to avoid cell damage under drought stress is an important factor in determining drought resistance. However, cyanoalanine synthase genes could not be screened in this work, as they may have been triggered and expressed at an earlier period.

The synthesis of phenylalanine was more efficient in DT than in DS under drought stress. However, reports of the role of phenylalanine in the drought response are mixed. Consistent with this study, phenylalanine increased in potatoes [52]; however, it decreased in chickpeas [53]. This phenomenon may be related to plant species, duration of stress, or stress level. As an upstream compound of the phenylalanine synthesis pathway, quinic acid is also a precursor to lignin synthesis [54] and increased million folds in DT but only 1.5 times in DS. This result is in accordance with reports on Oleaceae trees [55] but is opposite to findings on peaches, in which it decreased [56]. Quinic acid is involved in the resistance of plant cells to oxidation [57] and can improve plant resistance to strong light and high temperature [58,59]. These findings suggest that phenylalanine and quinic acid are closely related to plant response to drought or stress, but the response mechanisms need to be verified further. In addition, p-coumaric acid increased significantly in DT. Downstream of phenylalanine and quinic acid, it might also be related to cherry response to drought. However, caffeic acid and chlorogenic acid were specifically induced in DS (Figure S5). Plants produce cyanide under stress, and the accumulation of cyanide can be toxic to plant cells, although plants themselves have detoxification systems to reduce this damage.

**Figure 5:**
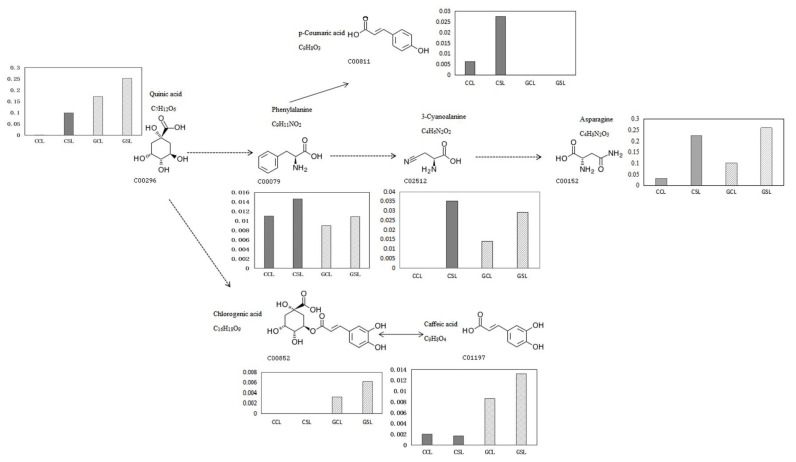
The pathway diagram of key drought-responsive metabolites from two cherry rootstocks. Solid arrows indicate direct reactions between metabolites. Dashed arrows indicate indirect reactions between metabolites. CCL and CSL represent control and drought treatment samples of CDR-1. GCL and GSL represent control and drought treatment samples of Gisela 5. Because the relative content of substances was detected by nontarget GC-MS, there are no units.

According to these findings, the common pathway between DT and DS was quinic acid → phenylalanine → cyanoalanine → asparagine, but the efficiency of the synthesis of quinic acid, cyanoalanine, and asparagine differed (Table 2). First, DT was significantly higher than Gisela 5, especially cyanoalanine. Second, there may have been a difference in the response pathways. In DT the pathway was quinic acid → phenylalanine → p-coumaric acid, whereas in DS it was more likely to be quinic acid → coffeic acid → chlorogenic acid (Figure 5). The main factors contributing to the difference were p-coumaric acid, coffeic acid, chlorogenic acid, and other specific metabolites, such as serine and glycine in DT and alanine, valine, and isoleucine in DS.

Furthermore, in DT, the most induced metabolites also included 2,3-dimethyl succinic acid, maleamate, and phytosphingosine. Many derivatives of succinic acid have been proposed as regulators of plant growth and herbicides, and 2,3-dimethyl succinic acid is one. They act as signaling compounds to integrate energetic metabolism and the hormonal systems of plants [60]. Succinic acid preparations can increase plant resistance to unfavorable conditions (drought, cold, etc.), which is helpful in protecting plants from frost, decreasing infection, and enhancing the chlorophyll content of leaves [61]. There are limited reports of maleamate in plant metabolism. Ethyl N (3,4-dichlorophenyl) maleamate acts as an inhibitor or suppressor of crabgrass [62], although little is known about the role of maleamate in plants.

Moreover, genes related to lipid synthesis and transport were upregulated in DT leaves, whereas genes involved in lipid metabolism were upregulated in roots and leaves. Sphingolipids (SPLs), a diverse group of lipids, are present in all eukaryotes. The main SPL in plants is phytosphingosine, which can inhibit cell growth and nutrient transport [63] and respond to plant diseases [64]. SPLs are dynamic regulators of plant cellular processes and are essential for basic cellular functions, cell tissue, and motility [65]. Phytosphingosine increased significantly under drought treatment in DT, and thus could be used as a marker metabolite. Moreover, functional analysis of SPL biosynthesis demonstrated that these lipids were directly involved in many aspects of plant development and response to environmental changes, including biotic and abiotic stimuli. The modification of lipid composition differed in drought-tolerant and drought-susceptible plants under stress, which suggests that lipid composition is of great significance to the drought resistance of plants. However, the specific role of plant secondary metabolites in enhancing drought resistance is not fully understood and needs to be explored further [66]. According to DT transcriptome analysis, glycogen biosynthesis and glycosyl hydrolase genes were downregulated to rebuild energy homeostasis, NCED homology genes were induced for stomatal regulation and water conservation, expression of linolenic acid and amino acid synthesis genes was generally increased to enhance drought tolerance, and TFs (CBF/NF-Ys, MYB, WRKY and U-box) may regulate key functional genes to adapt to stress, as reported in our previous research [31].

**Figure 6:**
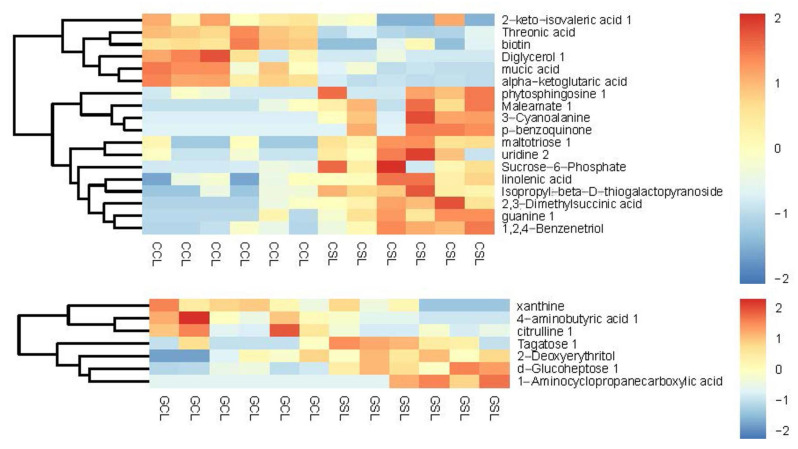
Heat map of the hierarchical clustering analysis of CDR-1 (CCL, CSL) and Gisela 5 (GCL, GSL). CCL, CSL, GSL, and GCL are the same as in Figure 4. Increasing and decreasing contents of metabolites are shown in orange and blue, respectively.

Finally, when we combined metabolomic analysis with transcript analysis, we detected proline, aspartate, and glutamate metabolism; purine metabolism; and galactose metabolism as common biological responses to coping with drought in both cherry rootstocks. According to the interaction analysis of differentially expressed metabolites, the main metabolites (Figure 6) were mapped into seven pathways: tricarboxylic acid (TCA) cycle; arginine metabolism; proline, glutamate, and asparagine metabolism; and other metabolism (Figure S6). However, in DS, D-glucoheptose tagatose, 2-deoxyerythritol, and 1-aminocyclopropanecarboxylic acid increased significantly. Differentially expressed metabolites also included tagatose, 4-aminobutyric acid, citrulline, and xanthine in DS (Figure 6), which were involved in mutual interaction pathways (Figure S7). In most studies, organic acids and TCA cycle intermediates increase in response to drought stress or temperature but decrease in glycophytes after salt stress [67]. Among carbonyl compounds, p-BQ and maltotriose, except 3-cyanoalanine, increased significantly in DT. As the electron acceptor in PS II activity, p-BQ increases dramatically after drought, whereas main proteins and elements involved in photosynthesis are repressed under water deficiency [68]. p-BQ may play a crucial role in maintaining photosynthetic capacity; however, there is limited information on this response. As carbohydrate conjugates, maltotriose with glucose, sucrose, galactose, fructan, and trehalose were reported to be involved in plant abiotic stress responses [67,69,70]. Sugars not only represent an energy source but also are precursors to carbon, substrates for polymers, storage and transport compounds, and signaling molecules.

## 5. Conclusions

**Figure 7:**
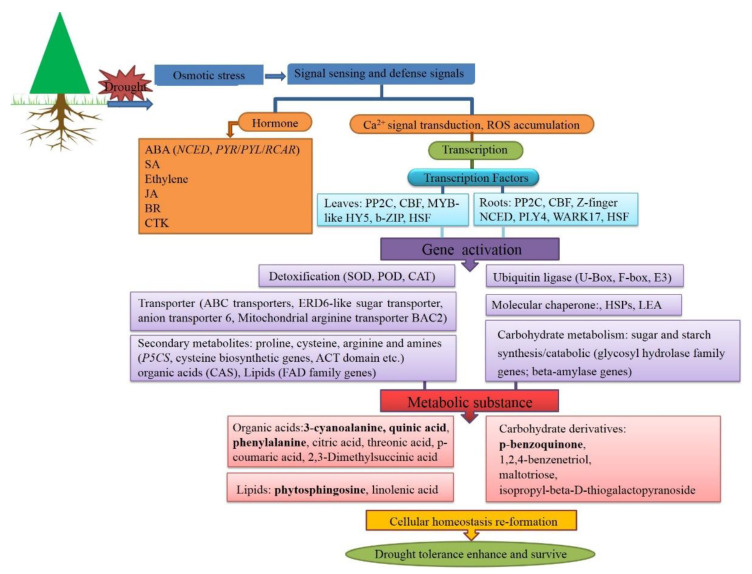
Deductive diagram of the drought-tolerant cherry rootstock *P. mahaleb* CDR-1.

We comprehensively analyzed overall changes in metabolic profiles in two contrasting cherry rootstocks and also performed transcript analysis. The results focused on the TCA cycle, energy metabolism, and lipid metabolism pathways, which were strongly relevant in DT and DS. Central metabolites and DEGs related to the cyanoalanine and phenylpropane metabolism pathways were the key factors in the difference in drought resistance of DT and DS. The drought-tolerant cherry appears to adapt to water deficits by expressing constitutively high levels of some protective metabolites, such as quinic acid and asparagine, and some specific metabolites, such as serine and glycine, in DT. Moreover, we explored 17 core candidate genes (Table 3) and screened six, including candidate metabolite-quinic acid, phytosphingosine, 3-cyanoalanine, p-BQ, and phenylalanine. Combining transcriptional and physiological results for DT [31], we created a deductive diagram of drought regulation mechanisms to describe and understand the drought regulation process of the drought-tolerant rootstock *P. mahaleb* CDR-1 (Figure 7). The metabolic pathways identified, functional validation of drought-responsive genes, and drought regulation mechanisms deduced in this study help uncover the complexity of drought tolerance at the molecular level and will be useful for breeding drought-tolerant cherry cultivars.

## Figures and Tables

**Table 1:** Experimental sampling code.

Material	Tissue	Group	Samples for RNA-seq	Samples for GC-MS Tests
DT	Leaves	Drought stress	CDR-LS1, CDR-LS2, CDR-LS3	CDR-LS1, CDR-LS2, CDR-LS3 CDR-LS4, CDR-LS5, CDR-LS6
		Control	CDR-LCK1, CDR-LCK2, CDR-LCK3	CDR-LCK1, CDR-LCK2, CDR-LCK3 CDR-LCK4, CDR-LCK5, CDR-LCK6
	Root tip tissues	Drought stress	CDR-RS1, CDR-RS2, CDR-RS3	-
		Control	CDR-RCK1, CDR-RCK2, CDR-RCK3	-
DS	Leaves	Drought stress	G5-LS1, G5-LS2, G5-LS3	G5-LS1, G5-LS2, G5-LS3 G5-LS4, G5-LS5, G5-LS6
		Control	G5-LCK1, G5-LCK2, G5-LCK3	G5-LCK1, G5-LCK2, G5-LCK3 G5-LCK4, G5-LCK5, G5-LCK6
	Root tip tissues	Drought stress	G5-RS1, G5-RS2, G5-RS3	-
		Control	G5-RCK1, G5-RCK2, G5-RCK3	-

DT, *P. mahaleb* CDR-1 (drought-tolerant cherry rootstock). DS, *P. cerasus* × *P. canescens* Gisela 5 (drought-susceptible cherry rootstock). CDR-LS, the leaf samples of DT in stress group; CDR-LCK, the leaf samples of DT in control group; G5-LS, the leaf samples of DS in stress group; G5-LCK, the leaf samples of DS in control group; CDR-RS, the root samples of DT in stress group; CDR-RCK, the root samples of DT in control group; G5-RS, the root samples of DS in stress group; G5-RCK, the root samples of DS in control group. 1, 2, 3, 4, 5 and 6 (the number at the end of each abbreviation) represent each biological replicate, respectively.

**Table 2:** Common central metabolites in cherry rootstocks.

Metabolite	Mean CCL	Mean CSL	FC	VIP	*p*	Mean GCL	Mean GSL	FC	VIP	*p*
Proline	0.8516	0.9086	1.1	1.034	0.048	0.2199	0.4656	2.1	1.096	0.042
Asparagine	0.0321	0.2258	7.0	1.141	0.049	0.1021	0.2609	2.6	1.273	0.022
Quinic acid	0.0005	0.0985	182.6	2.381	0.031	0.1715	0.2519	1.5	1.282	0.048
Purine riboside	0.0196	0.0785	4.0	1.228	0.039	0.1414	0.1594	1.1	1.333	0.028
Glutamine	0.0349	0.1036	3.0	1.416	0.014	0.0111	0.0917	8.3	1.085	0.010
3-cyanoalanine	8.5523 × 10^−9^	0.0352	4,111,229.0	2.902	0.012	0.0141	0.0292	2.1	1.032	0.028
L-allothreonine	0.0130	0.0245	1.9	1.358	0.014	0.0114	0.0206	1.8	1.233	0.026
Phenylalanine	0.0110	0.0146	1.3	1.165	0.040	0.0090	0.0109	1.2	1.080	0.049
Fucose	0.0045	0.0075	1.7	1.519	0.027	0.0049	0.0079	1.6	1.567	0.046
Salicylic acid	0.0037	0.0059	1.6	1.000	0.027	0.0040	0.0074	1.8	1.264	0.008
Sulfuric acid	0.0035	0.0056	1.6	1.000	0.032	0.0028	0.0060	2.2	1.081	0.006
Phytosphingosine	0.0016	0.0118	7.3	1.295	0.044	0.0216	0.0359	1.7	1.085	0.032
Biotin	0.0013	0.0002	0.2	1.032	0.047	0.0001	0.0002	2.0	1.021	0.048
D-glucoheptose	0.0016	0.0033	2.1	1.565	0.031	0.0010	0.0042	4.4	1.256	0.000

Common central metabolites here refer specifically to upregulated metabolites. GSL and GCL represent the drought stress treatment group and control group of Gisela 5 leaves; CSL and CCL represent the drought stress treatment group and control group of CDR-1 leaves. FC (fold change) indicates the ratio of the peak amount of drought stress groups (GSL, CSL) and the peak amount of control groups (GCL, CCL). Differences are significant at *p* < 0.05. Because the relative content of substances was detected by nontarget GC-MS, there are no units.

**Table 3:** Biological information on core candidate genes in the responses of cherry rootstocks to drought.

Gene ID	Putative Function	Number of Amino Acids	Theoretical pI	Putative Subcellular Localization
Pav_sc0006061.1_g110.1.mk	Beta-amylase 3, chloroplastic [*Prunus avium*]	547	9.01	Chloroplasts, nucleus, mitochondria
Pav_sc0005750.1_g010.1.br	Beta-glucosidase 11-like	508	6.25	Vacuole, chloroplasts, extracellular membrane
Pav_sc0000004.1_g040.1.mk	ABC transporter G family member 22-like isoform X3 [*Prunus avium*]	603	8.23	Plasma membrane, vacuole
Pav_sc0004467.1_g120.1.mk	Sugar transporter ERD6-like 16	376	6.84	Plasma membrane, nucleus
Pav_sc0000491.1_g270.1.mk	Stachyose synthase-like, raffinose synthase or seed imbibition protein	867	5.66	Chloroplasts, cytoplasm, and nucleus
Pav_sc0001335.1_g550.1.mk	RNA polymerase sigma factor sigE, chloroplastic/mitochondrial [*Prunus avium*]	550	10.00	Nucleus, chloroplasts, nucleus, mitochondria
Pav_sc0002893.1_g310.1.mk	Alpha-glucan water dikinase, chloroplastic isoform X1 [Glycine max]	1468	6.40	Chloroplasts
Pav_sc0001335.1_g050.1.mk	Probable protein phosphatase 2C 51	395	7.62	Chloroplasts, nucleus, and cytoplasm
Pav_sc0001405.1_g970.1.mk	Temperature-induced lipocalin-1 [*Prunus avium*]	185	5.97	Nucleus and cytoplasm
Pav_sc0002493.1_g100.1.mk	Early light-induced protein 1, chloroplastic-like [*Prunus avium*]	94	10.16	Chloroplasts, nucleus
Pav_sc0000138.1_g610.1.mk	Two-component response regulator-like APRR5	691	5.96	Nucleus
Pav_sc0001305.1_g820.1.mk	Late embryogenesis abundant protein 1-like	164	9.40	Mitochondria, chloroplasts, cytoplasm
Pav_sc0000719.1_g800.1.mk	E3 ubiquitin-protein ligase At4g11680 isoform X1	330	8.94	Plasma membrane, vacuole, endoplasmic reticulum, and Golgi apparatus
Pav_sc0000464.1_g820.1.mk	U-box domain-containing protein 4	383	6.03	Nucleus, cytoplasm
Pav_sc0000067.1_g380.1.mk	F-box/LRR-repeat MAX2 homolog A-like	654	5.86	Nucleus, cytoplasm
Pav_sc0000586.1_g780.1.mk	Homeobox-leucine zipper protein ATHB-12-like	322	4.58	Nucleus
Pav_sc0004290.1_g270.1.mk	Zinc finger CCCH domain-containing protein 23-like [*Prunus avium*]	111	4.60	Nucleus, chloroplasts, extracellular membrane

Note: The most likely located organelle is ranked first; possibility is ranked from high to low.

## Data Availability

The sequencing raw data for 24 samples can be accessed in the NCBI Sequence Read Archive (SRA) database under the accession number of SRP095080 (*Prunus mahaleb* CDR-1) and PRJNA704726 (*P. cerasus* × *P. canescens* Gisela 5).

## Supplementary Materials

The following are available online at https://www.mdpi.com/2079-7737/10/3/201/s1, Table S1: Primers used for the quantitative real-time PCR. Table S2: Transcriptome Illumina sequencing and mapped results for CDR-1 and Gisela 5. Table S3: Common transcripts involved in the drought response in CDR-1 and Gisela 5 leaves and roots. Table S4: Highly induced and repressed transcripts unique to CDR-1. Table S5: Highly induced and repressed transcripts unique to CDR-1. Table S6: Highly induced and repressed transcripts unique to Gisela 5 leaves. Table S7: Highly induced and repressed transcripts unique to Gisela 5 roots. Table S8: Explanation and predictability of the principal component analysis (PCA) and partial least squares–discriminant analysis (PLS-DA). Table S9 Differential drought responded upregulated metabolites in CDR-1. Table S10 Differential drought responded upregulated metabolites in Gisela 5. Table S11 Candidate DEGs involved in KEGG pathways that are related to differential metabolites. Figure S1: Venn diagrams of genes significantly differentially expressed between drought and well-watered treatments in CDR-1 and Gisela 5 roots and leaves. Figure S2: GC-TOFMS ion chromatogram of all samples. Figure S3: PCA model and OPLS-DA model score scatter plot for CDR-1 and Gisela 5 drought-responsive differential metabolites. Figure S4: Drought-responsive metabolites and genes in cherry rootstocks involved in the cyanoamino acid metabolism pathway. Figure S5: Drought-responsive metabolites and genes in cherry rootstocks involved in the phenylpropanoid biosynthesis pathway. Figure S6: Interaction analysis of differentially expressed metabolites in CDR-1. Figure S7: Interaction analysis of differentially expressed metabolites in Gisela 5.

## Abbreviations

DT	Drought-tolerant cherry rootstock
DS	Drought-susceptible cherry rootstock
TCA cycle	Tricarboxylic acid cycle
DEGs	Differentially expressed genes
PPI	Protein−Protein Interaction
ABA	Abscisic acid
SG	Stress group
CG	Control group
LWC	Relative water content
FPKM	Fragments Per Kilobase of transcript per Million mapped fragments
GO	Gene Ontology
KOG/COG	euKaryotic 87tssOrthologous Groups/Clusters of Orthologous Groups
KEGG	Kyoto Encyclopedia of Genes and Genomes
qRT-PCR	Quantitative real-time PCR
GC-MS	Gas chromatography-mass spectrometer
RI	Retention time index
QC	Quality control
PCA	Principal component analysis
OPLS-DA	Orthogonal projections to latent structures-discriminant analysis
VIP	Variable importance in the projection
CDS	Coding sequence
TFs	Transcription factors
