# Supplementary material for: Metabolomics and Transcriptomics Analyses of Two Contrasting Cherry Rootstocks in Response to Drought Stress
Source: Biology (Basel). 2021 Mar 6;10(3):201. doi: 10.3390/biology10030201 (PMC8001747; doi:10.3390/biology10030201)
Supplement: Supplementary file 1 [file biology-10-00201-s001.zip › biology-1120820-supplementary/Supplementary File/Supplemental Figures.docx]

Supplemental Figures

**Metabolic responses of two contrasting cherry rootstocks to drought stress combining with transcriptomics**


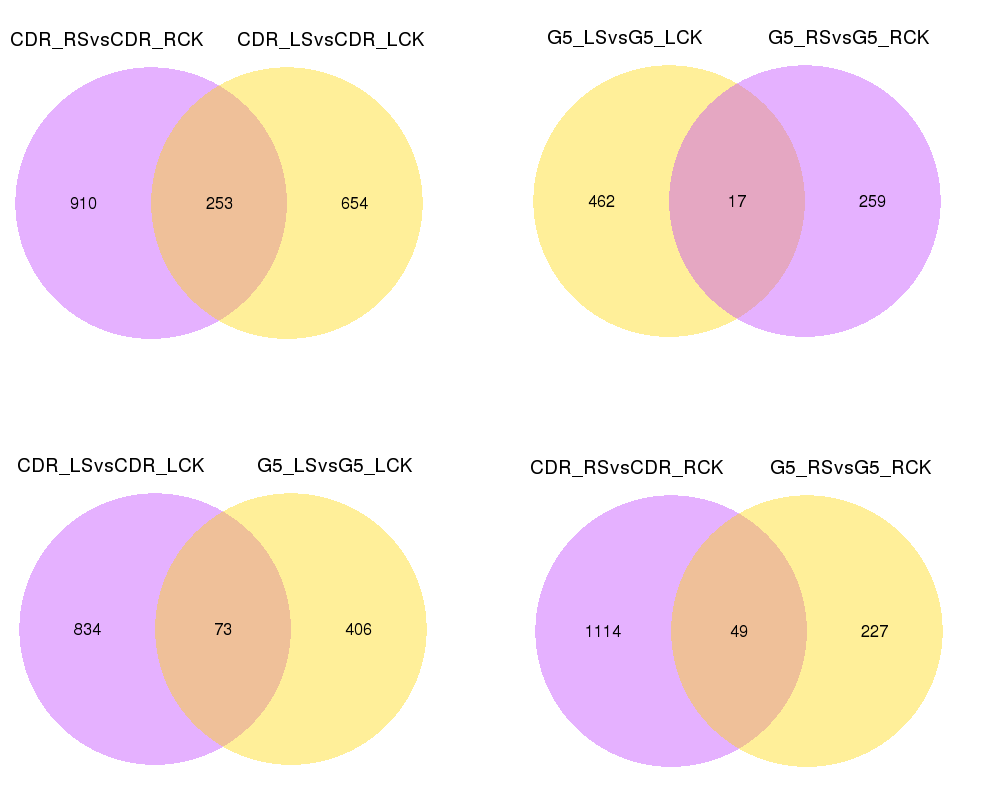


**Figure S1** Venn diagrams of significantly differentially expressed transcripts between drought and well watered treatments in ‘CDR-1’ and ‘Gisela 5’ roots and leaves. CDR-LS vs CDR-LCK indicates DEGs in 'CDR-1' leaves, CDR-RS vs CDR-RCK indicates DEGs in 'CDR-1' roots, G5-LS vs G5-LCK indicates DEGs in 'Gisela 5' leaves, G5-RS vs G5-RCK indicates DEGs in 'Gisela 5' roots.

**Figure S2** Changes in gene expression levels were confirmed using quantitative reverse transcription polymerase chain reaction (qRT-PCR). **G5-L** leaves of ‘Gisela 5’. **G5-T** Root tissues of ‘Gisela 5’. **CDR-L** leaves of Mahaleb ‘CDR-1’. **CDR -T** Root tissues of Mahaleb ‘CDR-1’. Transcripts ID: **1** av_sc0001339.1_g200.1.mk, 2. Pav_sc0002493.1_g100.1.mk, 3. Pav_sc0000131.1_g130.1.mk, 4. Pav_sc0000004.1_g040.1.mk, 5. Pav_sc0000311.1_g710.1.mk, 6. Pav_sc0000638.1_g820.1.mk, 7. Pav_sc0001335.1_g050.1.mk, 8. Pav_sc0001479.1_g020.1.mk, 9. Pav_sc0009842.1_g030.1.mk, 10. Pav_sc0000893.1_g020.1.mk


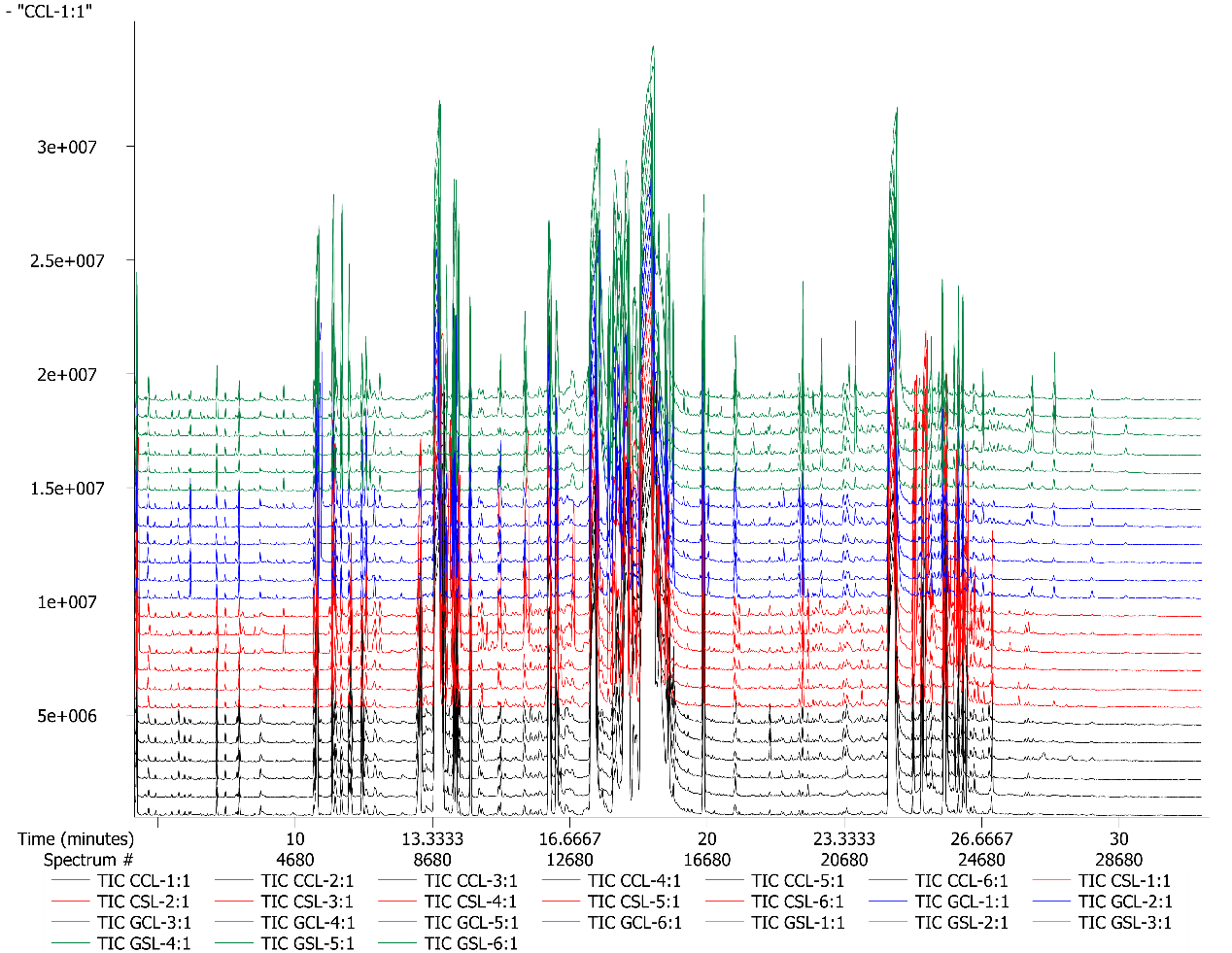


**Figure S3** GC-TOFMS ion chromatogram of all samples. GSL and GCL represent drought-stress treatment group and control group of ‘Gisela 5’ leaves; CSL and CCL represent drought -stress drought treatment group and control group of ‘CDR-1’ leaves.


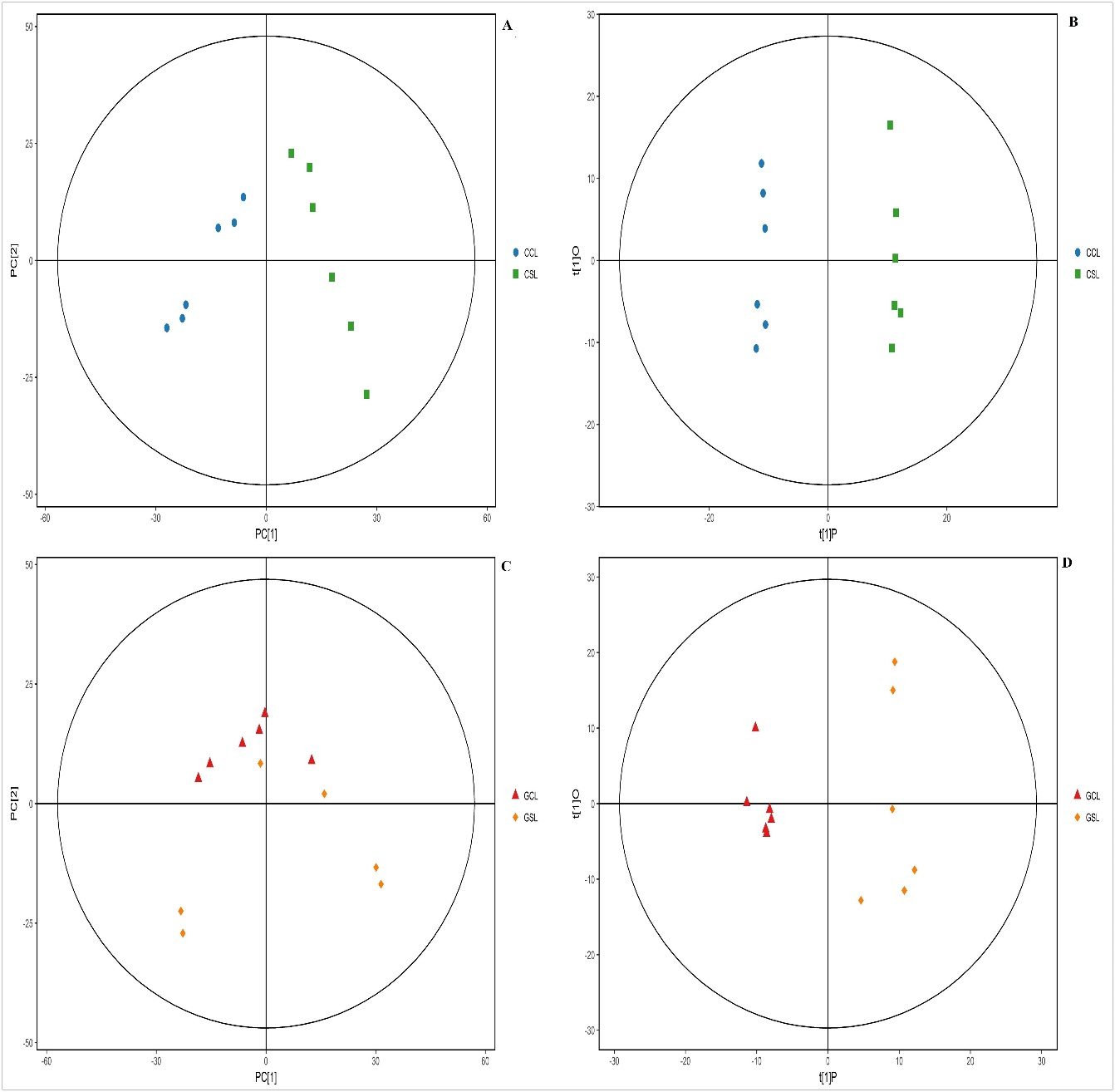
**Figure S4** PCA model and OPLS-DA model score scatter plot of ‘CDR-1’ and ‘Gisela5’ drought responded differential metabolites. **A** PCA model of ‘CDR-1’ group CCL vs. CSL. **B** OPLS-DA model of ‘CDR-1’ group CCL vs. CSL; **C** PCA model of ‘Gisela 5’ group GCL vs. GSL. **D** OPLS-DA model of ‘Gisela 5’ group GCL vs. GSL


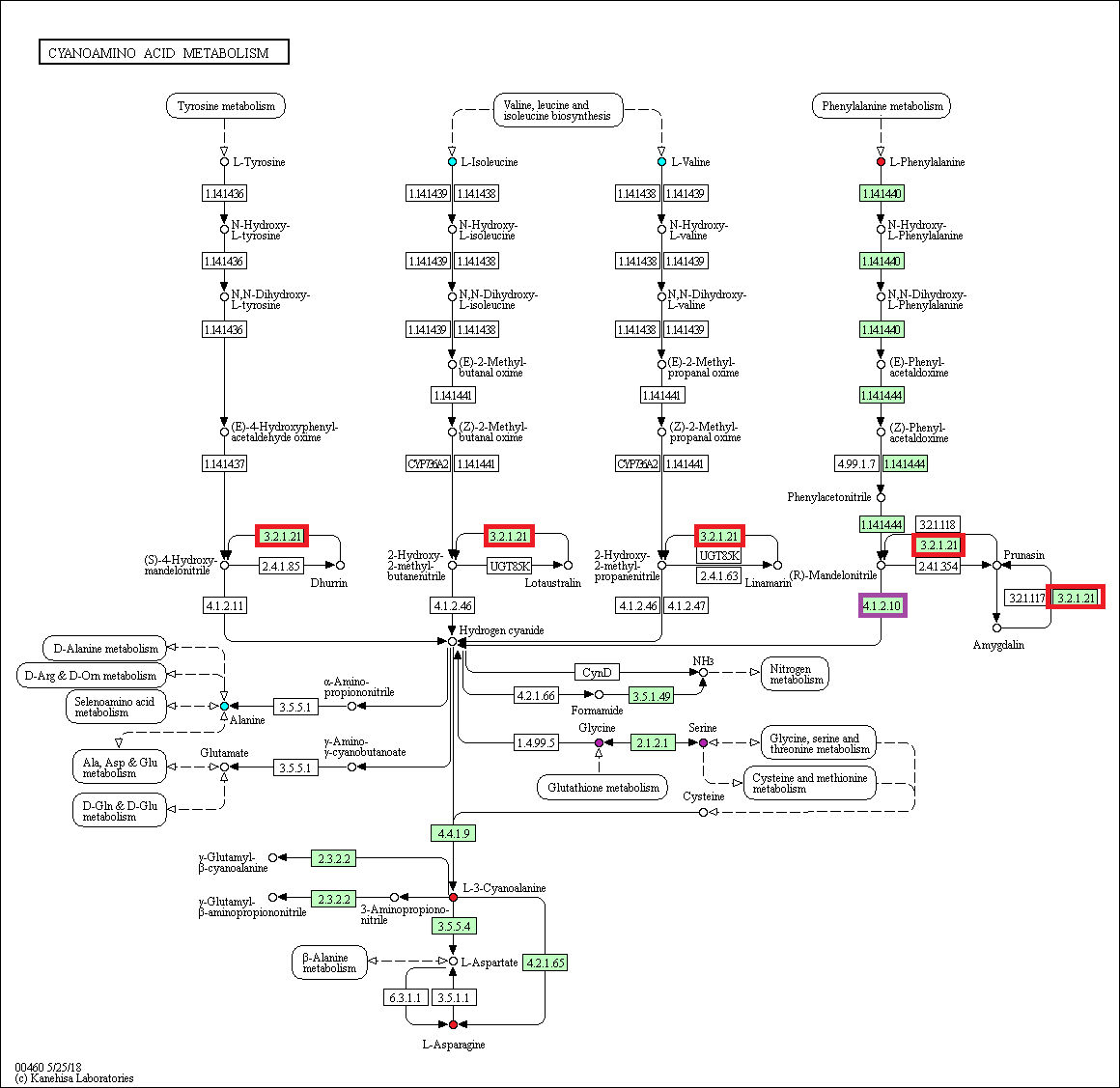


**Figure S5** Drought responded metabolites and genes in cherry rootstocks involved in cyanoamino acid metabolism pathway. **Red box** positive regulated genes in the same reaction of the two rootstocks. **Purple box** positive regulated genes of ‘CDR-1’. **Red dot** the common responded metabolites of two rootstocks. **Purple dot** responded metabolites of ‘CDR-1’. **Blue dot** responded metabolite of ‘Gisela 5’. Genes ID of ‘CDR-1’ participated in the reaction: 3.1.1.21：18770479，18778261，18774021；4.1.2.10：18790921，18790921. Genes ID of ‘Gisela 5’ participated in the reaction: 3.1.1.21：18792862，18775538，18766146，18774013.


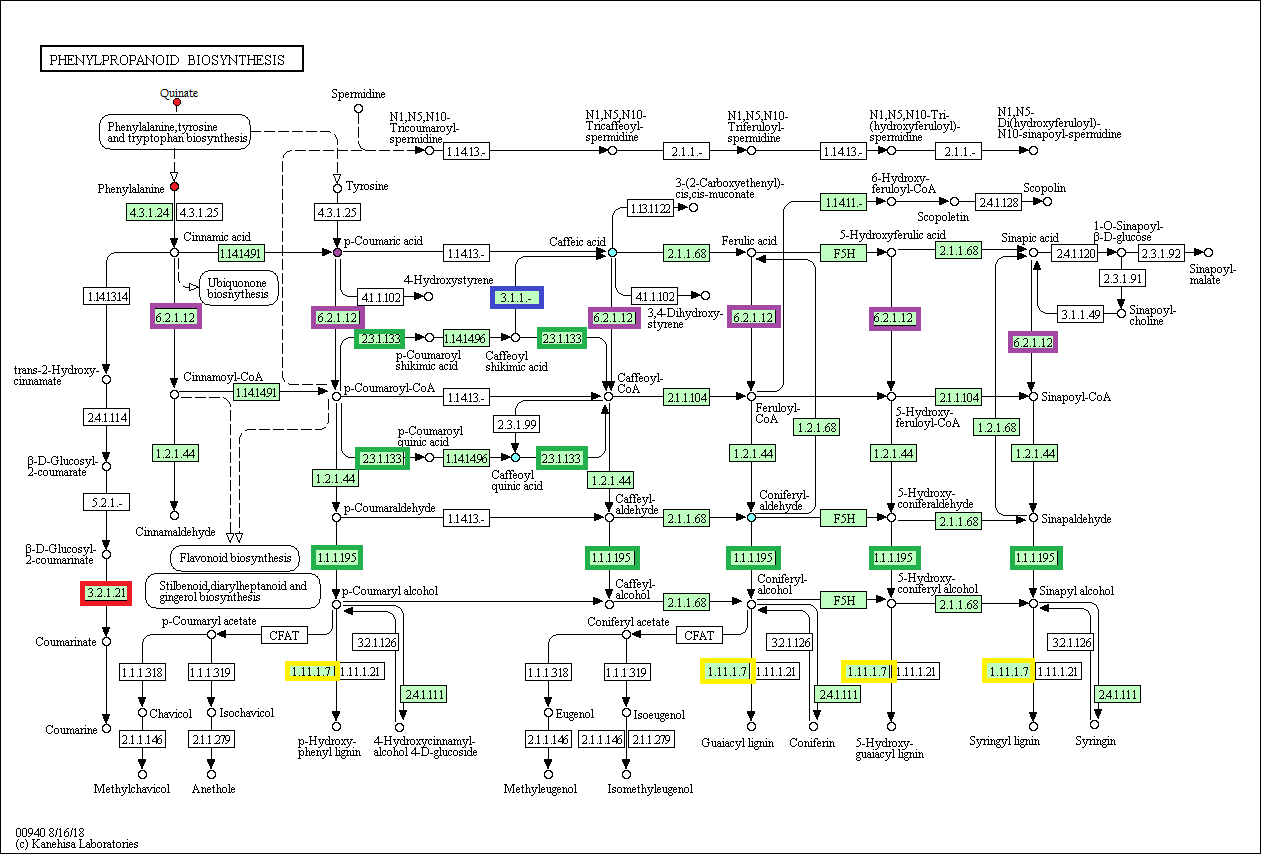


**Figure S6** Drought responded metabolites and genes in cherry rootstocks involved in phenylpropanoid biosynthesis pathway. Red box and green boxes represent positive or negative regulated genes in the same reaction of the two rootstocks respectively. **Purple box** positive regulated genes of ‘CDR-1’. **Blue box** negative regulated genes of ‘CDR-1’. **Yellow box** positive regulated genes in ‘CDR-1’ while negative in ‘Gisela 5’. **Red dot** the common responded metabolites of two rootstocks. **Purple dot** responded metabolites of ‘CDR-1’. **Blue dot** responded metabolites of ‘Gisela 5’. Genes ID of ‘CDR-1’ participated in the reaction: 3.2.1.21:18778261，18774021，18770479；6.1.2.12：18786755；3.1.1-：18777009；23.1.133：18783187；1.1.1.195: 18767368；1.11.1.7：18774869，18773443. Genes ID of ‘Gisela 5’ participated in the reaction: 3.2.1.21:18792862；1.1.1.195: 18774869，18773431；1.11.1.7：18793937，18774869；23.1.133：18782933. Reactions: ①RO1945:Caffeoyl-CoA + Quinate CoA + Chlorogenate；②RO1943:ATP + Caffeate + CoA AMP + Diphosphate + Caffeoyl-CoA；③RO2997:Chlorogenate + H2O Caffeate + Quinate. Caffeoyl quinic acid, also named Chlorogenate or chlorogenic acid; Caffeate also named Caffeic acid, Quinate also named Quinic acid.


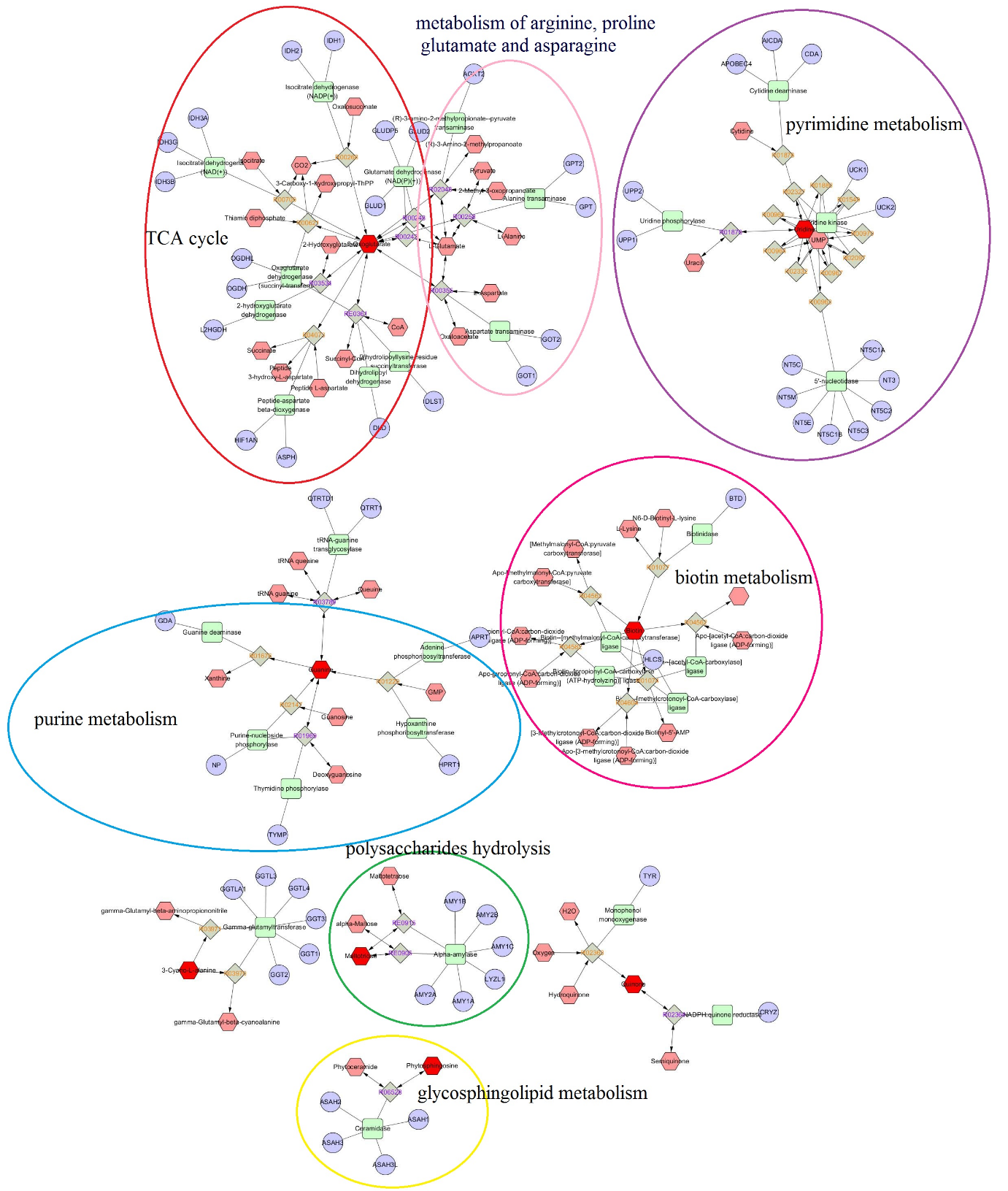


**Figure S7** Interaction analysis of differentially expressed metabolites in ‘CDR-1’


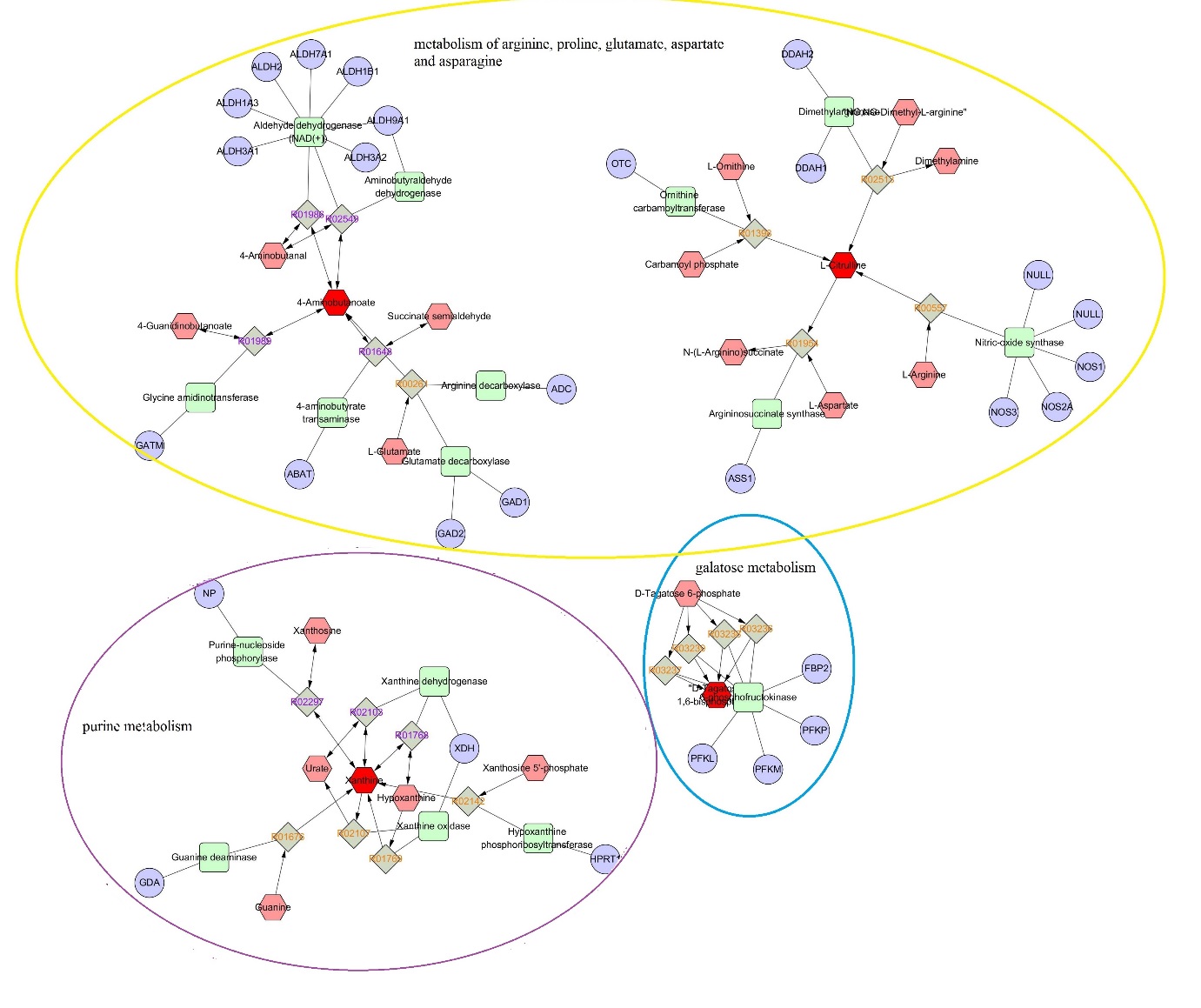


**Figure S8** Interaction analysis of differentially expressed metabolites in ‘Gisela 5’
