# Supplementary material for: Metabolomics and Transcriptomics Analyses of Two Contrasting Cherry Rootstocks in Response to Drought Stress
Source: Biology (Basel). 2021 Mar 6;10(3):201. doi: 10.3390/biology10030201 (PMC8001747; doi:10.3390/biology10030201)
Supplement: Supplementary file 1 [file biology-10-00201-s001.zip › biology-1120820-supplementary/Supplementary File/Supplemental Tables.docx]

Supplementary Tables

**Metabolic responses of two contrasting cherry rootstocks to drought stress combining with transcriptomics**

**Table S1** Primers used for the qRT-PCRs

| **Candidate Genes** | **Forward Primers (5’-3’)** | **Reverse Primers (5’-3’)** | **Amplicon length** |
| --- | --- | --- | --- |
| Pav_sc0000004.1_g040.1.mk | CACCAGAGCAAGCCCAGAAT | TCCCCTTGACGAAGAACTGAA | 183 |
| Pav_sc0000311.1_g710.1.mk | GATAACGAAGGCAAGCGAAGT | TCGGGTTCTGAGGGTGATTC | 132 |
| Pav_sc0002493.1_g100.1.mk | AGGAAGAGCAACCGTCTACAATAC | GCCAGAGCTGAAACAAACCC | 187 |
| Pav_sc0000004.1_g040.1.mk | ATACCATTCGCACAGGAAGTCA | TGTAACCAGTTCGAGTGATGCA | 184 |
| Pav_sc0000131.1_g130.1.mk | GGCTCGTCACTACCGCAATAC | TGTTCCTCTGCACGCTTCAC | 153 |
| Pav_sc0000638.1_g820.1.mk | TGGCTTGTTGGGATGAAATG | GAACGAAGATGCGAAGTAGGG | 183 |
| Pav_sc0001335.1_g050.1.mk | GCTCAGCCTTTTGGTGCATAT | GCCGTACTTTGAGTCGCTTTC | 156 |
| Pav_sc0001479.1_g020.1.mk | TATTACAGGGGAAGCACGAGG | ACGTGGCAAAATCTCAGGGT | 194 |
| Pav_sc0000893.1_g020.1.mk | GGCTTGGAATGATTGTTTGGA | ACAGGTTGCTTGTTAGCGTGAC | 209 |
| Pav_sc0009842.1_g030.1.mk | GGGCTCCGAATCATGTGAAC | ATTAGGGACCCAAAGAAGAACG | 159 |
| *ACTIN* | TTGTGCTGGACTCTGGTGATG | GCTCAGCAGTGGTGGTGAAC | - |

**Table S2** Transcriptome Illumina sequencing and mapped results on DT and DS

| **Samples** | **Clean reads** | **Map rate** | **Clean bases** | **Q 30 (%)** | **GC content (%)** |
| --- | --- | --- | --- | --- | --- |
| G5_LS1 | 51800008 | 82.2% | 7.77G | 90.32 | 44.62 |
| G5_LS2 | 50585496 | 81.6% | 7.59G | 90.84 | 44.52 |
| G5_LS3 | 52872452 | 83.0% | 7.93G | 91.70 | 45.03 |
| G5_LCK1 | 52019614 | 83.1% | 7.8G | 91.02 | 45.04 |
| G5_LCK2 | 50678998 | 83.0% | 7.6G | 90.41 | 45.13 |
| G5_LCK3 | 53927314 | 83.9% | 8.09G | 91.88 | 45.27 |
| G5_RS1 | 49442254 | 79.9% | 7.42G | 91.40 | 45.48 |
| G5_RS2 | 54373868 | 80.7% | 8.16G | 92.01 | 45.51 |
| G5_RS3 | 60791002 | 79.8% | 9.12G | 91.79 | 45.66 |
| G5_RCK1 | 53644954 | 82.9% | 8.05G | 91.45 | 45.34 |
| G5_RCK2 | 48877706 | 81.7% | 7.33G | 91.08 | 45.68 |
| G5_RCK3 | 51082546 | 80.3% | 7.66G | 87.18 | 45.24 |
| CDR_LS1 | 42012262 | 80.2% | 5.25G | 94.72 | 45.76 |
| CDR_LS2 | 39414918 | 81.9% | 4.93G | 94.75 | 45.95 |
| CDR_LS3 | 52513680 | 81.0% | 6.56G | 95.03 | 46.01 |
| CDR_LCK1 | 38569344 | 80.0% | 4.82G | 93.42 | 46.24 |
| CDR_LCK2 | 53862728 | 79.3% | 6.73G | 94.88 | 46.12 |
| CDR_LCK3 | 37346652 | 79.3% | 4.67G | 93.54 | 45.70 |
| CDR_RS1 | 30852238 | 75.3% | 3.86G | 94.84 | 46.16 |
| CDR_RS2 | 39798700 | 79.1% | 4.97G | 92.98 | 46.43 |
| CDR_RS3 | 38248542 | 79.2% | 4.78G | 92.96 | 46.41 |
| CDR_RCK1 | 45153498 | 78.3% | 5.64G | 94.76 | 46.72 |
| CDR_RCK2 | 39590088 | 80.2% | 4.95G | 93.35 | 46.12 |
| CDR_RCK3 | 30943208 | 77.8% | 3.87G | 92.94 | 46.10 |

Note: CDR-RCK, CDR-LCK, G5-RCK, G5-LCK are well-watered control samples, CDR-RS, CDR-LS, G5-RS, G5-LS are drought treatment samples.

**Table S3** Common drought response transcripts between DT and DS leaves and roots

| **Gene ID** | **LS** | **LCK** | **log2FC** | **FDR** | **Putative function** |
| --- | --- | --- | --- | --- | --- |
| Pav_sc0000124.1_g140.1.mk | 1851.7 | 176.8 | 3.4 | 0.000 | transcriptional regulator IFH1 |
| Pav_sc0000176.1_g240.1.mk | 643.1 | 105.9 | 2.6 | 0.000 | ninja-family protein AFP3 |
| Pav_sc0000271.1_g230.1.mk | 967.1 | 326.6 | 1.6 | 0.045 | probable CCR4-associated factor 1 homolog 11 |
| Pav_sc0000293.1_g410.1.mk | 1223.8 | 86.6 | 3.8 | 0.000 | COL domain class transcription factor |
| Pav_sc0001124.1_g320.1.mk | 5835.5 | 234.1 | 4.6 | 0.000 | Low quality protein reveille1 |
| Pav_co4022557.1_g010.1.br | 29.8 | 131.2 | -2.1 | 0.033 | oligopeptide transporter 7-like |
| Pav_sc0000017.1_g420.1.mk | 703.4 | 2172.5 | -1.6 | 0.044 | bHLH transcription factor |
| Pav_sc0000412.1_g660.1.mk | 23.0 | 141.3 | -2.6 | 0.001 | U-box domain-containing protein 15-like |
| Pav_sc0000567.1_g740.1.mk | 782.7 | 2762.6 | -1.8 | 0.013 | AP2/ERF and B3 domain-containing transcription factor RAV1 |
| Pav_sc0001102.1_g230.1.mk | 778.6 | 2864.9 | -1.9 | 0.003 | AP2/EREBP family transcription factor-like protein 15 |
| Pav_sc0001051.1_g030.1.mk | 869.7 | 10154.9 | -3.5 | 0.000 | zinc finger protein constans6-like |
| Pav_sc0001305.1_g990.1.mk | 15.1 | 92.4 | -2.6 | 0.001 | AP2/EREBP family transcription factor-like protein 9 |
| Pav_sc0001335.1_g080.1.mk | 97.7 | 364.0 | -1.9 | 0.020 | putative RING-H2 finger protein ATL21B |
| Pav_sc0000129.1_g1080.1.mk | 246.1 | 824.3 | -1.7 | 0.014 | serine/threonine-protein kinase At5g01020 |
| Pav_sc0000254.1_g1260.1.mk | 192.9 | 1063.8 | -2.5 | 0.000 | probable serine/threonine-protein kinase At1g01540 |
| Pav_sc0000349.1_g230.1.br | 19.9 | 274.5 | -3.8 | 0.000 | lysine-rich arabinogalactan protein 18-like |
| Pav_sc0001781.1_g130.1.mk | 411.8 | 1774.4 | -2.1 | 0.002 | leucine-rich repeat receptor-like serine/threonine-protein kinase BAM1 |
| Pav_sc0003835.1_g010.1.mk | 442.7 | 1551.8 | -1.8 | 0.017 | LRR receptor-like serine/threonine-protein kinase At1g07650 |
| Pav_sc0009864.1_g010.1.mk | 14.9 | 146.5 | -3.3 | 0.008 | lysine-rich arabinogalactan protein 18 |
| Pav_sc0000877.1_g1320.1.mk | 158.4 | 551.3 | -1.8 | 0.008 | probable serine/threonine-protein kinase At1g54610 |
| Pav_sc0000582.1_g980.1.mk | 80.3 | 270.9 | -1.8 | 0.035 | bifunctional aspartokinase/homoserine dehydrogenase |
| Pav_sc0002342.1_g010.1.mk | 178.5 | 574.7 | -1.7 | 0.038 | G-type lectin S-receptor-like serine/threonine-protein kinase RLK1 |
| Pav_sc0002342.1_g050.1.mk | 96.7 | 412.7 | -2.1 | 0.003 | G-type lectin S-receptor-like serine/threonine-protein kinase RLK1 |
| Pav_sc0000129.1_g900.1.mk | 14.6 | 203.6 | -3.8 | 0.012 | auxin-binding protein ABP20 |
| Pav_sc0000983.1_g260.1.mk | 48.8 | 218.4 | -2.2 | 0.005 | auxin-induced protein AUX28-like |
| Pav_sc0004305.1_g250.1.mk | 724.1 | 4656.8 | -2.7 | 0.000 | auxin-binding protein ABP19a |
| Pav_sc0006464.1_g040.1.mk | 1031.1 | 34120.3 | -5.0 | 0.000 | probable xyloglucan endotransglucosylase/hydrolase protein 6 |
| Pav_sc0000034.1_g040.1.mk | 1100.8 | 5611.7 | -2.3 | 0.000 | probable glycerophosphoryl diester phosphodiesterase 2 |
| Pav_sc0000354.1_g310.1.mk | 98.8 | 4841.0 | -5.6 | 0.000 | probable xyloglucan endotransglucosylase/hydrolase protein 33 |
| Pav_sc0000440.1_g180.1.mk | 146.4 | 1022.3 | -2.8 | 0.000 | fasciclin-like arabinogalactan protein 17 |
| Pav_sc0000410.1_g030.1.mk | 103.6 | 488.4 | -2.2 | 0.001 | DEAD-box ATP-dependent RNA helicase 32 |
| Pav_sc0000890.1_g1520.1.mk | 60.5 | 194.1 | -1.7 | 0.044 | probable galacturonosyltransferase-like 1 |
| Pav_sc0001323.1_g970.1.mk | 88.9 | 454.2 | -2.4 | 0.001 | beta-xylosidase/alpha-L-arabinofuranosidase 2 |
| Pav_sc0001575.1_g010.1.mk | 1521.2 | 5658.6 | -1.9 | 0.010 | glucose-1-phosphate adenylyltransferase large subunit 1-like |
| Pav_sc0001519.1_g030.1.mk | 153.2 | 580.6 | -1.9 | 0.050 | probable xyloglucan glycosyltransferase 12 |
| Pav_sc0004467.1_g120.1.mk | 128.2 | 1302.3 | -3.3 | 0.015 | sugar transporter ERD6-like 16 |
| Pav_sc0000174.1_g1430.1.br | 1215.6 | 4203.0 | -1.8 | 0.009 | major allergen Pru ar 1 |
| Pav_sc0000174.1_g1770.1.br | 51.3 | 198.5 | -2.0 | 0.012 | major allergen Pru av 1-like |
| Pav_sc0000071.1_g530.1.mk | 2.3 | 48.8 | -4.4 | 0.000 | protein RALF-like 34 |
| Pav_sc0000219.1_g050.1.mk | 45.0 | 637.9 | -3.8 | 0.000 | protein plant cadmium resistance 7-like |
| Pav_sc0000554.1_g330.1.mk | 378.4 | 1671.4 | -2.1 | 0.001 | ultraviolet-B receptor UVR8 |
| Pav_sc0000652.1_g350.1.mk | 82.8 | 411.4 | -2.3 | 0.011 | oligopeptide transporter 7-like |
| **Gene ID** | **LS** | **LCK** | **log2FC** | **FDR** | **Putative function** |
| Pav_sc0000652.1_g350.1.mk | 82.8 | 411.4 | -2.3 | 0.011 | oligopeptide transporter 7-like |
| Pav_sc0000652.1_g380.1.mk | 17.1 | 124.2 | -2.9 | 0.001 | oligopeptide transporter 7-like |
| Pav_sc0000747.1_g320.1.br | 130.1 | 679.7 | -2.4 | 0.000 | general transcriptional corepressor tupA |
| Pav_sc0000852.1_g050.1.mk | 74.3 | 1294.6 | -4.1 | 0.000 | peamaclein |
| Pav_sc0000852.1_g690.1.mk | 72.8 | 365.6 | -2.3 | 0.001 | subtilisin-like protease |
| Pav_sc0000877.1_g910.1.mk | 6110.8 | 19461.8 | -1.7 | 0.034 | chlorophyll a-b binding protein P4, chloroplastic |
| Pav_sc0000893.1_g020.1.mk | 94.6 | 1342.1 | -3.8 | 0.000 | pectinesterase |
| Pav_sc0000907.1_g650.1.mk | 820.6 | 2392.3 | -1.5 | 0.036 | dnaJ homolog subfamily B member 3 |
| Pav_sc0000975.1_g210.1.mk | 83.2 | 509.9 | -2.6 | 0.000 | squamosa promoter-binding-like protein 3 |
| Pav_sc0001084.1_g460.1.mk | 37.5 | 136.3 | -1.9 | 0.022 | rho GDP-dissociation inhibitor 1 |
| Pav_sc0001118.1_g030.1.mk | 272.7 | 1476.2 | -2.4 | 0.000 | formin-like protein 1 |
| Pav_sc0003286.1_g010.1.mk | 9.7 | 56.8 | -2.5 | 0.012 | sulfate transporter 2.1-like |
| Pav_sc0004547.1_g060.1.br | 171.1 | 633.9 | -1.9 | 0.010 | receptor-like protein kinase FERONIA |
| Pav_sc0000685.1_g080.1.mk | 141.2 | 446.2 | -1.7 | 0.027 | probable receptor protein kinase TMK1 |
| Pav_sc0001181.1_g1090.1.mk | 255.5 | 748.8 | -1.6 | 0.041 | chaperone protein dnaJ 6 |
| Pav_sc0001191.1_g110.1.mk | 367.9 | 1157.5 | -1.7 | 0.027 | cytochrome P450 CYP749A22-like |
| Pav_sc0001502.1_g290.1.mk | 41.7 | 165.2 | -2.0 | 0.017 | cationic peroxidase 1-like |
| Pav_sc0001126.1_g030.1.mk | 8.7 | 207.5 | -4.6 | 0.001 | uncharacterized protein At4g06744-like |
| Pav_sc0000136.1_g020.1.br | 28.2 | 485.0 | -4.1 | 0.000 | uncharacterized protein At4g06744-like |
| Pav_sc0002234.1_g100.1.mk | 458.1 | 1217.8 | -1.4 | 0.045 | uncharacterized protein LOC103335357 |
| Pav_sc0001197.1_g100.1.mk | 256.6 | 62.1 | 2.0 | 0.005 | uncharacterized protein LOC103323789 |
| Pav_sc0002401.1_g330.1.mk | 12805.5 | 1389.5 | 3.2 | 0.000 | uncharacterized serine-rich protein C215.13-like |
| Pav_sc0000138.1_g440.1.mk | 314.4 | 68.2 | 2.2 | 0.001 | uncharacterized calcium-binding protein At1g02270 |
| Pav_sc0000212.1_g1130.1.mk | 12.0 | 428.2 | -5.2 | 0.000 | hypothetical protein-ppa006267mg |
| Pav_sc0000405.1_g550.1.mk | 8.1 | 41.8 | -2.4 | 0.046 | hypothetical protein_ppa014957mg |
| Pav_sc0000567.1_g470.1.mk | 25.6 | 111.0 | -2.1 | 0.017 | hypothetical protein B456_004G198900 |
| Pav_sc0000719.1_g550.1.mk | 805.5 | 2576.4 | -1.7 | 0.033 | hypothetical protein VITISV_040328 |
| Pav_sc0001217.1_g090.1.mk | 1840.5 | 6585.1 | -1.8 | 0.005 | hypothetical protein PRUPE_ppa009922mg |
| Pav_sc0001405.1_g780.1.mk | 582.2 | 1983.7 | -1.8 | 0.018 | hypothetical protein PRUPE_ppa013533mg |
| Novel01458 | 724.7 | 2621.5 | -1.9 | 0.011 | hypothetical protein PRUPE_ppa000698mg [Prunpersica]>gi\|462411063\|gb\|EMJ16112.1\| hypothetical protein PRUPE_ppa000698mg [Prunus persica] |
| Pav_sc0002001.1_g060.1.mk | 3.5 | 24.4 | -2.8 | 0.050 | unnamed protein product |
| Pav_sc0000767.1_g130.1.mk | 853.7 | 3878.4 | -2.2 | 0.000 | - |
| Pav_sc0000052.1_g450.1.br | 3778.3 | 267.6 | 3.8 | 0.005 | extensin-3-like |
| Pav_sc0000103.1_g1100.1.mk | 323.5 | 98.3 | 1.7 | 0.017 | U-box domain-containing protein 21-like |
| Pav_sc0000557.1_g1350.1.mk | 287.5 | 85.7 | 1.7 | 0.027 | chitotriosidase-1-like |
| Pav_sc0000030.1_g1480.1.mk | 116.5 | 845.9 | -2.9 | 0.000 | ethylene-responsive transcription factor ERF012-like |
| Pav_sc0000129.1_g1460.1.mk | 2.1 | 229.5 | -6.8 | 0.001 | ethylene-responsive transcription factor ERF017 |
| Pav_sc0000107.1_g560.1.br | 0.7 | 83.6 | -6.9 | 0.020 | CBF/DREB1-like protein |
| Pav_sc0000480.1_g980.1.br | 102.5 | 453.3 | -2.1 | 0.000 | probable WRKY transcription factor 30 |
| Pav_sc0000800.1_g060.1.mk | 220.3 | 878.0 | -2.0 | 0.037 | transcription factor MYB44-like |
| Pav_sc0002451.1_g020.1.mk | 3.1 | 633.5 | -7.7 | 0.002 | AP2/EREBP family transcription factor-like protein 11 |
| Pav_sc0000009.1_g1290.1.br | 247.2 | 1343.6 | -2.4 | 0.000 | probable galacturonosyltransferase-like 10 |
| Pav_sc0000009.1_g970.1.mk | 321.0 | 759.0 | -1.2 | 0.042 | xyloglucan glycosyltransferase 4 |
| **Gene ID** | **LS** | **LCK** | **log2FC** | **FDR** | **Putative function** |
| Pav_sc0000009.1_g970.1.mk | 321.0 | 759.0 | -1.2 | 0.042 | xyloglucan glycosyltransferase 4 |
| Pav_sc0000428.1_g510.1.mk | 59.0 | 866.2 | -3.9 | 0.014 | brassinosteroid-regulated protein BRU1-like |
| Pav_sc0000428.1_g530.1.mk | 47.6 | 483.6 | -3.3 | 0.013 | brassinosteroid-regulated protein BRU1-like |
| Pav_sc0000428.1_g560.1.mk | 35.2 | 223.4 | -2.7 | 0.042 | brassinosteroid-regulated protein BRU1-like |
| Pav_sc0000260.1_g250.1.mk | 170.0 | 587.8 | -1.8 | 0.021 | calmodulin-like protein 3 |
| Pav_sc0000441.1_g110.1.mk | 87.6 | 938.6 | -3.4 | 0.002 | calcium-binding protein PBP1-like |
| Pav_sc0000464.1_g670.1.mk | 145.3 | 598.8 | -2.0 | 0.000 | probable calcium-binding protein CML23 |
| Pav_sc0000491.1_g1020.1.mk | 76.3 | 256.1 | -1.7 | 0.005 | chitinase 10 |
| Pav_sc0000554.1_g980.1.mk | 341.9 | 943.4 | -1.5 | 0.006 | glucan endo-1,3-beta-glucosidase-like |
| Pav_sc0000129.1_g330.1.mk | 211.1 | 2860.3 | -3.8 | 0.023 | putative nuclease HARBI1 |
| Pav_sc0003468.1_g110.1.mk | 131.1 | 364.4 | -1.5 | 0.013 | probable carboxylesterase 15 |
| **Pav_sc0000257.1_g1040.1.mk** | **223.9** | **1966.0** | **-3.1** | **0.000** | **mitochondrial uncoupling protein 5-like** |
| **Pav_sc0001700.1_g110.1.mk** | **2.0** | **319.3** | **-7.3** | **0.001** | **mitochondrial uncoupling protein 4** |
| Novel01512 | 21.7 | 248.8 | -3.5 | 0.010 | CYSTM1 family protein A-like |
| Pav_sc0001229.1_g040.1.br | 3.4 | 29.7 | -3.1 | 0.002 | probable disease resistance RPP8-like protein 2 |
| Pav_sc0012430.1_g010.1.mk | 93.3 | 1208.7 | -3.7 | 0.002 | calcium-binding protein PBP1-like |
| Pav_sc0000689.1_g480.1.mk | 199.8 | 747.5 | -1.9 | 0.001 | probable LIM domain-containing serine/threonine-protein kinase DDB_G0286997 |
| Pav_sc0001192.1_g170.1.br | 76.4 | 326.9 | -2.1 | 0.000 | classical arabinogalactan protein 1-like |
| Pav_sc0000257.1_g060.1.mk | 134.7 | 1025.4 | -2.9 | 0.001 | hypothetical protein_ppa008424mg |
| Pav_sc0002360.1_g030.1.mk | 59.7 | 201.6 | -1.8 | 0.018 | hypothetical protein L484_003489 |
| Pav_sc0002837.1_g050.1.mk | 322.8 | 940.6 | -1.5 | 0.004 | hypothetical protein POPTR_0016s05620g, partial |
| Pav_sc0003562.1_g350.1.mk | 6.3 | 80.2 | -3.7 | 0.000 | hypothetical protein PRUPE_ppa017712mg |
| Pav_sc0000405.1_g550.1.mk | 25.2 | 146.8 | -2.5 | 0.024 | hypothetical protein_ppa014957mg |
| Pav_sc0000600.1_g350.1.mk | 81.3 | 653.4 | -3.0 | 0.017 | hypothetical protein POPTR_0005s28050g |
| Pav_sc0000852.1_g720.1.mk | 564.3 | 1309.8 | -1.2 | 0.046 | hypothetical protein PRUPE_ppa008855mg |
| Pav_sc0000893.1_g1090.1.mk | 10.6 | 68.1 | -2.7 | 0.022 | hypothetical protein PRUPE_ppa026515mg |
| Pav_sc0000893.1_g310.1.mk | 84.8 | 226.6 | -1.4 | 0.031 | probable calcium-binding protein CML25 |
| Pav_sc0000998.1_g350.1.br | 19.0 | 78.7 | -2.1 | 0.005 | hypothetical protein GLYMA_20G204500 |
| Pav_sc0001040.1_g260.1.mk | 60.2 | 189.3 | -1.7 | 0.006 | uncharacterized protein At1g04910-like |
| Pav_sc0001181.1_g690.1.mk | 249.7 | 2199.9 | -3.1 | 0.013 | uncharacterized protein LOC103341559 |
| Pav_sc0001181.1_g790.1.mk | 113.8 | 301.7 | -1.4 | 0.031 | uncharacterized protein LOC103341568 |
| Pav_sc0001203.1_g150.1.mk | 428.9 | 1000.5 | -1.2 | 0.045 | uncharacterized protein LOC103330641 |
| Pav_sc0000130.1_g360.1.mk | 10.2 | 90.7 | -3.2 | 0.011 | uncharacterized protein LOC103334034 |
| Pav_sc0003567.1_g050.1.br | 7.8 | 82.4 | -3.4 | 0.044 | - |
| Pav_sc0000146.1_g520.1.mk | 27.8 | 132.5 | -2.3 | 0.000 | - |
| Pav_sc0000257.1_g050.1.mk | 619.4 | 1592.4 | -1.4 | 0.024 | - |
| Pav_sc0000257.1_g1050.1.br | 31.1 | 336.2 | -3.4 | 0.000 | - |
| Pav_sc0002482.1_g030.1.br | 15.2 | 112.3 | -2.9 | 0.001 | - |

**Table S4** Highly induced and depressed unique transcripts in DT leaves

| **Gene ID** | **LS** | **LCK** | **log2FC** | **FDR** | **Putative function** |
| --- | --- | --- | --- | --- | --- |
| Pav_sc0000069.1_g640.1.mk | 37.0 | 2.1 | 4.1 | 0.008 | glutaredoxin-C6 |
| Pav_sc0001405.1_g1920.1.mk | 22.7 | 1.4 | 4.0 | 0.004 | G-type lectin S-receptor-like serine/threonine-protein kinase At5g24080 |
| Pav_sc0001974.1_g090.1.mk | 53.8 | 2.4 | 4.5 | 0.000 | Low quality protein 18 kDa seed maturation protein |
| Pav_sc0004687.1_g090.1.mk | 87.3 | 5.2 | 4.1 | 0.000 | hypothetical protein PRUPE_ppa020510mg |
| Pav_co4053553.1_g010.1.mk | 124.6 | 8.4 | 3.9 | 0.013 | low-temperature-induced 65 kDa protein-like |
| Pav_sc0001405.1_g970.1.mk | 226.0 | 26.9 | 3.1 | 0.034 | low-temperature-induced 65 kDa protein-like |
| Pav_sc0000040.1_g120.1.mk | 1860.2 | 220.9 | 3.1 | 0.019 | probable galactinol--sucrose galactosyltransferase 5 |
| Pav_sc0000491.1_g270.1.mk | 327.0 | 38.1 | 3.1 | 0.000 | stachyose synthase-like |
| Pav_sc0006061.1_g110.1.mk | 14032.1 | 1352.9 | 3.4 | 0.000 | beta-amylase 3, chloroplastic |
| Pav_sc0001003.1_g160.1.mk | 49.7 | 5.5 | 3.2 | 0.000 | probable purine permease 4 |
| Pav_sc0000261.1_g020.1.mk | 21.3 | 1.4 | 3.9 | 0.006 | GDSL esterase/lipase At3g48460 |
| Pav_sc0000909.1_g530.1.mk | 60.0 | 5.7 | 3.4 | 0.000 | protein YLS3-like |
| Pav_sc0000308.1_g360.1.mk | 2428.6 | 223.6 | 3.4 | 0.004 | anthocyanidin 3-O-glucosyltransferase 7-like |
| Pav_sc0000747.1_g460.1.br | 38.8 | 4.8 | 3.0 | 0.007 | cytochrome P450 71B37-like |
| Pav_sc0000766.1_g240.1.mk | 191.4 | 20.9 | 3.2 | 0.004 | hypothetical protein PRUPE_ppa027069mg |
| Pav_sc0000852.1_g880.1.mk | 584.4 | 59.3 | 3.3 | 0.000 | hypothetical protein PRUPE_ppa010429mg |
| Novel00768 | 24.6 | 2.7 | 3.2 | 0.008 | hypothetical protein PRUPE_ppa023458mg (Prunus persica) persica]> gi\|462421460\|gb\|EMJ25723.1\| hypothetical protein PRUPE_ppa023458 mg [Prunus persica] |
| Pav_sc0000449.1_g130.1.mk | 47.6 | 2.2 | 4.4 | 0.047 | hypothetical protein CISIN_1g040585mg, partial |
| Pav_sc0000293.1_g490.1.mk | 20.9 | 0.3 | 6.3 | 0.001 | uncharacterized protein LOC103327421 isoform X1 |
| Pav_sc0000554.1_g420.1.mk | 15.1 | 0.5 | 4.8 | 0.024 | uncharacterized protein LOC103332685 |
| Pav_sc0001175.1_g090.1.mk | 32.9 | 0.3 | 6.9 | 0.028 | uncharacterized protein LOC103340063 |
| Pav_sc0001509.1_g140.1.br | 21.4 | 1.2 | 4.2 | 0.003 | uncharacterized protein LOC103322889 |
| Pav_sc0004348.1_g250.1.mk | 58.8 | 4.3 | 3.8 | 0.001 | uncharacterized protein LOC103330474 |
| Pav_sc0000502.1_g400.1.mk | 3.6 | 40.0 | -3.5 | 0.000 | ethylene-responsive transcription factor ERF020 |
| Pav_sc0000129.1_g1460.1.mk | 38.0 | 428.2 | -3.5 | 0.000 | ethylene-responsive transcription factor ERF017 |
| Pav_sc0000355.1_g370.1.mk | 3.9 | 68.0 | -4.1 | 0.014 | ethylene-responsive transcription factor ERF027-like |
| Pav_sc0000428.1_g520.1.mk | 3.0 | 33.1 | -3.4 | 0.049 | brassinosteroid-regulated protein BRU1-like |
| Pav_sc0000428.1_g560.1.mk | 1.4 | 30.7 | -4.4 | 0.001 | brassinosteroid-regulated protein BRU1-like |
| Pav_sc0000757.1_g020.1.br | 33.7 | 285.5 | -3.1 | 0.000 | LRR receptor-like serine/threonine-protein kinase GSO1 |
| Pav_sc0000852.1_g350.1.mk | 1.2 | 26.3 | -4.4 | 0.001 | putative receptor protein kinase ZmPK1 |
| Pav_sc0000977.1_g510.1.mk | 102.8 | 1041.9 | -3.3 | 0.027 | cytochrome P450 94C1 |
| Pav_sc0002360.1_g910.1.mk | 4.9 | 44.6 | -3.2 | 0.000 | myb-related protein Myb4-like |
| **Pav_sc0003802.1_g110.1.mk** | **586.5** | **5608.3** | **-3.3** | **0.000** | **dehydration-responsive protein RD22-like** |
| **Pav_sc0006347.1_g080.1.br** | **1.6** | **16.5** | **-3.4** | **0.043** | **dehydration-responsive protein RD22-like** |
| **Pav_sc0011243.1_g010.1.mk** | **10.4** | **86.1** | **-3.0** | **0.001** | **dehydration-responsive protein RD22-like** |
| **Pav_sc0011423.1_g010.1.br** | **46.2** | **455.1** | **-3.3** | **0.003** | **dehydration-responsive protein RD22-like** |
| **Pav_sc0012602.1_g010.1.mk** | **70.1** | **680.3** | **-3.3** | **0.000** | **dehydration-responsive protein RD22-like** |
| Pav_sc0000396.1_g640.1.mk | 12.3 | 438.3 | -5.2 | 0.001 | ethylene-responsive transcription factor 1B-like |
| Pav_sc0000583.1_g510.1.mk | 3.2 | 87.1 | -4.8 | 0.004 | ethylene-responsive transcription factor 1B-like |
| **Gene ID** | **LS** | **LCK** | **log2FC** | **FDR** | **Putative function** |
| Pav_sc0000119.1_g160.1.mk | 28.4 | 639.7 | -4.5 | 0.000 | ethylene-responsive transcription factor 1B |
| **Pav_sc0003681.1_g010.1.mk** | **6.3** | **87.5** | **-3.8** | **0.015** | **probable alpha,alpha-trehalose-phosphate synthase** |
| **Novel01202** | **23.1** | **244.4** | **-3.4** | **0.035** | **receptor-like protein kinase [Prunus mume]** |
| Pav_sc0000852.1_g310.1.br | 5.3 | 43.0 | -3.0 | 0.001 | putative receptor protein kinase ZmPK1 |
| Pav_sc0000622.1_g170.1.br | 21.8 | 230.8 | -3.4 | 0.032 | receptor-like protein 12 |
| Pav_sc0000854.1_g110.1.br | 5.1 | 54.4 | -3.4 | 0.013 | receptor-like protein 12 |
| Pav_sc0002060.1_g020.1.br | 8.5 | 83.3 | -3.3 | 0.027 | probable receptor-like protein kinase At5g3902  0 |
| Pav_sc0000886.1_g770.1.mk | 10.7 | 184.2 | -4.1 | 0.004 | probable WRKY transcription factor 29 |
| Pav_sc0002451.1_g070.1.mk | 1.9 | 17.8 | -3.2 | 0.041 | probable F-box protein At4g22030 |
| Pav_sc0007588.1_g010.1.mk | 115.0 | 1045.7 | -3.2 | 0.000 | F-box protein At2g27310-like |
| **Pav_sc0001502.1_g260.1.br** | **7.0** | **105.3** | **-3.9** | **0.016** | **cationic peroxidase 1-like** |
| **Pav_sc0001299.1_g090.1.mk** | **16.2** | **244.0** | **-3.9** | **0.000** | **annexin D4** |
| **Novel01659** | **3.8** | **56.4** | **-3.9** | **0.000** | **annexin D4 [Prunus mume]** |
| **Pav_sc0011889.1_g010.1.br** | **4.3** | **52.4** | **-3.6** | **0.000** | **metalloendoproteinase 1-like** |
| **Pav_co4060979.1_g010.1.mk** | **6.8** | **84.0** | **-3.6** | **0.001** | **arginase 1, mitochondrial** |
| Pav_sc0000037.1_g350.1.mk | 15.1 | 135.5 | -3.2 | 0.000 | heavy metal-associated isoprenylated plant protein 26 |
| Pav_sc0004531.1_g060.1.br | 2.2 | 108.7 | -5.6 | 0.000 | 14 kDa proline-rich protein DC2.15-like |
| **Pav_sc0000057.1_g050.1.mk** | **16.6** | **262.6** | **-4.0** | **0.001** | **abscisic acid receptor PYL4-like** |
| **Pav_sc0001341.1_g250.1.mk** | **57.8** | **763.1** | **-3.7** | **0.000** | **abscisic acid receptor PYL4** |
| **Pav_sc0000099.1_g330.1.mk** | **22.2** | **186.4** | **-3.1** | **0.000** | **putative cell division cycle ATPase** |
| Pav_sc0006144.1_g040.1.br | 1.5 | 19.1 | -3.6 | 0.021 | lysine histidine transporter-like 8 |
| Pav_sc0000131.1_g390.1.mk | 2.1 | 39.9 | -4.2 | 0.000 | 3-ketoacyl-CoA synthase 10 |
| Pav_sc0000174.1_g1900.1.mk | 19.5 | 212.6 | -3.4 | 0.000 | major allergen Pru av 1-like |
| Pav_sc0000618.1_g380.1.mk | 150.5 | 1391.4 | -3.2 | 0.005 | allene oxide cyclase 4, chloroplastic-like |
| Pav_sc0000700.1_g1700.1.mk | 3.4 | 44.5 | -3.7 | 0.025 | pollen-specific protein SF21-like |
| Novel01344 | 7.4 | 109.6 | -3.9 | 0.000 | hypothetical protein PRUPE_ppa000601mg [Prunus persica]>gi \|462417374\|gb\|EMJ22111.1\| hypothetical protein PRUPE_ppa000601mg [Prunus persica] |
| Pav_co3996249.1_g010.1.br | 3.8 | 30.9 | -3.0 | 0.007 | patatin-like protein 2 |
| Pav_co4036755.1_g010.1.br | 7.0 | 155.7 | -4.5 | 0.000 | uncharacterized protein At4g06744-like |
| Pav_sc0000373.1_g260.1.mk | 92.2 | 835.1 | -3.2 | 0.000 | uncharacterized protein LOC103334808 |
| Pav_sc0000157.1_g640.1.mk | 18.0 | 162.7 | -3.2 | 0.026 | -\- |
| Pav_sc0000789.1_g220.1.mk | 3.1 | 29.8 | -3.2 | 0.003 | -\- |
| Pav_sc0001327.1_g040.1.mk | 3.9 | 305.0 | -6.3 | 0.000 | L-type lectin-domain containing receptor kinase IX.1-like |
| Pav_sc0001405.1_g1090.1.mk | 2.2 | 784.0 | -8.5 | 0.001 | cytochrome P450 714C2-like |
| Pav_sc0001615.1_g030.1.mk | 0.3 | 144.5 | -9.0 | 0.000 | galactolipase DONGLE, chloroplastic |
| Pav_sc0001938.1_g090.1.br | 1.2 | 135.4 | -6.8 | 0.000 | peroxidase 5-like |
| Pav_sc0002119.1_g040.1.mk | 0.3 | 52.2 | -7.5 | 0.000 | methanol O-anthraniloyltransferase-like |
| **Pav_sc0002270.1_g200.1.br** | **0.3** | **25.9** | **-6.5** | **0.001** | **receptor-like protein 12** |
| Pav_sc0003135.1_g030.1.mk | 141.5 | 10060.4 | -6.2 | 0.000 | phospholipase A1-Igamma1, chloroplastic |
| Pav_sc0008941.1_g010.1.br | 0.3 | 27.2 | -6.6 | 0.000 | TMV resistance protein N-like |
| Pav_sc0011444.1_g010.1.mk | 9.1 | 702.1 | -6.3 | 0.011 | TMV resistance protein N-like |
| Pav_sc0000293.1_g320.1.br | 1.9 | 148.1 | -6.3 | 0.000 | -\- |
| **Gene ID** | **LS** | **LCK** | **log2FC** | **FDR** | **Putative function** |
| Pav_sc0000129.1_g1190.1.mk | 1.3 | 146.8 | -6.8 | 0.017 | -\- |
| Pav_sc0001327.1_g010.1.br | 0.3 | 22.2 | -6.2 | 0.002 | -\- |
| Pav_sc0004652.1_g040.1.br | 2.7 | 195.3 | -6.2 | 0.000 | -\- |

**Table S5** Highly induced and repressed unique transcripts in DT roots

| **Gene ID** | **RS** | **LCK** | **log2FC** | **FDR** | **Putative function** |
| --- | --- | --- | --- | --- | --- |
| Pav_sc0000069.1_g640.1.mk | 154.1 | 12.0 | 3.7 | 0.008 | glutaredoxin-C6 |
| Pav_sc0001405.1_g1920.1.mk | 204.2 | 4.8 | 5.4 | 0.020 | G-type lectin S-receptor-like serine/threonine-protein kinase At5g24080 |
| Pav_sc0001974.1_g090.1.mk | 104.6 | 10.7 | 3.3 | 0.009 | 18 kDa seed maturation protein |
| Pav_sc0004687.1_g090.1.mk | 628.3 | 3.8 | 7.4 | 0.001 | hypothetical protein PRUPE_ppa020510mg |
| Novel00309 | 538.3 | 39.8 | 3.8 | 0.032 | hypothetical protein PRUPE_ppa017942mg |
| Pav_sc0001958.1_g050.1.mk | 165.4 | 5.5 | 4.9 | 0.002 | hypothetical protein PRUPE_ppa015056mg |
| Pav_sc0000039.1_g150.1.mk | 450.4 | 6.8 | 6.1 | 0.002 | hypothetical protein PRUPE_ppa015056mg |
| Pav_sc0000376.1_g720.1.mk | 38.5 | 2.1 | 4.2 | 0.002 | hypothetical protein PRUPE_ppa022014mg |
| Pav_sc0003135.1_g430.1.mk | 416.5 | 51.1 | 3.0 | 0.024 | hypothetical protein PRUPE_ppa017601mg |
| Pav_sc0000024.1_g170.1.mk | 56.9 | 6.6 | 3.1 | 0.027 | uncharacterized protein LOC103324482 |
| Pav_sc0000624.1_g1150.1.mk | 202.2 | 6.5 | 5.0 | 0.005 | Uncharacterized protein TCM_039609 |
| Pav_sc0000704.1_g390.1.mk | 27.4 | 1.8 | 3.9 | 0.036 | uncharacterized protein LOC103341044 |
| Pav_sc0004659.1_g020.1.br | 94.6 | 1.7 | 5.8 | 0.024 | uncharacterized protein LOC103322894 |
| Pav_sc0000129.1_g190.1.mk | 33.6 | 2.7 | 3.6 | 0.015 | heat stress transcription factor A-2-like |
| Pav_sc0000293.1_g410.1.mk | 389.6 | 42.8 | 3.2 | 0.000 | COL domain class transcription factor |
| Pav_sc0000308.1_g080.1.mk | 68.7 | 5.5 | 3.6 | 0.010 | (R)-mandelonitrile lyase 1-like |
| Pav_sc0000464.1_g820.1.mk | 1559.2 | 83.7 | 4.2 | 0.001 | U-box domain-containing protein 4 |
| Pav_sc0000661.1_g440.1.mk | 68.5 | 3.5 | 4.3 | 0.000 | myb-related protein 305 |
| Pav_sc0000744.1_g160.1.mk | 706.0 | 23.7 | 4.9 | 0.000 | myb-like protein X |
| Pav_sc0002001.1_g030.1.mk | 291.8 | 24.0 | 3.6 | 0.000 | RING-H2 finger protein ATL52-like |
| Pav_sc0002858.1_g200.1.mk | 3712.2 | 312.9 | 3.6 | 0.001 | probable protein phosphatase 2C 24 |
| Pav_sc0001335.1_g050.1.mk | 2026.3 | 108.2 | 4.2 | 0.005 | probable protein phosphatase 2C 51 |
| Pav_sc0000848.1_g900.1.mk | 944.6 | 10.9 | 6.4 | 0.027 | translocator protein homolog |
| Pav_sc0001073.1_g130.1.mk | 315.7 | 27.2 | 3.5 | 0.033 | septum-promoting GTP-binding protein 1 |
| Pav_sc0001305.1_g820.1.mk | 458.8 | 2.7 | 7.4 | 0.034 | late embryogenesis abundant protein 1-like |
| Pav_sc0001341.1_g190.1.mk | 1027.2 | 76.4 | 3.7 | 0.006 | sodium/potassium/calcium exchanger 1 isoform X1 |
| Pav_sc0001671.1_g140.1.mk | 27.3 | 2.6 | 3.4 | 0.027 | protein FANTASTIC FOUR 3 |
| Pav_sc0000129.1_g1460.1.mk | 15.5 | 1430.6 | -6.5 | 0.000 | ethylene-responsive transcription factor ERF017 |
| Pav_sc0000355.1_g370.1.mk | 1.5 | 879.8 | -9.2 | 0.000 | ethylene-responsive transcription factor ERF027-like |
| Pav_sc0000428.1_g520.1.mk | 26.7 | 2094.0 | -6.3 | 0.000 | brassinosteroid-regulated protein BRU1-like |
| Pav_sc0000428.1_g560.1.mk | 15.6 | 2390.8 | -7.3 | 0.000 | brassinosteroid-regulated protein BRU1-like |
| Pav_sc0000502.1_g400.1.mk | 2.1 | 393.2 | -7.6 | 0.000 | ethylene-responsive transcription factor ERF020 |
| Pav_sc0000757.1_g020.1.br | 35.1 | 2928.8 | -6.4 | 0.000 | LRR receptor-like serine/threonine-protein kinase GSO1 |
| Pav_sc0000852.1_g350.1.mk | 1.7 | 278.8 | -7.4 | 0.000 | putative receptor protein kinase ZmPK1 |
| Pav_sc0000977.1_g510.1.mk | 70.7 | 12957.9 | -7.5 | 0.000 | cytochrome P450 94C1 |
| Pav_sc0002360.1_g910.1.mk | 1.8 | 124.0 | -6.1 | 0.000 | myb-related protein Myb4-like |
| Novel01275 | 0.3 | 23.1 | -6.3 | 0.002 | hypothetical protein PRUPE_ppa023897mg |
| Pav_sc0000540.1_g210.1.mk | 0.3 | 43.5 | -7.3 | 0.000 | hypothetical protein PRUPE_ppa016272mg |
| Pav_co4048231.1_g010.1.br | 18.5 | 2745.5 | -7.2 | 0.006 | hypothetical protein PRUPE_ppa022793mg |
| Pav_sc0000071.1_g560.1.mk | 5.9 | 676.3 | -6.8 | 0.000 | hypothetical protein PRUPE_ppa005157mg |
| **Gene ID** | **RS** | **LCK** | **log2FC** | **FDR** | **Putative function** |
| Pav_sc0000293.1_g330.1.mk | 3.1 | 392.3 | -7.0 | 0.000 | hypothetical protein L484_001138 |
| Pav_sc0000890.1_g1330.1.mk | 5.4 | 471.5 | -6.4 | 0.039 | hypothetical protein PRUPE_ppa022051mg |
| Pav_sc0000910.1_g430.1.mk | 6.0 | 439.1 | -6.2 | 0.000 | hypothetical protein PRUPE_ppa026275mg |
| Pav_sc0001453.1_g020.1.mk | 25.9 | 6752.0 | -8.0 | 0.000 | hypothetical protein PRUPE_ppa014951mg, partial |
| Pav_sc0003094.1_g120.1.br | 87.1 | 5930.3 | -6.1 | 0.000 | hypothetical protein PRUPE_ppa019661mg |
| Pav_sc0002635.1_g050.1.mk | 13.7 | 1118.0 | -6.4 | 0.000 | hypothetical protein B456_011G137700 |
| Pav_sc0000800.1_g1600.1.mk | 0.3 | 22.2 | -6.2 | 0.009 | uncharacterized protein LOC103320155 |
| Pav_sc0000130.1_g360.1.mk | 6.0 | 522.9 | -6.4 | 0.000 | uncharacterized protein LOC103334034 |
| Pav_sc0000869.1_g580.1.br | 34.7 | 2627.8 | -6.2 | 0.000 | uncharacterized protein LOC103337706 |
| Pav_sc0000886.1_g880.1.br | 11.5 | 958.9 | -6.4 | 0.000 | uncharacterized protein LOC103337710 |
| Pav_sc0000359.1_g040.1.mk | 42.1 | 8008.2 | -7.6 | 0.000 | unnamed protein product |
| Pav_sc0001015.1_g120.1.mk | 15.5 | 1334.6 | -6.4 | 0.000 | uncharacterized protein LOC103319705 |
| Pav_sc0001181.1_g160.1.mk | 24.0 | 1752.6 | -6.2 | 0.000 | uncharacterized protein LOC103341531 |
| Pav_sc0002004.1_g130.1.mk | 7.1 | 617.5 | -6.4 | 0.000 | uncharacterized protein LOC103331352 |
| Pav_sc0002741.1_g090.1.mk | 46.8 | 4355.9 | -6.5 | 0.000 | uncharacterized protein LOC103334067 isoform X1 |
| Pav_sc0001405.1_g520.1.mk | 27.1 | 2018.2 | -6.2 | 0.000 | ethylene-responsive transcription factor ERF027 |
| Pav_sc0009842.1_g030.1.mk | 3.3 | 389.9 | -6.9 | 0.000 | ethylene-responsive transcription factor 13-like |
| Pav_sc0001557.1_g160.1.mk | 2.7 | 361.7 | -7.0 | 0.000 | ethylene-responsive transcription factor 1A-like |
| Pav_sc0000502.1_g250.1.mk | 1.9 | 858.5 | -8.8 | 0.000 | ethylene-responsive transcription factor ERF022-like |
| Pav_sc0000308.1_g610.1.mk | 130.2 | 11252.8 | -6.4 | 0.000 | probable xyloglucan endotransglucosylase/hydrolase protein 23 |
| Pav_sc0002360.1_g920.1.mk | 3.4 | 1897.6 | -9.1 | 0.000 | myb-related protein Myb4-like |
| Pav_sc0000890.1_g480.1.mk | 14.6 | 1438.7 | -6.6 | 0.000 | probable WRKY transcription factor 40 |
| Pav_sc0000981.1_g210.1.mk | 304.6 | 23188.6 | -6.3 | 0.000 | probable WRKY transcription factor 40 |
| Pav_sc0000893.1_g650.1.mk | 80.6 | 5269.0 | -6.0 | 0.000 | U-box domain-containing protein 21-like |
| Pav_sc0000998.1_g640.1.mk | 10.0 | 1804.8 | -7.5 | 0.000 | transcription factor bHLH92 |
| Pav_sc0001313.1_g380.1.mk | 0.8 | 416.9 | -9.1 | 0.000 | transcription factor bHLH36-like |
| Pav_sc0000055.1_g430.1.mk | 3.2 | 296.9 | -6.5 | 0.000 | NAC domain-containing protein 90-like |
| Pav_sc0000168.1_g110.1.mk | 11.9 | 2603.4 | -7.8 | 0.000 | NAC domain-containing protein 90 |
| Pav_sc0000107.1_g520.1.br | 2.8 | 1451.0 | -9.0 | 0.000 | CBF/DREB1-like protein d |
| Pav_sc0000326.1_g500.1.mk | 4.2 | 1275.5 | -8.2 | 0.000 | probable F-box protein At4g22030 |
| Pav_sc0000780.1_g050.1.mk | 71.6 | 7307.1 | -6.7 | 0.000 | zinc finger protein ZAT11 |
| Pav_sc0000352.1_g030.1.mk | 0.3 | 35.8 | -6.9 | 0.000 | probable protein phosphatase 2C 55 |
| Pav_sc0000909.1_g590.1.mk | 13.6 | 1351.2 | -6.6 | 0.000 | T4.17 |
| Pav_sc0001181.1_g340.1.mk | 20.6 | 1753.9 | -6.4 | 0.000 | probable serine/threonine-protein kinase At1g18390 |
| Pav_sc0001323.1_g410.1.mk | 35.1 | 4395.4 | -7.0 | 0.000 | serine/threonine-protein kinase-like protein CCR4 |
| Pav_sc0005527.1_g090.1.mk | 0.6 | 82.9 | -7.2 | 0.001 | tryptophan synthase alpha chain-like |
| Pav_co4034817.1_g010.1.br | 0.9 | 200.9 | -7.8 | 0.000 | probable disease resistance protein At5g66900 |
| Pav_sc0000348.1_g030.1.br | 3.6 | 692.7 | -7.6 | 0.000 | probable disease resistance protein At5g66900 |
| Pav_sc0001242.1_g030.1.mk | 0.7 | 57.3 | -6.4 | 0.000 | probable disease resistance protein At5g66900 |
| Pav_sc0002706.1_g010.1.mk | 88.6 | 9.1 | 3.3 | 0.036 | (R)-mandelonitrile lyase 1-like |
| Pav_sc0001020.1_g030.1.mk | 56.1 | 5124.9 | -6.5 | 0.000 | E3 ubiquitin-protein ligase PUB23-like |
| Pav_sc0000129.1_g330.1.mk | 432.9 | 35727.4 | -6.4 | 0.000 | putative nuclease HARBI1 |
| Pav_sc0000216.1_g580.1.mk | 1.1 | 318.8 | -8.2 | 0.000 | peroxidase N1-like |
| **Gene ID** | **RS** | **LCK** | **log2FC** | **FDR** | **Putative function** |
| Pav_sc0000333.1_g240.1.mk | 0.6 | 54.9 | -6.5 | 0.000 | terpene synthase |
| Pav_sc0001644.1_g010.1.br | 0.3 | 51.4 | -7.5 | 0.000 | trans-resveratrol di-O-methyltransferase-like |
| Pav_sc0002045.1_g020.1.mk | 0.3 | 128.8 | -8.8 | 0.000 | trans-resveratrol di-O-methyltransferase-like |
| Pav_sc0004730.1_g070.1.mk | 2.8 | 187.4 | -6.1 | 0.000 | putative auxin-responsive family protein, partial |
| Pav_sc0000063.1_g110.1.mk | 35.7 | 3668.7 | -6.7 | 0.000 | putative calcium-binding protein CML19 isoform X1 |
| Pav_sc0000127.1_g030.1.mk | 6.7 | 492.0 | -6.2 | 0.000 | Calmodulin-binding family protein |
| Pav_sc0003135.1_g450.1.mk | 22.7 | 3287.5 | -7.2 | 0.000 | protein TIFY 5B |
| Pav_sc0000675.1_g1140.1.mk | 19.4 | 1449.9 | -6.2 | 0.000 | BON1-associated protein 2-like |
| Pav_sc0000747.1_g030.1.mk | 6.1 | 579.6 | -6.6 | 0.000 | RPM1-interacting protein 4-like |
| Pav_sc0000852.1_g070.1.mk | 0.7 | 78.3 | -6.9 | 0.000 | pumilio homolog 12-like |

**Table S6** Highly induced and repressed unique transcripts in DS leaves

| **Gene ID** | **LS** | **LCK** | **log2FC** | **FDR** | **Putative function** |
| --- | --- | --- | --- | --- | --- |
| Pav_sc0000028.1_g650.1.mk | 3768.1 | 698.9 | 2.4 | 0.000 | probable protein phosphatase 2C 13 |
| Pav_sc0000051.1_g110.1.mk | 3371.1 | 786.3 | 2.1 | 0.001 | zinc finger protein constans like2 |
| Pav_sc0000598.1_g150.1.mk | 462.5 | 88.4 | 2.4 | 0.000 | zinc finger protein constans like2 |
| Pav_sc0000220.1_g180.1.mk | 90.6 | 19.0 | 2.3 | 0.008 | beta-glucosidase 40-like |
| Pav_sc0000216.1_g510.1.mk | 39.7 | 7.3 | 2.5 | 0.034 | ABC transporter G family member 5 |
| Pav_co4008389.1_g010.1.mk | 218.2 | 58.2 | 1.9 | 0.014 | protein LURP-one-related 11-like |
| Pav_sc0000037.1_g510.1.mk | 1085.7 | 378.9 | 1.5 | 0.046 | probable 2-aminoethanethiol dioxygenase |
| Pav_sc0000037.1_g560.1.mk | 372.4 | 45.0 | 3.0 | 0.007 | CBL-interacting protein kinase 5 |
| Pav_sc0000044.1_g810.1.mk | 2919.3 | 1005.2 | 1.5 | 0.030 | salt tolerance protein-like |
| Pav_sc0000061.1_g030.1.mk | 14429.0 | 3506.7 | 2.0 | 0.001 | haloacid dehalogenase-like hydrolase domain-containing protein 3 |
| Pav_sc0000093.1_g150.1.mk | 642.3 | 195.2 | 1.7 | 0.027 | organ-specific protein S2-like |
| Pav_sc0000130.1_g860.1.mk | 84.0 | 13.4 | 2.7 | 0.001 | endoglucanase 17 |
| Pav_sc0000220.1_g1270.1.mk | 3189.1 | 851.7 | 1.9 | 0.003 | cyclic dof factor 1-like |
| Pav_sc0000229.1_g490.1.mk | 420.5 | 138.9 | 1.6 | 0.027 | cytochrome P450 736A117 |
| Pav_sc0000348.1_g430.1.br | 21.1 | 0.3 | 6.0 | 0.027 | methylesterase 10 |
| Pav_sc0000354.1_g050.1.mk | 48.6 | 10.3 | 2.2 | 0.029 | WAT1-related protein At4g15540-like |
| Pav_sc0000396.1_g1160.1.mk | 1339.8 | 144.2 | 3.2 | 0.000 | light-inducible protein CPRF2 |
| Pav_sc0000474.1_g140.1.mk | 1681.5 | 425.1 | 2.0 | 0.002 | protein notum homolog isoform X2 |
| Pav_sc0000554.1_g150.1.mk | 50.1 | 5.7 | 3.1 | 0.001 | sericin-2 |
| Pav_sc0000557.1_g170.1.mk | 939.7 | 229.7 | 2.0 | 0.002 | ARGOS-like protein |
| Pav_sc0000580.1_g160.1.mk | 1293.8 | 143.7 | 3.2 | 0.000 | BAHD acyltransferase DCR |
| Pav_sc0000588.1_g480.1.mk | 27735.5 | 9638.9 | 1.5 | 0.040 | S-adenosylmethionine decarboxylase proenzyme |
| Pav_sc0000716.1_g230.1.mk | 2297.3 | 712.5 | 1.7 | 0.022 | auxin-responsive protein IAA1 |
| Pav_sc0000800.1_g710.1.mk | 35.8 | 5.9 | 2.6 | 0.022 | GATA transcription factor 12 |
| Pav_sc0000877.1_g870.1.mk | 2370.4 | 396.4 | 2.6 | 0.000 | cyclic dof factor 3 |
| Pav_sc0001046.1_g470.1.mk | 15220.4 | 4567.5 | 1.7 | 0.011 | protein LHY |
| Pav_sc0001090.1_g190.1.mk | 1834.5 | 577.4 | 1.7 | 0.026 | phytosulfokines-like |
| Pav_sc0001196.1_g1870.1.mk | 2729.4 | 856.5 | 1.7 | 0.018 | protein NRT1/ PTR FAMILY 4.3-like |
| Pav_sc0001289.1_g140.1.mk | 1775.2 | 582.5 | 1.6 | 0.027 | bidirectional sugar transporter SWEET2a-like |
| Pav_sc0001305.1_g350.1.br | 1339.0 | 388.3 | 1.8 | 0.008 | CBS domain-containing protein CBSX5-like isoform X1 |
| Pav_sc0001323.1_g1090.1.mk | 129.4 | 30.7 | 2.1 | 0.008 | peroxidase 43 |
| Pav_sc0001405.1_g250.1.mk | 1990.2 | 707.8 | 1.5 | 0.045 | gibberellin 20 oxidase 1-D |
| Pav_sc0001429.1_g160.1.br | 51.1 | 12.6 | 2.0 | 0.048 | BAHD acyltransferase At5g47980-like |
| Pav_sc0001762.1_g030.1.br | 295.4 | 88.8 | 1.7 | 0.019 | probable disease resistance protein At4g27220 |
| Pav_sc0001859.1_g200.1.mk | 2879.4 | 219.5 | 3.7 | 0.000 | lisH domain-containing protein C1711.05-like |
| Pav_sc0001883.1_g190.1.mk | 419.1 | 140.3 | 1.6 | 0.038 | two-component response regulator ARR9-like |
| Pav_sc0001885.1_g070.1.mk | 5939.4 | 1752.8 | 1.8 | 0.027 | conserved hypothetical protein |
| Pav_sc0002181.1_g020.1.mk | 44.0 | 8.0 | 2.5 | 0.021 | pleiotropic drug resistance protein 1-like |
| Pav_sc0002264.1_g050.1.br | 442.3 | 123.6 | 1.8 | 0.021 | receptor-like protein 2 |
| Pav_sc0002319.1_g030.1.mk | 2313.5 | 632.4 | 1.9 | 0.021 | two-component response regulator ARR8 |
| **Gene ID** | **RS** | **RCK** | **log2FC** | **FDR** | **Putative function** |
| Pav_sc0002358.1_g060.1.br | 239.0 | 63.6 | 1.9 | 0.020 | malonyl-CoA anthocyanidin 5-O-glucoside-6''-O-malonyltransferase-like |
| Pav_sc0002359.1_g160.1.mk | 2143.1 | 242.1 | 3.1 | 0.003 | inositol transporter 1 isoform X1 |
| Pav_sc0002716.1_g020.1.br | 193.4 | 43.2 | 2.2 | 0.005 | salicylate carboxymethyltransferase-like |
| Pav_sc0005572.1_g010.1.br | 692.6 | 240.7 | 1.5 | 0.047 | salicylate carboxymethyltransferase-like |
| Pav_sc0004467.1_g010.1.mk | 41.9 | 5.7 | 2.9 | 0.005 | DNA topoisomerase 1-like, partial |
| Pav_sc0007269.1_g010.1.mk | 75.1 | 11.7 | 2.7 | 0.049 | probable E3 ubiquitin-protein ligase BAH1-like 1 |
| Pav_sc0000051.1_g460.1.mk | 2230.9 | 353.1 | 2.7 | 0.001 | hypothetical protein JCGZ_25652 |
| Pav_sc0000909.1_g080.1.mk | 35.4 | 2.1 | 4.0 | 0.000 | hypothetical protein PRUPE_ppa008368mg |
| Pav_sc0001110.1_g040.1.mk | 299.6 | 90.9 | 1.7 | 0.019 | hypothetical protein PRUPE_ppa015982mg |
| Pav_sc0000094.1_g210.1.mk | 786.4 | 261.7 | 1.6 | 0.047 | hypothetical protein PRUPE_ppa005009mg |
| Pav_sc0000395.1_g200.1.mk | 3290.6 | 1059.6 | 1.6 | 0.027 | hypothetical protein PRUPE_ppa005330mg |
| Pav_sc0000164.1_g470.1.mk | 179.6 | 26.9 | 2.7 | 0.040 | hypothetical protein PRUPE_ppa003618mg |
| Pav_sc0007253.1_g040.1.mk | 973.7 | 97.9 | 3.3 | 0.008 | hypothetical protein PRUPE_ppa016167mg |
| Pav_sc0000563.1_g250.1.mk | 30.8 | 2.8 | 3.5 | 0.004 | hypothetical protein PRUPE_ppa021913mg |
| Novel00304 | 525.4 | 79.2 | 2.7 | 0.000 | hypothetical protein PRUPE_ppa022133mg |
| Pav_sc0000367.1_g200.1.mk | 3098.3 | 433.6 | 2.8 | 0.000 | uncharacterized protein LOC103321152 |
| Novel01519 | 144.0 | 46.3 | 1.6 | 0.050 | uncharacterized protein LOC103410364 |
| Pav_sc0001749.1_g180.1.mk | 203.0 | 59.5 | 1.8 | 0.034 | uncharacterized protein LOC103337641 |
| Pav_sc0002574.1_g130.1.mk | 380.0 | 80.7 | 2.2 | 0.026 | uncharacterized acetyltransferase At3g50280 |
| Pav_sc0002776.1_g020.1.mk | 84.7 | 11.4 | 2.9 | 0.034 | uncharacterized protein LOC103329348 |
| Pav_sc0003087.1_g030.1.br | 733.2 | 103.8 | 2.8 | 0.000 | uncharacterized protein LOC103929856 |
| Pav_sc0000078.1_g060.1.mk | 2381.0 | 620.7 | 1.9 | 0.002 | uncharacterized protein At1g01500 |
| Novel00194 | 31.7 | 1.9 | 4.1 | 0.001 | - |
| Novel00314 | 1076.7 | 123.7 | 3.1 | 0.000 | - |
| Novel00869 | 140.1 | 15.6 | 3.2 | 0.020 | - |
| Novel01239 | 100.8 | 17.3 | 2.5 | 0.001 | - |
| Novel01419 | 142.7 | 31.6 | 2.2 | 0.006 | - |
| Pav_sc0000048.1_g180.1.br | 17.3 | 0.9 | 4.2 | 0.018 | - |
| Pav_sc0000608.1_g070.1.mk | 79.2 | 14.2 | 2.5 | 0.003 | - |
| Pav_sc0000764.1_g270.1.br | 2856.9 | 973.7 | 1.6 | 0.045 | - |
| Pav_sc0000851.1_g070.1.br | 27.6 | 3.2 | 3.1 | 0.049 | - |
| Pav_sc0003492.1_g400.1.mk | 55.0 | 181.4 | -1.7 | 0.050 | probable pectinesterase/pectinesterase inhibitor 35 |
| Pav_co4031697.1_g010.1.mk | 172.2 | 1347.8 | -3.0 | 0.000 | 5'-adenylylsulfate reductase 3, chloroplastic-like |
| Pav_sc0000112.1_g190.1.mk | 88.5 | 310.9 | -1.8 | 0.026 | probable glutamate carboxypeptidase 2 |
| Pav_sc0000775.1_g210.1.mk | 51.6 | 209.4 | -2.0 | 0.020 | ATP sulfurylase 1, chloroplastic |
| Pav_sc0000196.1_g030.1.mk | 501.5 | 2502.2 | -2.3 | 0.000 | DEAD-box ATP-dependent RNA helicase 57 |
| Pav_sc0002106.1_g360.1.mk | 31.4 | 163.9 | -2.4 | 0.001 | DEAD-box ATP-dependent RNA helicase 28-like |
| Pav_sc0000775.1_g300.1.mk | 5937.7 | 25197.4 | -2.1 | 0.001 | NADPH quinone oxidoreductase-like |
| Pav_sc0000220.1_g2360.1.mk | 49.1 | 249.8 | -2.3 | 0.001 | 26S proteasome non-ATPase regulatory subunit 10 |
| Pav_sc0000349.1_g160.1.mk | 46.3 | 157.9 | -1.8 | 0.050 | putative pre-mRNA-splicing factor ATP-dependent RNA helicase PRP1 |
| Novel00640 | 8.2 | 66.4 | -3.0 | 0.001 | golgin subfamily A member 4 isoform X3 |
| Novel01227 | 376.5 | 1589.3 | -2.1 | 0.003 | filament-like plant protein plant protein |
| **Gene ID** | **RS** | **RCK** | **log2FC** | **FDR** | **Putative function** |
| Novel00066 | 269.8 | 930.0 | -1.8 | 0.013 | gag-pol polyprotein |
| Novel00202 | 7.0 | 107.9 | -3.9 | 0.050 | early nodulin-like protein |
| Pav_co3993429.1_g010.1.br | 22.8 | 114.5 | -2.3 | 0.004 | wall-associated receptor kinase 1-like |
| Pav_sc0000174.1_g1620.1.br | 40.2 | 205.9 | -2.4 | 0.001 | bidirectional sugar transporter SWEET17 |
| Novel01651 | 15.5 | 70.4 | -2.2 | 0.027 | putative disease resistance protein RGA3 |
| Novel01652 | 24.5 | 107.5 | -2.1 | 0.017 | putative disease resistance protein RGA3 |
| Pav_co4076473.1_g010.1.mk | 2.0 | 125.6 | -5.9 | 0.000 | zinc transporter 8-like |
| Pav_sc0000069.1_g800.1.mk | 1413.7 | 8876.7 | -2.7 | 0.002 | zinc transporter 4, chloroplastic |
| Pav_sc0000042.1_g360.1.mk | 75.0 | 325.5 | -2.1 | 0.005 | transcription factor bHLH137 isoform X2 |
| Pav_sc0000103.1_g720.1.mk | 115.7 | 414.3 | -1.8 | 0.021 | zinc finger protein VAR3, chloroplastic |
| Pav_sc0000102.1_g430.1.mk | 30.3 | 312.9 | -3.4 | 0.000 | bHLH33 |
| Pav_sc0000183.1_g470.1.mk | 1576.7 | 9433.3 | -2.6 | 0.000 | zinc transporter 8-like |
| Pav_sc0000143.1_g270.1.mk | 7.9 | 72.4 | -3.2 | 0.000 | RING-H2 finger protein ATL66 |
| Pav_sc0006018.1_g110.1.mk | 1.8 | 31.2 | -4.1 | 0.001 | transcription factor MYB86-like |
| Pav_sc0000373.1_g800.1.mk | 3.0 | 38.5 | -3.7 | 0.001 | zinc finger protein CONSTANS-LIKE 7 |
| Pav_sc0000308.1_g560.1.mk | 270.7 | 1102.4 | -2.0 | 0.004 | ethylene-responsive transcription factor RAP2-7 isoform X2 |
| Pav_sc0000373.1_g800.1.mk | 3.0 | 38.5 | -3.7 | 0.001 | zinc finger protein CONSTANS-LIKE 7 |
| Pav_sc0000491.1_g090.1.mk | 31.6 | 187.2 | -2.6 | 0.000 | zinc transporter 5 |
| Pav_sc0000749.1_g220.1.mk | 50.6 | 162.8 | -1.7 | 0.034 | transcription factor bHLH149 |
| Pav_sc0000800.1_g020.1.mk | 26.1 | 102.3 | -2.0 | 0.049 | transcription factor MYB44-like |
| Pav_sc0000907.1_g580.1.mk | 17.5 | 98.3 | -2.5 | 0.024 | F-box protein PP2-B15-like |
| Pav_sc0000909.1_g330.1.mk | 102.3 | 356.7 | -1.8 | 0.021 | F-box/kelch-repeat protein At2g44130-like |
| Pav_sc0001102.1_g1140.1.mk | 193.0 | 681.2 | -1.8 | 0.015 | AP2/EREBP family transcription factor-like protein 15 |
| Pav_sc0001331.1_g080.1.mk | 127.7 | 519.1 | -2.0 | 0.003 | zinc finger protein CONSTANS-LIKE 9-like |
| Pav_sc0001450.1_g070.1.mk | 3.7 | 33.1 | -3.2 | 0.007 | zinc finger BED domain-containing protein RICESLEEPER 2-like |
| Pav_sc0001518.1_g170.1.mk | 69.5 | 233.4 | -1.7 | 0.047 | NAC domain-containing protein 72 |
| Pav_sc0000023.1_g230.1.mk | 1.9 | 26.4 | -3.8 | 0.011 | serine carboxypeptidase-like 25 |
| Pav_sc0000069.1_g710.1.mk | 37.8 | 164.3 | -2.1 | 0.027 | probable leucine-rich repeat receptor-like protein kinase At1g68400 |
| Pav_sc0000071.1_g480.1.mk | 313.9 | 1321.5 | -2.1 | 0.002 | probable serine/threonine-protein kinase At1g01540 |
| Pav_sc0000176.1_g210.1.mk | 60.5 | 199.4 | -1.7 | 0.050 | aspartate, glycine, lysine and serine-rich protein-like |
| Pav_sc0000207.1_g1320.1.mk | 569.2 | 5730.1 | -3.3 | 0.000 | leucine-rich repeat extensin-like protein 3 |
| Pav_sc0000220.1_g2340.1.mk | 3.7 | 65.9 | -4.2 | 0.000 | LRR receptor-like serine/threonine-protein kinase |
| Pav_sc0000254.1_g1050.1.mk | 73.7 | 301.1 | -2.0 | 0.009 | GDSL esterase/lipase At4g01130 |
| Pav_sc0000257.1_g060.1.mk | 125.7 | 1104.4 | -3.1 | 0.000 | purine permease 3-like |
| Pav_sc0000373.1_g290.1.mk | 235.3 | 788.7 | -1.7 | 0.029 | serine/threonine protein phosphatase 2A 57 kDa regulatory subunit B' beta isoform-like |
| Pav_sc0000583.1_g270.1.mk | 5.0 | 388.9 | -6.3 | 0.000 | proline-rich receptor-like protein kinase PERK2 |
| Pav_sc0000661.1_g130.1.mk | 164.2 | 584.8 | -1.8 | 0.015 | proline-rich receptor-like protein kinase PERK2 isoform X2 |
| Pav_sc0000618.1_g360.1.mk | 73.6 | 317.6 | -2.1 | 0.004 | probable LRR receptor-like serine/threonine-protein kinase At3g47570 |
| Pav_sc0000996.1_g110.1.mk | 443.1 | 1930.8 | -2.1 | 0.003 | probable LRR receptor-like serine/threonine-protein kinase At1g56140 |
| Pav_sc0002024.1_g060.1.br | 16.6 | 139.7 | -3.1 | 0.000 | lysine-specific demethylase 8 |
| **Gene ID** | **RS** | **RCK** | **log2FC** | **FDR** | **Putative function** |
| Pav_sc0002479.1_g040.1.mk | 601.6 | 6071.9 | -3.3 | 0.000 | probable inactive leucine-rich repeat receptor-like protein kinase At3g03770 |
| Pav_sc0002862.1_g110.1.mk | 60.7 | 227.6 | -1.9 | 0.013 | probable LRR receptor-like serine/threonine-protein kinase At1g12460 |
| Pav_sc0003681.1_g080.1.mk | 75.9 | 367.0 | -2.3 | 0.001 | lysine histidine transporter-like 8 |
| Pav_sc0006018.1_g350.1.mk | 23.3 | 104.1 | -2.2 | 0.012 | serine carboxypeptidase-like 40 |
| Pav_sc0004305.1_g260.1.mk | 16.9 | 108.3 | -2.7 | 0.001 | arginine N-methyltransferase 2 |
| Pav_sc0000002.1_g060.1.mk | 121.9 | 481.1 | -2.0 | 0.008 | bifunctional 3-dehydroquinate dehydratase/shikimate dehydrogenase, chloroplastic-like isoform X1 |
| Pav_sc0000009.1_g080.1.mk | 148.0 | 509.7 | -1.8 | 0.024 | polyribonucleotide nucleotidyltransferase 2, mitochondrial |
| Pav_sc0000010.1_g080.1.mk | 1493.7 | 12062.6 | -3.0 | 0.000 | isoflavone reductase homolog |
| Pav_sc0000017.1_g1480.1.mk | 133.7 | 766.3 | -2.5 | 0.000 | probable pectinesterase/pectinesterase inhibitor 35 |
| Pav_sc0000062.1_g460.1.mk | 3549.5 | 13810.1 | -2.0 | 0.003 | alpha-glucan phosphorylase, H isozyme |
| Pav_sc0000067.1_g080.1.mk | 3.7 | 39.3 | -3.4 | 0.001 | Inactive protein kinase |
| Pav_sc0000067.1_g710.1.mk | 90.9 | 955.8 | -3.4 | 0.000 | trans-resveratrol di-O-methyltransferase-like |
| Pav_sc0000103.1_g660.1.mk | 101.3 | 335.5 | -1.7 | 0.033 | phosphoethanolamine N-methyltransferase 1 isoform X1 |
| Pav_sc0000100.1_g490.1.mk | 399.4 | 1263.1 | -1.7 | 0.028 | UDP-glucuronate 4-epimerase 6 |
| Pav_sc0000130.1_g210.1.br | 45.2 | 296.1 | -2.7 | 0.000 | probable galacturonosyltransferase-like 7 |
| Pav_sc0000259.1_g320.1.mk | 80.1 | 460.3 | -2.5 | 0.000 | bifunctional dethiobiotin synthetase/7,8-diamino-pelargonic acid aminotransferase, mitochondrial |
| Pav_sc0000375.1_g730.1.mk | 500.4 | 1782.6 | -1.8 | 0.012 | peptidyl-prolyl cis-trans isomerase CYP19-3 |
| Pav_sc0000467.1_g190.1.br | 4.2 | 39.3 | -3.2 | 0.003 | lysosomal Pro-X carboxypeptidase-like |
| Pav_sc0000467.1_g600.1.mk | 161.8 | 684.7 | -2.1 | 0.004 | lysosomal Pro-X carboxypeptidase-like |
| Pav_sc0000030.1_g040.1.mk | 35.5 | 336.2 | -3.2 | 0.000 | probable beta-D-xylosidase 7 |
| Pav_sc0000044.1_g1060.1.mk | 78.3 | 384.9 | -2.3 | 0.001 | beta-galactosidase 3 |
| Pav_sc0000554.1_g1670.1.mk | 43.8 | 423.1 | -3.3 | 0.000 | glutathione S-transferase DHAR2-like |
| Pav_sc0000554.1_g2030.1.mk | 667.8 | 2398.4 | -1.8 | 0.013 | DNA-directed RNA polymerase 3, chloroplastic |
| Pav_sc0000557.1_g650.1.mk | 429.5 | 1446.6 | -1.8 | 0.026 | putative quinone-oxidoreductase homolog, chloroplastic |
| Pav_sc0000558.1_g420.1.mk | 452.7 | 1529.9 | -1.8 | 0.013 | galacturonosyltransferase 8 |
| Pav_sc0000558.1_g750.1.mk | 379.6 | 1460.7 | -1.9 | 0.005 | alpha-xylosidase 1 |
| Pav_sc0000567.1_g1160.1.mk | 104.4 | 350.1 | -1.7 | 0.032 | probable galacturonosyltransferase-like 3 |
| Pav_sc0000661.1_g300.1.mk | 7.1 | 79.0 | -3.5 | 0.000 | probable trehalose-phosphate phosphatase D |
| Pav_sc0000691.1_g130.1.mk | 34.1 | 214.6 | -2.7 | 0.000 | UDP-glycosyltransferase 85A2-like |
| Pav_sc0000910.1_g200.1.mk | 1922.0 | 13172.8 | -2.8 | 0.000 | glucose-6-phosphate/phosphate translocator 2, chloroplastic |
| Pav_co4056075.1_g010.1.mk | 58.3 | 249.7 | -2.1 | 0.036 | 36.4 kDa proline-rich protein-like |
| Pav_sc0000009.1_g480.1.br | 57.5 | 776.9 | -3.8 | 0.000 | 2-methylene-furan-3-one reductase-like |
| Pav_sc0000009.1_g510.1.br | 271.5 | 2134.4 | -3.0 | 0.000 | 2-methylene-furan-3-one reductase-like |
| Pav_sc0000009.1_g900.1.mk | 16.4 | 94.8 | -2.5 | 0.003 | WAT1-related protein At5g40240-like |
| Pav_sc0000023.1_g690.1.mk | 1740.0 | 6907.7 | -2.0 | 0.001 | cyclic nucleotide-gated ion channel 2 |
| Pav_sc0000030.1_g200.1.mk | 2719.6 | 9134.8 | -1.7 | 0.017 | sulfate transporter 1.3-like |
| Pav_sc0000037.1_g170.1.mk | 207.4 | 1136.4 | -2.5 | 0.000 | protein trichome birefringence-like 37 |
| Pav_sc0000037.1_g200.1.mk | 6.5 | 61.3 | -3.2 | 0.003 | zingipain-2-like |
| Pav_sc0000040.1_g1070.1.mk | 798.2 | 2633.7 | -1.7 | 0.011 | cystinosin homolog isoform X1 |
| Pav_sc0000042.1_g280.1.mk | 6.6 | 41.3 | -2.6 | 0.022 | CASP-like protein POPTRDRAFT_823430 |
| **Gene ID** | **RS** | **RCK** | **log2FC** | **FDR** | **Putative function** |
| Pav_sc0000044.1_g560.1.mk | 144.8 | 1510.9 | -3.4 | 0.000 | snakin-2-like |
| Pav_sc0000045.1_g090.1.br | 4.8 | 58.3 | -3.6 | 0.012 | putative ribonuclease H protein At1g65750 |
| Pav_sc0000052.1_g450.1.br | 943.7 | 3181.1 | -1.8 | 0.007 | extensin-3-like |
| Pav_sc0000052.1_g680.1.mk | 35.6 | 273.1 | -2.9 | 0.000 | fasciclin-like arabinogalactan protein 1 |
| Pav_sc0000062.1_g160.1.mk | 2028.8 | 6777.2 | -1.7 | 0.018 | Tubulin beta-8 chain -like protein |
| Pav_sc0000069.1_g260.1.mk | 211.9 | 797.6 | -1.9 | 0.011 | solute carrier family 25 member 44-like |
| Pav_sc0000072.1_g210.1.mk | 140.2 | 483.9 | -1.8 | 0.017 | armadillo repeat-containing kinesin-like protein 2 isoform X1 |
| Pav_sc0000084.1_g450.1.mk | 184.2 | 1246.5 | -2.8 | 0.001 | GATA transcription factor 8 |
| Pav_sc0000087.1_g500.1.mk | 53.6 | 254.1 | -2.2 | 0.001 | epsin-3 |
| Pav_sc0000093.1_g640.1.mk | 35.1 | 206.5 | -2.6 | 0.001 | protein RADIALIS-like 3 |
| Pav_sc0000095.1_g1120.1.mk | 113.2 | 438.8 | -2.0 | 0.003 | cationic amino acid transporter 8, vacuolar |
| Pav_sc0000095.1_g960.1.mk | 263.0 | 812.6 | -1.6 | 0.049 | bystin |
| Pav_sc0000099.1_g590.1.mk | 302.3 | 960.3 | -1.7 | 0.022 | syntaxin-112-like |
| Pav_sc0000099.1_g740.1.mk | 143.9 | 545.8 | -1.9 | 0.011 | nucleolar protein 6 |
| Pav_sc0000108.1_g390.1.mk | 36.0 | 221.8 | -2.6 | 0.000 | UPF0481 protein At3g47200-like |
| Pav_sc0000119.1_g230.1.mk | 2199.5 | 6636.7 | -1.6 | 0.050 | extensin-like |
| Pav_sc0000129.1_g1200.1.mk | 15.7 | 182.1 | -3.5 | 0.000 | subtilisin-like protease |
| Pav_sc0000129.1_g180.1.mk | 6.9 | 52.0 | -2.9 | 0.003 | putative auxin-binding protein 2 |
| Pav_sc0000129.1_g880.1.mk | 47.8 | 917.1 | -4.3 | 0.000 | universal stress protein A-like protein |
| Pav_sc0000130.1_g490.1.mk | 140.5 | 543.0 | -2.0 | 0.006 | pentatricopeptide repeat-containing protein At3g56030 |
| Pav_sc0000143.1_g300.1.mk | 46.8 | 311.2 | -2.7 | 0.000 | adenylate isopentenyltransferase 3, chloroplastic |
| Pav_sc0000146.1_g150.1.mk | 12.8 | 130.5 | -3.4 | 0.000 | mRNA-decapping enzyme-like protein |
| Pav_sc0000159.1_g160.1.mk | 31.1 | 131.7 | -2.1 | 0.009 | repetitive proline-rich cell wall protein-like |
| Pav_sc0000166.1_g190.1.mk | 1292.0 | 6583.6 | -2.3 | 0.000 | pumilio homolog 24 |
| Pav_sc0000174.1_g1330.1.mk | 137.6 | 521.9 | -1.9 | 0.011 | major cherry allergen Pru av 1.0202 |
| Pav_sc0000174.1_g380.1.mk | 9.3 | 186.7 | -4.3 | 0.000 | cell division control protein 48 homolog B |
| Pav_sc0000203.1_g050.1.mk | 142.3 | 550.1 | -2.0 | 0.008 | non-specific lipid-transfer protein 1-like |
| Pav_sc0000206.1_g690.1.mk | 6.6 | 39.9 | -2.6 | 0.022 | protein sulfur dfficiency induced 2-like |
| Pav_sc0000220.1_g410.1.br | 0.9 | 19.3 | -4.5 | 0.005 | probable protein S-acyltransferase 22 |
| Pav_sc0000221.1_g250.1.mk | 794.0 | 2779.2 | -1.8 | 0.013 | expansin-like A2 |
| Pav_sc0000244.1_g040.1.mk | 120.1 | 784.3 | -2.7 | 0.010 | protein trichome birefringence-like 25 isoform X2 |
| Pav_sc0000257.1_g550.1.mk | 478.4 | 1590.8 | -1.7 | 0.017 | protein NRT1/ PTR FAMILY 4.6 |
| Pav_sc0000259.1_g290.1.mk | 19.7 | 134.9 | -2.8 | 0.017 | Momilactone A synthase |
| Pav_sc0000326.1_g590.1.br | 57.7 | 255.8 | -2.1 | 0.029 | probable vacuolar amino acid transporter YPQ1 |
| Pav_sc0000348.1_g1030.1.mk | 146.0 | 1816.6 | -3.6 | 0.000 | subtilisin-like protease |
| Pav_sc0000351.1_g300.1.mk | 72.8 | 248.9 | -1.8 | 0.037 | BAG family molecular chaperone regulator 1-like |
| Pav_sc0000355.1_g300.1.mk | 702.8 | 2052.0 | -1.5 | 0.036 | protein SPIRAL1-like 3 |
| Pav_sc0000358.1_g370.1.mk | 1407.2 | 4932.8 | -1.8 | 0.011 | cleavage stimulation factor subunit 1 isoform X2 |
| Pav_sc0000358.1_g570.1.mk | 73.8 | 259.2 | -1.8 | 0.033 | monocopper oxidase-like protein SKU5 |
| Pav_sc0000370.1_g260.1.mk | 128.2 | 451.1 | -1.8 | 0.022 | subtilisin-like protease |
| Pav_sc0000396.1_g670.1.mk | 151.5 | 788.2 | -2.4 | 0.000 | UPF0481 protein At3g47200-like |
| Pav_sc0000405.1_g550.1.mk | 8.1 | 41.8 | -2.4 | 0.046 | protein EXORDIUM-like 5 |
| Pav_sc0000412.1_g480.1.mk | 202.9 | 4925.9 | -4.6 | 0.000 | copper transport protein ATX1 |
| **Gene ID** | **RS** | **RCK** | **log2FC** | **FDR** | **Putative function** |
| Pav_sc0000481.1_g130.1.mk | 84.5 | 330.6 | -2.0 | 0.008 | pentatricopeptide repeat-containing protein At1g02370, mitochondrial |
| Pav_sc0000499.1_g090.1.mk | 24.9 | 100.7 | -2.0 | 0.023 | H/ACA ribonucleoprotein complex subunit 4-like |
| Pav_sc0000540.1_g200.1.mk | 572.5 | 1803.5 | -1.7 | 0.044 | ammonium transporter 3 member 1-like |
| Pav_sc0000549.1_g660.1.br | 118.8 | 819.8 | -2.8 | 0.000 | aquaporin TIP1-3 |
| Pav_sc0000567.1_g700.1.mk | 46.8 | 484.6 | -3.4 | 0.000 | 1-aminocyclopropane-1-carboxylate oxidase homolog 11-like |
| Pav_sc0000586.1_g740.1.mk | 65.2 | 498.6 | -2.9 | 0.000 | DNA-directed RNA polymerase 1, mitochondrial |
| Pav_sc0000591.1_g200.1.mk | 1407.5 | 4470.3 | -1.7 | 0.022 | cellulose synthase-like protein D3 |
| Pav_sc0000648.1_g210.1.mk | 157.7 | 730.4 | -2.2 | 0.001 | protein yippee-like At4g27745 |
| Pav_sc0000659.1_g360.1.mk | 36.6 | 131.7 | -1.8 | 0.040 | DNA-directed RNA polymerase 2, chloroplastic/mitochondrial isoform X1 |
| Pav_sc0000697.1_g090.1.mk | 49.8 | 185.9 | -1.9 | 0.029 | tRNA (guanine(10)-N2)-methyltransferase homolog |
| Pav_sc0000700.1_g600.1.mk | 66.1 | 303.8 | -2.2 | 0.002 | probable inactive receptor kinase At2g26730 |
| Pav_sc0000704.1_g050.1.mk | 56.5 | 223.0 | -2.0 | 0.012 | pentatricopeptide repeat-containing protein At1g31430-like |
| Pav_sc0000704.1_g160.1.mk | 332.1 | 1947.4 | -2.6 | 0.002 | protein FAF-like, chloroplastic |
| Pav_sc0000713.1_g260.1.mk | 152.4 | 1113.6 | -2.9 | 0.000 | root phototropism protein 3-like |
| Pav_sc0000716.1_g530.1.mk | 31.2 | 257.4 | -3.0 | 0.000 | organic cation/carnitine transporter 3-like |
| Pav_sc0000736.1_g070.1.mk | 75.7 | 467.1 | -2.6 | 0.000 | nucleotide pyrophosphatase/phosphodiesterase-like |
| Pav_sc0000740.1_g610.1.mk | 5.1 | 42.0 | -3.0 | 0.005 | elongation factor 1-alpha-like |
| Pav_sc0000764.1_g130.1.mk | 84.1 | 365.2 | -2.1 | 0.001 | calmodulin-like protein 11 isoform X2 |
| Pav_sc0000800.1_g060.1.mk | 5.1 | 89.1 | -4.1 | 0.000 | class 3 truncated hemoglobin |
| Pav_sc0000800.1_g1370.1.mk | 206.5 | 1860.1 | -3.2 | 0.000 | cation transport regulator-like protein 2 |
| Pav_sc0000800.1_g1580.1.mk | 1063.7 | 17763.1 | -4.1 | 0.000 | histidine-containing phosphotransfer protein 4-like |
| Pav_sc0000800.1_g510.1.mk | 4.7 | 41.1 | -3.1 | 0.017 | chitin elicitor receptor kinase 1-like |
| Pav_sc0000843.1_g580.1.mk | 394.4 | 1393.2 | -1.8 | 0.016 | monothiol glutaredoxin-S6-like |
| Pav_sc0000848.1_g520.1.mk | 559.2 | 2750.9 | -2.3 | 0.000 | pheromone-processing carboxypeptidase KEX1-like |
| Pav_sc0000848.1_g760.1.mk | 132.5 | 845.9 | -2.7 | 0.000 | probable inactive receptor kinase At5g67200 |
| Pav_sc0000852.1_g470.1.mk | 22.4 | 99.4 | -2.1 | 0.013 | chromosome-associated kinesin KIF4A-like |
| Pav_sc0000852.1_g490.1.mk | 264.3 | 819.0 | -1.6 | 0.049 | abscisic acid 8'-hydroxylase 4-like |
| Pav_sc0000852.1_g900.1.mk | 20.8 | 314.2 | -3.9 | 0.001 | 5'-adenylylsulfate reductase 3, chloroplastic-like |
| Pav_sc0000877.1_g1350.1.mk | 4.2 | 63.4 | -3.9 | 0.000 | Beta-1,3-glucosyltransferase |
| Pav_sc0000886.1_g440.1.mk | 23.3 | 104.7 | -2.2 | 0.006 | protein PPLZ02 |
| Pav_sc0000890.1_g720.1.mk | 105.4 | 375.0 | -1.8 | 0.017 | pentatricopeptide repeat-containing protein At2g01740 |
| Pav_sc0000893.1_g400.1.mk | 18.9 | 170.6 | -3.2 | 0.000 | 65-kDa microtubule-associated protein 6-like |
| Pav_sc0000907.1_g880.1.mk | 467.1 | 16013.9 | -5.1 | 0.000 | feruloyl CoA ortho-hydroxylase 2-like |
| Pav_sc0000908.1_g280.1.mk | 121.1 | 666.2 | -2.5 | 0.000 | NO-associated protein 1, chloroplastic/mitochondrial |
| Pav_sc0000909.1_g710.1.mk | 192.2 | 1379.9 | -2.8 | 0.000 | 5'-adenylylsulfate reductase 3, chloroplastic-like |
| Pav_sc0000910.1_g690.1.mk | 83.6 | 299.4 | -1.8 | 0.013 | probable xyloglucan endotransglucosylase/hydrolase protein 8 |
| Pav_sc0000910.1_g790.1.mk | 5.1 | 80.8 | -4.0 | 0.015 | aldo-keto reductase family 4 member C9-like |
| Pav_sc0000955.1_g080.1.mk | 60.1 | 217.8 | -1.9 | 0.025 | putative alpha-xylosidase 2 |
| Pav_sc0001015.1_g180.1.mk | 34.2 | 144.0 | -2.1 | 0.006 | 14 kDa proline-rich protein DC2.15-like |
| Pav_sc0001040.1_g280.1.mk | 179.4 | 703.9 | -2.0 | 0.008 | homocysteine S-methyltransferase 1 |
| Pav_sc0001051.1_g010.1.mk | 96.8 | 843.8 | -3.1 | 0.000 | 5'-adenylylsulfate reductase 1, chloroplastic-like |
| Pav_sc0001102.1_g1030.1.mk | 316.5 | 2065.2 | -2.7 | 0.000 | somatic embryogenesis receptor kinase 4 |
| **Gene ID** | **RS** | **RCK** | **log2FC** | **FDR** | **Putative function** |
| Pav_sc0001102.1_g1090.1.mk | 3.2 | 48.6 | -3.9 | 0.000 | peptidyl-prolyl cis-trans isomerase FKBP53 isoform X1 |
| Pav_sc0001102.1_g230.1.mk | 778.6 | 2864.9 | -1.9 | 0.003 | monocopper oxidase-like protein SKS1 |
| Pav_sc0001102.1_g800.1.mk | 64.7 | 210.0 | -1.7 | 0.027 | SAUR family protein, putative |
| Pav_sc0001105.1_g280.1.mk | 117.5 | 395.5 | -1.8 | 0.035 | adenine phosphoribosyltransferase 1-like |
| Pav_sc0001125.1_g010.1.mk | 157.3 | 749.8 | -2.3 | 0.001 | TPR repeat-containing thioredoxin TTL1 |
| Pav_sc0001181.1_g030.1.mk | 639.6 | 2151.7 | -1.8 | 0.021 | probable pectate lyase 5 |
| Pav_sc0001183.1_g360.1.mk | 33.0 | 155.4 | -2.2 | 0.004 | feruloyl CoA ortho-hydroxylase 2-like |
| Pav_sc0001217.1_g030.1.mk | 191.5 | 1391.5 | -2.9 | 0.000 | WAT1-related protein At1g09380 |
| Pav_sc0001236.1_g400.1.mk | 32.3 | 250.6 | -3.0 | 0.000 | probable 2-oxoglutarate-dependent dioxygenase AOP1 |
| Pav_sc0001258.1_g090.1.mk | 34.6 | 133.8 | -2.0 | 0.030 | monosaccharide-sensing protein 2 |
| Pav_sc0001289.1_g570.1.mk | 10182.0 | 35970.1 | -1.8 | 0.015 | tetraspanin-19-like |
| Pav_sc0001305.1_g390.1.mk | 199.1 | 766.9 | -1.9 | 0.008 | receptor-like protein kinase At3g21340 |
| Pav_sc0001305.1_g980.1.mk | 65.5 | 309.6 | -2.2 | 0.001 | GDSL esterase/lipase At5g62930 |
| Pav_sc0001309.1_g1070.1.mk | 323.9 | 1350.6 | -2.1 | 0.002 | aluminum-activated malate transporter 2-like |
| Pav_sc0001335.1_g200.1.mk | 894.5 | 2709.5 | -1.6 | 0.038 | probable pectinesterase/pectinesterase inhibitor 34 |
| Pav_sc0001335.1_g240.1.mk | 120.9 | 633.8 | -2.4 | 0.000 | two-component response regulator-like APRR7 |
| Pav_sc0001345.1_g100.1.mk | 405.6 | 1582.2 | -2.0 | 0.006 | 60S ribosomal protein L21-1 |
| **Pav_sc0001472.1_g050.1.mk** | **19.6** | **133.3** | **-2.8** | **0.000** | **cationic peroxidase 1-like** |
| Pav_sc0001513.1_g190.1.mk | 69.9 | 418.1 | -2.6 | 0.000 | bifunctional phosphatase IMPL2, chloroplastic |
| Pav_sc0001554.1_g180.1.mk | 280.2 | 842.1 | -1.6 | 0.050 | cytochrome P450 714A1-like |
| Pav_sc0001582.1_g050.1.mk | 119.2 | 448.0 | -1.9 | 0.009 | probable methyltransferase PMT24 |
| Pav_sc0001601.1_g060.1.mk | 542.3 | 2935.5 | -2.4 | 0.000 | cytochrome P450 714A1-like |
| Pav_sc0001699.1_g700.1.mk | 84.9 | 257.3 | -1.6 | 0.042 | protein MIZU-KUSSEI 1 |
| Pav_sc0001794.1_g390.1.mk | 144.6 | 1670.6 | -3.5 | 0.000 | WAT1-related protein At1g70260 |
| Pav_sc0001827.1_g090.1.mk | 174.0 | 669.5 | -1.9 | 0.006 | cyclic nucleotide-gated ion channel 4 |
| Pav_sc0001846.1_g020.1.mk | 910.0 | 2767.2 | -1.6 | 0.016 | extensin-2-like |
| Pav_sc0001882.1_g070.1.mk | 4768.9 | 22488.0 | -2.2 | 0.000 | sulfur difficiency induced protein |
| Pav_sc0001963.1_g250.1.mk | 70.3 | 2610.6 | -5.2 | 0.000 | transcriptional corepressor LEUNIG-like isoform X1 |
| Pav_sc0001963.1_g370.1.mk | 410.2 | 1223.5 | -1.6 | 0.040 | cellulose synthase A catalytic subunit 2 |
| Pav_sc0002004.1_g150.1.mk | 1214.4 | 3855.2 | -1.7 | 0.018 | G-type lectin S-receptor-like serine/threonine-protein kinase RLK1 |
| Pav_sc0002024.1_g120.1.mk | 84.5 | 384.4 | -2.2 | 0.001 | probable receptor protein kinase TMK1 |
| Pav_sc0002055.1_g080.1.mk | 70.3 | 384.5 | -2.5 | 0.000 | endoglucanase 10-like |
| Pav_sc0002154.1_g100.1.mk | 312.7 | 975.3 | -1.6 | 0.044 | probable importin-7 homolog |
| Pav_sc0002179.1_g160.1.mk | 5.8 | 58.9 | -3.4 | 0.000 | glucan endo-1,3-beta-glucosidase 12 |
| Pav_sc0002206.1_g190.1.mk | 50.8 | 677.9 | -3.7 | 0.000 | protein ATAF2-like |
| Pav_sc0002207.1_g650.1.mk | 3.1 | 30.6 | -3.3 | 0.010 | probable GPI-anchored adhesin-like protein PGA55 |
| Pav_sc0002234.1_g110.1.mk | 78.2 | 303.3 | -2.0 | 0.010 | protein ABIL2-like |
| Pav_sc0002327.1_g1050.1.mk | 60.1 | 412.7 | -2.8 | 0.000 | oligopeptide transporter 2-like |
| Pav_sc0002327.1_g380.1.mk | 5.8 | 177.5 | -4.9 | 0.003 | G-type lectin S-receptor-like serine/threonine-protein kinase RLK1 |
| Pav_sc0002360.1_g830.1.mk | 38.4 | 200.7 | -2.4 | 0.044 | L-type lectin-domain containing receptor kinase IX.1-like |
| Pav_sc0002383.1_g160.1.br | 36.2 | 146.2 | -2.0 | 0.019 | L-type lectin-domain containing receptor kinase IX.1-like |
| Pav_sc0002383.1_g170.1.br | 18.7 | 83.4 | -2.2 | 0.028 | cyclin-dependent kinase inhibitor 7-like isoform X1 |
| Pav_sc0002451.1_g320.1.mk | 19.5 | 91.1 | -2.2 | 0.013 | chlorophyll a-b binding protein 6, chloroplastic |
| **Gene ID** | **RS** | **RCK** | **log2FC** | **FDR** | **Putative function** |
| Pav_sc0002681.1_g030.1.mk | 17.2 | 78.4 | -2.2 | 0.019 | protein ROOT PRIMORDIUM DEFECTIVE 1 |
| Pav_sc0002772.1_g120.1.mk | 17.4 | 112.2 | -2.7 | 0.001 | adenylyl-sulfate kinase 3 |
| Pav_sc0002842.1_g010.1.mk | 4.1 | 47.8 | -3.5 | 0.000 | alcohol dehydrogenase |
| Pav_sc0002842.1_g220.1.mk | 57.1 | 189.4 | -1.7 | 0.040 | alcohol dehydrogenase 1 |
| Pav_sc0002858.1_g140.1.mk | 1.7 | 18.4 | -3.5 | 0.044 | tropinone reductase homolog At1g07440-like |
| Pav_sc0002858.1_g210.1.br | 111.5 | 355.4 | -1.7 | 0.043 | tropinone reductase homolog At1g07440-like |
| Pav_sc0002858.1_g270.1.br | 725.3 | 2591.9 | -1.8 | 0.009 | tropinone reductase homolog At1g07440-like |
| Pav_sc0002858.1_g290.1.br | 297.8 | 908.5 | -1.6 | 0.043 | probable sugar phosphate/phosphate translocator At1g12500 |
| Pav_sc0002862.1_g340.1.mk | 83.5 | 283.1 | -1.8 | 0.022 | L-type lectin-domain containing receptor kinase IX.1-like |
| Pav_sc0002962.1_g080.1.mk | 44.0 | 165.4 | -1.9 | 0.030 | probable mannitol dehydrogenase |
| Pav_sc0003583.1_g040.1.mk | 7.1 | 70.1 | -3.3 | 0.000 | shikimate O-hydroxycinnamoyltransferase-like |
| Pav_sc0003766.1_g050.1.mk | 60.3 | 283.0 | -2.2 | 0.001 | probable galacturonosyltransferase 15 |
| Pav_sc0003835.1_g230.1.mk | 509.5 | 1639.0 | -1.7 | 0.027 | brassinosteroid-regulated protein BRU1-like |
| Pav_sc0003915.1_g020.1.mk | 76.4 | 1398.1 | -4.2 | 0.003 | probable mannitol dehydrogenase |
| Pav_sc0003921.1_g050.1.mk | 657.6 | 2372.9 | -1.9 | 0.008 | probable protein arginine N-methyltransferase 3 |
| Pav_sc0004314.1_g050.1.mk | 76.7 | 278.8 | -1.9 | 0.020 | triacylglycerol lipase 2-like |
| Pav_sc0004913.1_g210.1.mk | 95.4 | 326.8 | -1.8 | 0.040 | 60S ribosome subunit biogenesis protein NIP7 homolog |
| Pav_sc0005273.1_g090.1.mk | 10.7 | 57.2 | -2.4 | 0.019 | pentatricopeptide repeat-containing protein At1g06710, mitochondrial |
| Pav_sc0005373.1_g060.1.mk | 80.6 | 269.3 | -1.7 | 0.044 | protein EXORDIUM-like 5 |
| Pav_sc0005640.1_g020.1.mk | 7.6 | 181.1 | -4.6 | 0.000 | pentatricopeptide repeat-containing protein At3g46790, chloroplastic |
| Pav_sc0005704.1_g010.1.mk | 29.5 | 108.9 | -1.9 | 0.046 | expansin |
| Pav_sc0006402.1_g100.1.mk | 274.5 | 815.5 | -1.6 | 0.049 | phospho-2-dehydro-3-deoxyheptonate aldolase 2, chloroplastic-like |
| Pav_sc0006673.1_g020.1.mk | 275.5 | 982.7 | -1.8 | 0.016 | prolyl endopeptidase-like |
| Pav_sc0007796.1_g070.1.mk | 1619.5 | 21314.5 | -3.7 | 0.000 | magnesium transporter MRS2-I-like |
| Pav_sc0010540.1_g020.1.mk | 332.2 | 1754.4 | -2.4 | 0.000 | cytochrome P450 81D11 |
| Pav_sc0011125.1_g010.1.br | 225.7 | 713.0 | -1.7 | 0.048 | putative disease resistance protein RGA3, partial |
| Pav_sc0011228.1_g020.1.br | 56.3 | 198.0 | -1.8 | 0.040 | probable beta-D-xylosidase 7 |
| Novel00036 | 13.3 | 94.3 | -2.8 | 0.001 | hypothetical protein PRUPE_ppa001023mg |
| Novel00571 | 37.3 | 175.1 | -2.2 | 0.017 | hypothetical protein PRUPE_ppa001116mg |
| Novel00072 | 97.1 | 533.5 | -2.5 | 0.000 | hypothetical protein PRUPE_ppa005107mg |
| Novel01170 | 1132.0 | 4486.1 | -2.0 | 0.003 | hypothetical protein PRUPE_ppa001453mg PRUPE_ppa001453mg |
| Novel00244 | 77.9 | 319.1 | -2.0 | 0.005 | hypothetical protein PRUPE_ppa024411mg |
| Novel01064 | 4.7 | 27.8 | -2.6 | 0.037 | hypothetical protein PRUPE_ppa023179mg |
| Novel01283 | 118.6 | 518.6 | -2.1 | 0.001 | hypothetical protein PRUPE_ppa004852mg |
| Novel01648 | 24.3 | 580.1 | -4.6 | 0.000 | hypothetical protein PRUPE_ppa022668mg |
| Pav_sc0000108.1_g470.1.mk | 128.7 | 371.3 | -1.5 | 0.041 | hypothetical protein PRUPE_ppa012437mg |
| Pav_sc0000072.1_g090.1.mk | 2.2 | 28.7 | -3.7 | 0.003 | hypothetical protein PRUPE_ppa026236mg |
| Pav_sc0000212.1_g080.1.br | 554.4 | 4433.4 | -3.0 | 0.000 | hypothetical protein L484_027250 |
| Pav_sc0000212.1_g1520.1.mk | 88.9 | 335.1 | -1.9 | 0.013 | hypothetical protein L484_021105 |
| Pav_sc0000220.1_g2330.1.mk | 30.6 | 540.0 | -4.1 | 0.000 | hypothetical protein B456_009G323500 |
| Pav_sc0000220.1_g400.1.mk | 514.8 | 7351.9 | -3.8 | 0.000 | hypothetical protein |
| Pav_sc0000220.1_g630.1.mk | 207.1 | 668.8 | -1.7 | 0.026 | hypothetical protein PRUPE_ppa002940mg |
| Pav_sc0000257.1_g050.1.mk | 60.4 | 236.7 | -2.0 | 0.012 | hypothetical protein PRUPE_ppa008424mg |
| **Gene ID** | **RS** | **RCK** | **log2FC** | **FDR** | **Putative function** |
| Pav_sc0000624.1_g1910.1.br | 1.1 | 25.7 | -4.6 | 0.001 | hypothetical protein PRUPE_ppa017508mg |
| Pav_sc0000638.1_g1130.1.br | 43.1 | 154.3 | -1.8 | 0.025 | hypothetical protein PRUPE_ppa011328mg |
| Pav_sc0000600.1_g150.1.mk | 163.3 | 643.3 | -2.0 | 0.002 | hypothetical protein PRUPE_ppa026352mg |
| Pav_sc0003756.1_g060.1.mk | 8.4 | 82.4 | -3.3 | 0.001 | hypothetical protein L484_003734 |
| Pav_sc0009726.1_g010.1.mk | 60.7 | 232.1 | -1.9 | 0.017 | hypothetical protein PRUPE_ppa001023mg |
| Pav_sc0000401.1_g160.1.mk | 3.1 | 29.9 | -3.3 | 0.012 | hypothetical protein PRUPE_ppa014957mg |
| Pav_sc0000691.1_g800.1.br | 40.3 | 153.5 | -1.9 | 0.035 | hypothetical protein L484_023803 |
| Pav_sc0000546.1_g020.1.mk | 55.4 | 215.0 | -2.0 | 0.021 | hypothetical protein PRUPE_ppb010066mg |
| Pav_sc0003503.1_g060.1.mk | 25.8 | 141.9 | -2.5 | 0.002 | hypothetical protein PRUPE_ppa014686mg, partial |
| Pav_sc0003583.1_g020.1.br | 3.1 | 114.9 | -5.2 | 0.000 | hypothetical protein PRUPE_ppa020116mg, partial |
| Pav_sc0000848.1_g750.1.mk | 84.7 | 370.0 | -2.1 | 0.003 | hypothetical protein L484_009082 |
| Pav_sc0001265.1_g080.1.mk | 312.9 | 1249.1 | -2.0 | 0.004 | hypothetical protein CICLE_v10016074mg |
| Pav_sc0001080.1_g1300.1.mk | 61.6 | 403.9 | -2.7 | 0.000 | hypothetical protein PRUPE_ppa009086mg |
| Pav_sc0001289.1_g560.1.mk | 11175.5 | 40907.8 | -1.9 | 0.011 | hypothetical protein PHAVU_002G196300g |
| Pav_sc0002342.1_g020.1.br | 27.8 | 139.5 | -2.3 | 0.002 | hypothetical protein L484_022196 |
| Pav_sc0002475.1_g240.1.mk | 7008.7 | 21759.3 | -1.6 | 0.044 | hypothetical protein POPTR_0008s17960g |
| Pav_sc0000467.1_g630.1.mk | 174.5 | 1402.2 | -3.0 | 0.000 | unnamed protein product |
| Pav_sc0000588.1_g070.1.mk | 259.0 | 931.5 | -1.8 | 0.012 | uncharacterized protein LOC103332245 isoform X2 |
| Pav_sc0000617.1_g430.1.mk | 123.3 | 405.7 | -1.7 | 0.041 | uncharacterized protein LOC103329666 |
| Pav_sc0000652.1_g590.1.br | 29.0 | 212.5 | -2.9 | 0.000 | uncharacterized protein LOC101312235 isoform X1 |
| Pav_sc0000793.1_g100.1.mk | 140.0 | 577.7 | -2.0 | 0.001 | uncharacterized protein LOC103320283 isoform X2 |
| Pav_sc0003079.1_g020.1.mk | 381.1 | 1339.4 | -1.8 | 0.011 | uncharacterized protein LOC103326011 |
| Pav_sc0003492.1_g280.1.mk | 411.6 | 1304.9 | -1.7 | 0.036 | uncharacterized protein LOC103326043 |
| Pav_sc0000824.1_g060.1.mk | 565.9 | 2586.5 | -2.2 | 0.001 | uncharacterized protein LOC103324435 |
| Pav_sc0000824.1_g070.1.mk | 182.9 | 909.2 | -2.3 | 0.001 | uncharacterized protein LOC103327533 |
| Pav_sc0000893.1_g880.1.mk | 856.9 | 2585.4 | -1.6 | 0.034 | uncharacterized protein DDB_G0271670 |
| Pav_sc0001031.1_g160.1.br | 14.6 | 66.9 | -2.2 | 0.027 | uncharacterized protein At1g04910-like |
| Pav_sc0001040.1_g260.1.mk | 996.1 | 3246.9 | -1.7 | 0.033 | uncharacterized protein At1g04910-like |
| Pav_sc0001077.1_g150.1.mk | 208.1 | 1850.1 | -3.2 | 0.000 | uncharacterized protein LOC103330360 |
| Pav_sc0001084.1_g290.1.mk | 42.9 | 7634.1 | -7.5 | 0.000 | uncharacterized protein LOC103333330 |
| Pav_sc0001163.1_g090.1.mk | 224.1 | 878.9 | -2.0 | 0.002 | uncharacterized protein LOC103341519 |
| Pav_sc0002080.1_g080.1.mk | 31.3 | 167.2 | -2.4 | 0.001 | uncharacterized protein LOC103323159 |
| Pav_sc0000872.1_g110.1.mk | 154.0 | 995.5 | -2.7 | 0.000 | uncharacterized protein At5g01610-like |
| Pav_sc0002214.1_g010.1.mk | 38.5 | 152.0 | -2.0 | 0.021 | uncharacterized protein LOC103335358 |
| Pav_sc0001502.1_g370.1.mk | 65.0 | 255.6 | -2.0 | 0.012 | uncharacterized protein At3g28850 |
| Pav_sc0002250.1_g080.1.mk | 172.7 | 915.7 | -2.4 | 0.000 | uncharacterized protein LOC103319390 |
| Pav_sc0002360.1_g680.1.mk | 1003.1 | 2856.6 | -1.5 | 0.036 | uncharacterized GPI-anchored protein At4g28100 |
| Pav_sc0002839.1_g450.1.mk | 172.8 | 792.2 | -2.2 | 0.001 | uncharacterized protein LOC103336415 |
| Pav_sc0003946.1_g060.1.mk | 95.8 | 415.4 | -2.1 | 0.003 | uncharacterized protein At1g04910-like |
| Pav_sc0004199.1_g010.1.br | 173.1 | 547.2 | -1.7 | 0.047 | unnamed protein product |
| Pav_sc0005746.1_g060.1.mk | 344.3 | 1356.7 | -2.0 | 0.050 | uncharacterized protein LOC103337946 |
| Pav_sc0000030.1_g1220.1.mk | 38.5 | 398.7 | -3.4 | 0.000 | uncharacterized protein At1g04910 |
| Novel00403 | 1.2 | 17.7 | -3.8 | 0.034 | uncharacterized protein LOC103340828 isoform X2 |
| **Gene ID** | **RS** | **RCK** | **log2FC** | **FDR** | **Putative function** |
| Novel00645 | 9.6 | 96.7 | -3.3 | 0.000 | uncharacterized protein LOC103340828 isoform X2 |
| Pav_sc0000040.1_g020.1.mk | 462.6 | 1785.5 | -1.9 | 0.003 | uncharacterized protein LOC103329820 |
| Pav_sc0000071.1_g620.1.mk | 92.8 | 600.8 | -2.7 | 0.000 | uncharacterized protein LOC103339889 |
| Pav_sc0000113.1_g140.1.mk | 1316.2 | 4145.7 | -1.7 | 0.030 | uncharacterized protein LOC103340317 |
| Novel00711 | 2.5 | 27.1 | -3.4 | 0.010 | - |
| Novel00762 | 206.2 | 665.6 | -1.7 | 0.032 | - |
| Novel00991 | 28.1 | 307.9 | -3.5 | 0.004 | - |
| Pav_sc0001015.1_g270.1.br | 102.1 | 760.1 | -2.9 | 0.000 | - |
| Novel01102 | 1.3 | 20.2 | -4.0 | 0.007 | - |
| Pav_sc0003583.1_g030.1.br | 2.8 | 95.2 | -5.1 | 0.000 | - |
| Pav_co4040199.1_g010.1.br | 9.7 | 49.2 | -2.3 | 0.033 | - |
| Novel01362 | 123.8 | 4025.1 | -5.0 | 0.000 | - |
| Novel01530 | 5.6 | 37.1 | -2.7 | 0.026 | - |
| Pav_co4070623.1_g010.1.mk | 10.7 | 74.9 | -2.8 | 0.021 | - |
| Pav_sc0000069.1_g750.1.mk | 13.5 | 887.3 | -6.0 | 0.000 | - |
| Pav_sc0000130.1_g200.1.mk | 1962.0 | 20338.8 | -3.4 | 0.000 | - |
| Pav_sc0000206.1_g670.1.mk | 384.0 | 2625.3 | -2.8 | 0.000 | - |
| Pav_sc0000323.1_g400.1.mk | 202.8 | 853.7 | -2.1 | 0.002 | - |
| Pav_sc0000254.1_g340.1.mk | 11.2 | 133.8 | -3.6 | 0.000 | - |
| Pav_sc0000497.1_g190.1.mk | 4.8 | 494.1 | -6.7 | 0.000 | - |
| Pav_sc0000430.1_g250.1.mk | 55.9 | 230.7 | -2.0 | 0.012 | - |
| Pav_sc0000652.1_g340.1.br | 43.8 | 151.2 | -1.8 | 0.036 | - |
| Pav_sc0000652.1_g580.1.br | 23.4 | 190.0 | -3.0 | 0.000 | - |
| Pav_sc0000704.1_g150.1.mk | 1.6 | 21.2 | -3.8 | 0.018 | - |
| Pav_sc0001339.1_g070.1.mk | 4824.1 | 13963.1 | -1.5 | 0.049 | - |
| Pav_sc0002842.1_g230.1.mk | 1212.1 | 6492.0 | -2.4 | 0.000 | - |
| Pav_sc0012345.1_g010.1.br | 5.3 | 37.8 | -2.8 | 0.017 | - |
| Pav_sc0009852.1_g010.1.mk | 28.1 | 213.1 | -2.9 | 0.000 | - |

**Table S7** Highly induced and repressed unique transcripts in DS roots

| **Gene ID** | **RS** | **RCK** | **log2FC** | **FDR** | **Putative function** |
| --- | --- | --- | --- | --- | --- |
| Pav_sc0000484.1_g580.1.mk | 1557.0 | 271.9 | 2.5 | 0.013 | NAC transcription factor NAC4 |
| Pav_sc0000588.1_g090.1.mk | 610.9 | 211.6 | 1.5 | 0.007 | putative U-box domain-containing protein 42 |
| Pav_sc0001339.1_g200.1.mk | 170.4 | 64.5 | 1.4 | 0.037 | probable protein phosphatase 2C 48 |
| Pav_sc0001518.1_g660.1.mk | 138.6 | 39.0 | 1.8 | 0.005 | B-box zinc finger protein 20 |
| Pav_sc0000557.1_g660.1.mk | 218.6 | 78.5 | 1.5 | 0.017 | putative leucine-rich repeat receptor-like serine/threonine-protein kinase At2g24130 |
| Pav_sc0007867.1_g020.1.br | 126.7 | 36.9 | 1.8 | 0.008 | cysteine-rich receptor-like protein kinase 10 |
| Pav_sc0002383.1_g160.1.br | 28.6 | 4.4 | 2.7 | 0.015 | L-type lectin-domain containing receptor kinase IX.1-like |
| Pav_sc0001794.1_g600.1.mk | 52.8 | 6.3 | 3.1 | 0.000 | glycine-rich cell wall structural protein-like |
| Pav_co4048503.1_g010.1.mk | 40.8 | 7.8 | 2.4 | 0.014 | inactive TPR repeat-containing thioredoxin TTL3 |
| Pav_sc0001272.1_g140.1.mk | 3650.4 | 1338.9 | 1.4 | 0.008 | probable alpha,alpha-trehalose-phosphate synthase |
| Pav_sc0003562.1_g160.1.mk | 89.1 | 22.9 | 2.0 | 0.005 | fasciclin-like arabinogalactan protein 14 |
| Pav_sc0004043.1_g010.1.mk | 814.7 | 209.5 | 2.0 | 0.015 | chitotriosidase-1-like |
| Pav_sc0003823.1_g150.1.mk | 244.7 | 4.7 | 5.7 | 0.023 | polygalacturonase |
| Pav_sc0000396.1_g1070.1.mk | 625.8 | 169.1 | 1.9 | 0.003 | pathogenesis-related protein PR-4-like |
| Pav_sc0000568.1_g780.1.br | 63.2 | 4.7 | 3.7 | 0.000 | pathogenesis-related protein 1-like |
| Pav_sc0000480.1_g020.1.mk | 363.6 | 86.0 | 2.1 | 0.020 | peroxisomal membrane protein PEX14 |
| Pav_sc0003766.1_g190.1.br | 534.0 | 49.1 | 3.4 | 0.008 | pathogenesis-related protein 1-like |
| Pav_sc0000558.1_g1030.1.mk | 40.6 | 3.0 | 3.8 | 0.005 | xyloglucan endotransglucosylase/hydrolase protein 2-like |
| Pav_sc0000405.1_g520.1.mk | 10369.8 | 4227.8 | 1.3 | 0.026 | xyloglucan endotransglucosylase/hydrolase protein 9 |
| Pav_sc0000624.1_g200.1.mk | 2897.5 | 1224.7 | 1.2 | 0.037 | probable xyloglucan endotransglucosylase/hydrolase protein 23 |
| Pav_sc0000729.1_g370.1.br | 14.6 | 1.1 | 3.8 | 0.034 | putative germin-like protein 2-1 |
| Pav_sc0000729.1_g380.1.br | 172.1 | 18.4 | 3.2 | 0.000 | putative germin-like protein 2-1 |
| Pav_sc0000729.1_g390.1.br | 223.7 | 31.2 | 2.8 | 0.000 | putative germin-like protein 2-1 |
| Pav_sc0000729.1_g400.1.br | 35.5 | 1.7 | 4.4 | 0.000 | putative germin-like protein 2-1 |
| Pav_sc0005529.1_g010.1.br | 271.0 | 89.7 | 1.6 | 0.010 | putative wall-associated receptor kinase-like 16 |
| Pav_sc0000890.1_g1120.1.mk | 1987.9 | 783.1 | 1.3 | 0.036 | cytochrome P450 CYP82D47-like |
| Pav_sc0001405.1_g1110.1.mk | 89.3 | 10.9 | 3.0 | 0.000 | cytochrome P450 714C2-like |
| Pav_sc0000890.1_g1120.1.mk | 1987.9 | 783.1 | 1.3 | 0.036 | cytochrome P450 CYP82D47-like |
| Pav_sc0001405.1_g1110.1.mk | 89.3 | 10.9 | 3.0 | 0.000 | cytochrome P450 714C2-like |
| Pav_sc0002430.1_g020.1.mk | 483.6 | 190.9 | 1.3 | 0.042 | glutamate receptor 2.7-like |
| Pav_sc0000830.1_g450.1.mk | 38639.3 | 15867.9 | 1.3 | 0.031 | BURP domain-containing protein 3-like |
| Pav_sc0001293.1_g320.1.mk | 1923.2 | 468.7 | 2.0 | 0.000 | kininogen-1-like |
| Pav_sc0001673.1_g250.1.mk | 754.1 | 266.1 | 1.5 | 0.010 | SPX domain-containing membrane protein At4g22990-like |
| Pav_sc0002327.1_g970.1.mk | 54.0 | 2.3 | 4.6 | 0.034 | 2-alkenal reductase (NADP(+)-dependent)-like |
| Pav_sc0000195.1_g1120.1.mk | 2117.3 | 374.6 | 2.5 | 0.016 | WAT1-related protein At1g21890-like |
| Pav_sc0000244.1_g080.1.mk | 162.2 | 24.3 | 2.7 | 0.006 | endochitinase 2-like |
| Pav_sc0000348.1_g220.1.mk | 38.5 | 3.9 | 3.3 | 0.000 | R8H-1 protein |
| Pav_sc0000354.1_g620.1.mk | 3735.4 | 1080.6 | 1.8 | 0.012 | probable nucleoredoxin 1 |
| Pav_sc0004971.1_g080.1.mk | 1195.1 | 188.0 | 2.7 | 0.000 | protein NRT1/ PTR FAMILY 7.3 |
| Pav_sc0006098.1_g090.1.br | 3971.8 | 664.0 | 2.6 | 0.000 | FHA domain-containing protein At4g14490-like |
| **Gene ID** | **RS** | **RCK** | **log2FC** | **FDR** | **Putative function** |
| Pav_sc0000159.1_g200.1.br | 402.2 | 84.7 | 2.2 | 0.015 | hypothetical protein PRUPE_ppa015161mg |
| Pav_sc0000583.1_g120.1.mk | 79.9 | 21.2 | 1.9 | 0.010 | hypothetical protein PRUPE_ppa017815mg |
| Pav_sc0000800.1_g600.1.mk | 830.2 | 345.5 | 1.3 | 0.042 | hypothetical protein PRUPE_ppa014721mg |
| Pav_sc0001009.1_g420.1.mk | 627.6 | 148.0 | 2.1 | 0.050 | hypothetical protein PRUPE_ppa021902mg, partial |
| Pav_sc0001770.1_g120.1.mk | 44.2 | 11.0 | 2.0 | 0.037 | hypothetical protein POPTR_0015s04050g |
| Pav_sc0002106.1_g110.1.br | 209.8 | 70.2 | 1.6 | 0.008 | uncharacterized protein LOC103323136 |
| Pav_sc0002308.1_g040.1.br | 1929.9 | 406.1 | 2.2 | 0.027 | hypothetical protein PRUPE_ppa015887mg |
| Pav_sc0002308.1_g130.1.br | 67.5 | 14.1 | 2.3 | 0.017 | hypothetical protein PRUPE_ppa015887mg |
| Pav_sc0003903.1_g050.1.br | 129.4 | 37.9 | 1.8 | 0.025 | uncharacterized protein LOC103948804 |
| Pav_sc0001181.1_g960.1.mk | 492.7 | 193.2 | 1.4 | 0.035 | uncharacterized protein At1g66480-like |
| Pav_sc0003182.1_g200.1.mk | 528.4 | 178.6 | 1.6 | 0.005 | hypothetical protein PRUPE_ppa026145mg, partial |
| Pav_sc0003705.1_g010.1.mk | 120.1 | 27.8 | 2.1 | 0.043 | hypothetical protein PRUPE_ppa005094mg |
| Pav_sc0004001.1_g050.1.br | 77.2 | 16.4 | 2.2 | 0.001 | hypothetical protein PRUPE_ppa012458mg |
| Pav_sc0012348.1_g010.1.br | 154.7 | 51.6 | 1.6 | 0.012 | uncharacterized protein LOC103323136 |
| Pav_sc0006720.1_g020.1.br | 67.5 | 8.0 | 3.1 | 0.000 | hypothetical protein PRUPE_ppa012458mg |
| Pav_sc0005632.1_g010.1.mk | 38.7 | 5.4 | 2.8 | 0.002 | hypothetical protein PRUPE_ppa015959mg, partial |
| Pav_co4025945.1_g010.1.mk | 808.7 | 14.4 | 5.8 | 0.000 | hypothetical protein PRUPE_ppa005895mg |
| Novel00710 | 136.4 | 45.8 | 1.6 | 0.016 | polyprotein |
| Pav_sc0000129.1_g1230.1.mk | 2331.4 | 229.4 | 3.3 | 0.022 | protein LURP-one-related 6-like |
| Pav_sc0000129.1_g1980.1.mk | 91.0 | 24.7 | 1.9 | 0.014 | alpha-aminoadipic semialdehyde synthase |
| Pav_sc0000138.1_g870.1.mk | 473.3 | 158.1 | 1.6 | 0.005 | homeobox leucine zipper protein |
| Novel01208 | 2332.4 | 836.6 | 1.5 | 0.007 | - |
| Pav_sc0001181.1_g760.1.mk | 190.0 | 61.9 | 1.6 | 0.010 | - |
| Pav_sc0000037.1_g290.1.mk | 42.5 | 5.6 | 2.9 | 0.001 | - |
| Pav_sc0000084.1_g150.1.mk | 63.2 | 14.2 | 2.2 | 0.005 | - |
| Pav_sc0000084.1_g160.1.mk | 306.0 | 71.0 | 2.1 | 0.000 | - |
| Pav_sc0000119.1_g210.1.br | 33284.0 | 9497.9 | 1.8 | 0.000 | - |
| Pav_sc0000119.1_g240.1.br | 68436.8 | 14695.8 | 2.2 | 0.002 | - |
| Pav_sc0011764.1_g010.1.mk | 160.9 | 38.7 | 2.1 | 0.035 | - |
| Pav_sc0000207.1_g1300.1.mk | 0.3 | 42.2 | -6.9 | 0.000 | 9-cis-epoxycarotenoid dioxygenase NCED6, chloroplastic |
| Pav_sc0000354.1_g320.1.mk | 127.5 | 648.3 | -2.3 | 0.000 | U-box domain-containing protein 19-like |
| Pav_sc0000467.1_g1030.1.mk | 42.5 | 156.0 | -1.9 | 0.024 | transcription factor FER-LIKE IRON DEFICIENCY-INDUCED TRANSCRIPTION FACTOR |
| Pav_sc0000648.1_g190.1.mk | 20.0 | 75.7 | -1.9 | 0.012 | transcription factor IBH1-like |
| Pav_sc0000755.1_g370.1.mk | 19.1 | 76.0 | -2.0 | 0.005 | ethylene-responsive transcription factor ERF086 |
| Pav_sc0000907.1_g880.1.mk | 51.9 | 302.6 | -2.5 | 0.000 | F-box protein PP2-B15-like |
| Pav_sc0000988.1_g060.1.mk | 71.8 | 360.8 | -2.3 | 0.042 | transcription factor ORG2-like |
| Pav_sc0001488.1_g330.1.mk | 12.5 | 65.3 | -2.4 | 0.001 | ethylene-responsive transcription factor ERF053-like |
| Pav_sc0002332.1_g010.1.mk | 6.3 | 35.7 | -2.5 | 0.008 | zinc finger homeobox protein 3-like |
| Pav_sc0002839.1_g300.1.mk | 32.9 | 519.2 | -4.0 | 0.005 | ethylene-responsive transcription factor RAP2-11-like |
| Pav_sc0004290.1_g150.1.mk | 168.2 | 427.8 | -1.3 | 0.037 | probable protein phosphatase 2C 72 |
| Pav_sc0000555.1_g050.1.mk | 77.7 | 723.8 | -3.2 | 0.003 | proline-rich protein 4 |
| Pav_sc0000023.1_g230.1.mk | 72.7 | 210.9 | -1.5 | 0.010 | serine carboxypeptidase-like 25 |
| **Gene ID** | **RS** | **RCK** | **log2FC** | **FDR** | **Putative function** |
| Pav_sc0000107.1_g280.1.mk | 70.0 | 225.8 | -1.7 | 0.028 | L-type lectin-domain containing receptor kinase VIII.2-like |
| Pav_sc0001003.1_g260.1.mk | 41.3 | 155.5 | -1.9 | 0.001 | LRR receptor-like serine/threonine-protein kinase GSO1 |
| Pav_sc0004531.1_g060.1.br | 109.4 | 768.2 | -2.8 | 0.010 | 14 kDa proline-rich protein DC2.15-like |
| Pav_sc0000202.1_g070.1.mk | 7.7 | 93.4 | -3.6 | 0.010 | tryptophan aminotransferase-related protein 4-like |
| Pav_sc0000051.1_g400.1.mk | 56.1 | 262.2 | -2.2 | 0.002 | tryptophan synthase beta chain |
| Pav_sc0001084.1_g170.1.mk | 2.6 | 27.9 | -3.4 | 0.045 | non-specific lipid-transfer protein-like |
| Pav_sc0000568.1_g700.1.br | 11.8 | 167.8 | -3.8 | 0.000 | basic form of pathogenesis-related protein 1-like |
| Pav_sc0000107.1_g480.1.br | 0.9 | 130.3 | -7.2 | 0.013 | dehydration responsive element-binding protein |
| Pav_sc0008045.1_g040.1.mk | 18.5 | 72.4 | -2.0 | 0.008 | geraniol 8-hydroxylase-like |
| Pav_sc0000122.1_g070.1.mk | 18.0 | 124.3 | -2.8 | 0.027 | probable receptor-like protein kinase At1g67000 isoform X1 |
| Pav_co4024757.1_g010.1.mk | 23.0 | 123.6 | -2.4 | 0.010 | putative E3 ubiquitin-protein ligase LIN-1 |
| Pav_co4002741.1_g010.1.br | 13.5 | 91.2 | -2.8 | 0.006 | putative wall-associated receptor kinase-like 16 |
| Pav_sc0000351.1_g520.1.br | 15.8 | 116.2 | -2.9 | 0.005 | deacetylvindoline O-acetyltransferase-like |
| Pav_sc0000716.1_g390.1.mk | 53.9 | 367.2 | -2.8 | 0.000 | probable 2-oxoglutarate-dependent dioxygenase AOP1 |
| Pav_sc0000716.1_g410.1.mk | 1146.1 | 4327.7 | -1.9 | 0.040 | probable 2-oxoglutarate-dependent dioxygenase AOP1 |
| Pav_sc0000716.1_g420.1.mk | 661.7 | 3315.3 | -2.3 | 0.000 | 2-oxoglutarate-dependent dioxygenase AOP2-like |
| Pav_sc0000890.1_g630.1.mk | 101.7 | 561.3 | -2.5 | 0.000 | omega-hydroxypalmitate O-feruloyl transferase |
| Pav_sc0000893.1_g490.1.mk | 23.8 | 928.4 | -5.3 | 0.000 | probable S-adenosylmethionine-dependent methyltransferase At5g38100 |
| Pav_sc0000383.1_g560.1.br | 5.5 | 36.0 | -2.7 | 0.010 | cytochrome P450 CYP736A12-like |
| Pav_sc0000395.1_g330.1.mk | 113.6 | 939.0 | -3.0 | 0.037 | cytochrome P450 76A1 |
| Pav_sc0001134.1_g190.1.mk | 76.5 | 224.4 | -1.6 | 0.010 | cytochrome P450 71A25-like |
| Pav_sc0002974.1_g200.1.mk | 41.8 | 119.5 | -1.5 | 0.026 | cytochrome P450 71D9-like |
| Pav_sc0000595.1_g1160.1.br | 1.7 | 20.3 | -3.6 | 0.007 | probable receptor-like protein kinase At5g39020 |
| Pav_sc0000852.1_g830.1.mk | 39.9 | 138.2 | -1.8 | 0.012 | glutamate receptor 2.7-like |
| Pav_sc0000907.1_g760.1.mk | 78.1 | 335.6 | -2.1 | 0.002 | wall-associated receptor kinase-like 20 |
| Pav_sc0000981.1_g370.1.br | 32.4 | 205.6 | -2.7 | 0.000 | putative wall-associated receptor kinase-like 16 |
| Pav_sc0002264.1_g030.1.br | 0.3 | 21.7 | -6.2 | 0.000 | receptor-like protein 2 |
| Pav_sc0004616.1_g080.1.br | 11.0 | 42.1 | -1.9 | 0.037 | receptor-like protein 12 |
| Pav_sc0010351.1_g010.1.mk | 29.2 | 211.7 | -2.9 | 0.000 | wall-associated receptor kinase 1-like |
| Pav_co4001095.1_g010.1.br | 20.0 | 71.9 | -1.8 | 0.012 | ABC transporter B family member 4-like |
| Pav_sc0000396.1_g040.1.mk | 755.4 | 3341.3 | -2.1 | 0.017 | ammonium transporter |
| Pav_sc0000480.1_g640.1.mk | 146.4 | 1328.1 | -3.2 | 0.000 | high-affinity nitrate transporter 2.1-like |
| Pav_sc0000497.1_g190.1.mk | 303.9 | 1264.0 | -2.1 | 0.000 | zinc transporter 5 |
| Pav_sc0000546.1_g020.1.mk | 4.1 | 23.0 | -2.5 | 0.047 | ammonium transporter 3 member 1-like |
| Pav_sc0000652.1_g280.1.mk | 13.5 | 109.0 | -3.0 | 0.000 | aluminum-activated malate transporter 10-like |
| Pav_sc0001125.1_g050.1.mk | 243.5 | 1097.9 | -2.2 | 0.000 | nitrate transporter |
| Pav_sc0002327.1_g380.1.mk | 12.5 | 105.6 | -3.1 | 0.031 | oligopeptide transporter 2-like |
| Pav_sc0003286.1_g010.1.mk | 73.9 | 253.0 | -1.8 | 0.002 | sulfate transporter 2.1-like |
| Pav_sc0007535.1_g010.1.mk | 42.9 | 138.7 | -1.7 | 0.011 | sulfate transporter 2.1-like |
| Pav_co4082063.1_g010.1.br | 99.2 | 282.8 | -1.5 | 0.013 | heat shock cognate 70 kDa protein-like |
| Pav_sc0000009.1_g390.1.mk | 11.7 | 92.2 | -3.0 | 0.000 | carbonic anhydrase 2, chloroplastic-like isoform X1 |
| Pav_sc0000026.1_g140.1.mk | 1.3 | 33.8 | -4.7 | 0.013 | putative expansin-B2 |
| **Gene ID** | **RS** | **RCK** | **log2FC** | **FDR** | **Putative function** |
| Pav_sc0000037.1_g170.1.mk | 604.2 | 2053.3 | -1.8 | 0.001 | protein trichome birefringence-like 37 |
| Pav_sc0000044.1_g360.1.mk | 192.2 | 523.1 | -1.4 | 0.035 | MLO-like protein 4 isoform X1 |
| Pav_sc0000093.1_g140.1.mk | 5.5 | 37.8 | -2.8 | 0.002 | BURP domain-containing protein 5-like |
| Pav_sc0000103.1_g1970.1.mk | 48.3 | 188.1 | -2.0 | 0.011 | abietadienol/abietadienal oxidase |
| Pav_sc0000130.1_g770.1.mk | 5.0 | 27.8 | -2.5 | 0.029 | dirigent protein 6 |
| Pav_sc0000138.1_g1240.1.br | 40.5 | 190.2 | -2.2 | 0.013 | cyclic nucleotide-gated ion channel 1-like |
| Pav_sc0000138.1_g500.1.mk | 6.0 | 86.7 | -3.9 | 0.010 | peroxidase 5-like |
| Pav_sc0000159.1_g100.1.mk | 3.4 | 39.5 | -3.5 | 0.000 | casparian strip membrane protein VIT_06s0080g00840 |
| Pav_sc0000174.1_g270.1.mk | 78.4 | 419.6 | -2.4 | 0.050 | putative E3 ubiquitin-protein ligase LIN-1 |
| Pav_sc0000195.1_g040.1.mk | 179.0 | 867.9 | -2.3 | 0.000 | 1-aminocyclopropane-1-carboxylate oxidase |
| Pav_sc0000207.1_g980.1.mk | 70.0 | 183.4 | -1.4 | 0.036 | dirigent protein 16-like |
| Pav_sc0000212.1_g1480.1.mk | 4.2 | 41.6 | -3.3 | 0.005 | 1-aminocyclopropane-1-carboxylate synthase 7 |
| Pav_sc0000231.1_g1380.1.br | 13.2 | 62.7 | -2.2 | 0.004 | predicted protein |
| Pav_sc0000243.1_g290.1.mk | 28.8 | 110.7 | -1.9 | 0.042 | shikimate O-hydroxycinnamoyltransferase-like |
| Pav_sc0000257.1_g1060.1.mk | 26.6 | 85.3 | -1.7 | 0.017 | probable pectinesterase/pectinesterase inhibitor 20 |
| Pav_sc0000259.1_g590.1.mk | 137.4 | 539.7 | -2.0 | 0.000 | germin-like protein subfamily T member 2 |
| Pav_sc0000290.1_g230.1.mk | 3.3 | 49.3 | -3.9 | 0.005 | peroxidase 24-like |
| Pav_sc0000348.1_g980.1.mk | 13.3 | 56.5 | -2.1 | 0.010 | protein LYK5-like |
| Pav_sc0000362.1_g080.1.mk | 164.2 | 769.7 | -2.2 | 0.000 | aquaporin PIP 2.2 |
| Pav_sc0000373.1_g840.1.mk | 1.7 | 16.6 | -3.3 | 0.030 | pentatricopeptide repeat-containing protein At4g35130, chloroplastic-like |
| Pav_sc0000375.1_g250.1.mk | 12.0 | 46.8 | -2.0 | 0.027 | putative protein TPRXL |
| Pav_sc0000383.1_g610.1.mk | 8.2 | 60.0 | -2.9 | 0.000 | cell number regulator 2-like |
| Pav_sc0000554.1_g1670.1.mk | 2.1 | 20.0 | -3.3 | 0.012 | aquaporin TIP1-3 |
| Pav_sc0000554.1_g2050.1.mk | 76.0 | 371.5 | -2.3 | 0.001 | dirigent protein 24 |
| Pav_sc0000588.1_g520.1.mk | 29.0 | 87.4 | -1.6 | 0.034 | strictosidine synthase 3-like |
| Pav_sc0000589.1_g060.1.mk | 835.2 | 1947.2 | -1.2 | 0.044 | probable aquaporin NIP5-1 |
| Pav_sc0000648.1_g150.1.mk | 77.6 | 1802.1 | -4.5 | 0.001 | putative lipid-transfer protein DIR1 |
| Pav_sc0000659.1_g330.1.mk | 1.3 | 22.7 | -4.1 | 0.001 | subtilisin-like protease |
| Pav_sc0000789.1_g430.1.mk | 164.8 | 565.0 | -1.8 | 0.000 | cyclic nucleotide-gated ion channel 1-like isoform X1 |
| Pav_sc0000848.1_g180.1.mk | 298.3 | 1643.7 | -2.5 | 0.007 | peroxidase 3-like |
| Pav_sc0000886.1_g550.1.mk | 549.6 | 2401.6 | -2.1 | 0.002 | probable polygalacturonase |
| Pav_sc0000890.1_g720.1.mk | 123.0 | 339.9 | -1.5 | 0.024 | protein PPLZ02 |
| Pav_sc0000910.1_g880.1.mk | 469.1 | 1800.9 | -1.9 | 0.004 | peroxidase 27 |
| Pav_sc0000981.1_g320.1.br | 7.6 | 36.9 | -2.3 | 0.017 | mitogen-activated protein kinase kinase kinase ANP1-like |
| Pav_sc0001046.1_g060.1.mk | 200.5 | 488.2 | -1.3 | 0.044 | RNA-binding protein 24-B |
| Pav_sc0001080.1_g360.1.mk | 143.7 | 399.9 | -1.5 | 0.011 | zerumbone synthase-like isoform X2 |
| Pav_sc0001084.1_g180.1.mk | 250.4 | 604.2 | -1.3 | 0.038 | EH domain-containing protein 1-like |
| Pav_sc0001189.1_g020.1.mk | 70.3 | 314.0 | -2.2 | 0.000 | putative phytosulfokines 6 |
| Pav_sc0001258.1_g220.1.mk | 34.4 | 100.4 | -1.5 | 0.028 | cyclin-A2-1 |
| Pav_sc0001258.1_g480.1.mk | 28.7 | 125.5 | -2.1 | 0.000 | conserved hypothetical protein |
| Pav_sc0001429.1_g110.1.mk | 941.2 | 3901.7 | -2.1 | 0.024 | 2-oxoglutarate-dependent dioxygenase AOP2-like |
| Pav_sc0001497.1_g010.1.mk | 63.7 | 356.6 | -2.5 | 0.034 | putative lipid-transfer protein DIR1 |
| Pav_sc0001503.1_g240.1.mk | 136.6 | 477.3 | -1.8 | 0.027 | protein trichome birefringence-like 3 |
| **Gene ID** | **RS** | **RCK** | **log2FC** | **FDR** | **Putative function** |
| Pav_sc0001583.1_g420.1.mk | 49.7 | 143.4 | -1.5 | 0.021 | LOB domain-containing protein 4 |
| Pav_sc0001685.1_g020.1.mk | 37.4 | 238.0 | -2.7 | 0.001 | putative lipid-transfer protein DIR1 |
| Pav_sc0001702.1_g030.1.mk | 203.4 | 797.3 | -2.0 | 0.000 | heavy metal-associated isoprenylated plant protein 26-like |
| Pav_sc0001794.1_g270.1.mk | 134.5 | 365.7 | -1.4 | 0.031 | probable glutathione S-transferase |
| Pav_sc0001856.1_g010.1.mk | 99.7 | 273.4 | -1.5 | 0.036 | bifunctional epoxide hydrolase 2-like |
| Pav_sc0001888.1_g110.1.mk | 6.9 | 49.3 | -2.8 | 0.010 | B3 domain-containing protein Os03g0619600-like |
| Pav_sc0001983.1_g060.1.mk | 17.2 | 59.5 | -1.8 | 0.031 | protein YLS2-like |
| Pav_sc0002052.1_g040.1.mk | 2.0 | 25.9 | -3.7 | 0.001 | TMV resistance protein N-like |
| Pav_sc0002154.1_g160.1.mk | 56.5 | 379.5 | -2.7 | 0.000 | IQ motif and SEC7 domain-containing protein 3 |
| Pav_sc0002697.1_g080.1.br | 183.9 | 507.6 | -1.5 | 0.014 | probable mannitol dehydrogenase |
| Pav_sc0002869.1_g250.1.mk | 12.9 | 78.9 | -2.6 | 0.000 | protein YLS9-like |
| Pav_sc0002914.1_g080.1.mk | 1.7 | 15.9 | -3.2 | 0.044 | laccase-11-like, partial |
| Pav_sc0002914.1_g090.1.mk | 2.1 | 21.4 | -3.4 | 0.007 | laccase-11-like |
| Pav_sc0003079.1_g050.1.mk | 24.1 | 174.7 | -2.9 | 0.000 | probable mannitol dehydrogenase |
| Pav_sc0003084.1_g020.1.br | 1.4 | 28.0 | -4.3 | 0.000 | stigma-specific STIG1-like protein 1 |
| Pav_sc0003258.1_g050.1.br | 16.4 | 57.9 | -1.8 | 0.026 | laccase-11-like |
| Pav_sc0003685.1_g040.1.mk | 8.7 | 60.4 | -2.8 | 0.000 | conserved hypothetical protein |
| Pav_sc0003696.1_g090.1.mk | 10.9 | 43.0 | -2.0 | 0.042 | bifunctional monodehydroascorbate reductase and carbonic anhydrase nectarin-3 |
| Pav_sc0003802.1_g020.1.br | 3.7 | 29.4 | -3.0 | 0.004 | UPF0481 protein At3g47200-like |
| Pav_sc0003868.1_g040.1.mk | 32.1 | 144.0 | -2.2 | 0.004 | dirigent protein 25-like |
| Pav_sc0012539.1_g030.1.br | 0.3 | 24.3 | -6.4 | 0.000 | palmitoyl-monogalactosyldiacylglycerol delta-7 desaturase, chloroplastic - like |
| Novel01595 | 14.4 | 62.1 | -2.1 | 0.006 | hypothetical protein PRUPE_ppa021021mg |
| Pav_sc0000024.1_g430.1.mk | 1.3 | 30.2 | -4.5 | 0.000 | hypothetical protein B456_013G004000 |
| Pav_sc0000449.1_g440.1.mk | 1097.6 | 2552.7 | -1.2 | 0.044 | hypothetical protein PRUPE_ppa009460mg |
| Pav_sc0000491.1_g830.1.mk | 173.0 | 496.1 | -1.5 | 0.008 | hypothetical protein PRUPE_ppa004818mg |
| Pav_sc0000506.1_g370.1.mk | 16.2 | 58.4 | -1.9 | 0.017 | hypothetical protein PRUPE_ppa021966mg |
| Novel01251 | 79.2 | 216.3 | -1.4 | 0.017 | hypothetical protein PRUPE_ppa020321mg |
| Pav_sc0000677.1_g300.1.mk | 18.3 | 131.1 | -2.8 | 0.000 | hypothetical protein PRUPE_ppa021336mg, partial |
| Pav_sc0000598.1_g260.1.mk | 712.3 | 1780.7 | -1.3 | 0.019 | hypothetical protein PRUPE_ppa005876mg |
| Pav_sc0000598.1_g390.1.mk | 201.5 | 781.1 | -2.0 | 0.002 | hypothetical protein PRUPE_ppa007964mg |
| Pav_sc0002055.1_g090.1.mk | 30.9 | 107.2 | -1.8 | 0.006 | hypothetical protein PRUPE_ppa018544mg |
| Pav_sc0002326.1_g200.1.mk | 11.7 | 52.8 | -2.2 | 0.006 | hypothetical protein CICLE_v10013026mg |
| Pav_sc0002207.1_g380.1.mk | 30.4 | 96.0 | -1.7 | 0.016 | hypothetical protein PRUPE_ppa024220mg |
| Pav_sc0000912.1_g040.1.mk | 12.0 | 59.9 | -2.3 | 0.006 | hypothetical protein B456_002G136200 |
| Pav_sc0001009.1_g290.1.mk | 81.5 | 251.8 | -1.6 | 0.006 | hypothetical protein PRUPE_ppa024827mg |
| Pav_sc0001428.1_g420.1.mk | 46.8 | 287.7 | -2.6 | 0.000 | hypothetical protein PRUPE_ppa025934mg |
| Pav_sc0001488.1_g090.1.mk | 70.9 | 186.8 | -1.4 | 0.031 | hypothetical protein PRUPE_ppa014753mg |
| Pav_sc0003766.1_g050.1.mk | 292.0 | 887.6 | -1.6 | 0.006 | hypothetical protein L484_003734 |
| Pav_sc0003619.1_g010.1.br | 32.4 | 129.7 | -2.0 | 0.002 | hypothetical protein PRUPE_ppa008070mg |
| Pav_sc0000254.1_g1300.1.mk | 313.5 | 870.9 | -1.5 | 0.042 | hypothetical protein CISIN_1g0090752mg |
| Pav_sc0001932.1_g180.1.mk | 15.9 | 148.9 | -3.2 | 0.000 | uncharacterized protein LOC103334218 |
| **Gene ID** | **RS** | **RCK** | **log2FC** | **FDR** | **Putative function** |
| Pav_sc0001073.1_g200.1.mk | 133.9 | 437.4 | -1.7 | 0.045 | uncharacterized protein LOC103329395 isoform X2 |
| Novel01561 | 86.0 | 237.6 | -1.5 | 0.028 | uncharacterized protein LOC103336576 isoform X2 |
| Pav_sc0001175.1_g290.1.mk | 0.3 | 14.2 | -5.6 | 0.019 | uncharacterized protein LOC103340052 |
| Pav_sc0000910.1_g460.1.mk | 18.6 | 163.9 | -3.1 | 0.031 | uncharacterized protein LOC103324526 |
| Novel00190 | 112.2 | 282.7 | -1.3 | 0.034 | uncharacterized protein LOC103340122 |
| Pav_sc0005678.1_g070.1.mk | 39.1 | 120.3 | -1.6 | 0.012 | uncharacterized protein LOC103323917 |
| Pav_sc0006018.1_g100.1.mk | 3.6 | 32.0 | -3.1 | 0.002 | Uncharacterized protein TCM_014487 |
| Pav_sc0006298.1_g050.1.mk | 29.1 | 106.4 | -1.9 | 0.009 | uncharacterized protein At1g04910-like |
| Pav_sc0000465.1_g440.1.br | 12.0 | 53.0 | -2.1 | 0.008 | - |
| Novel01523 | 30.4 | 93.6 | -1.6 | 0.028 | - |
| Novel00681 | 25.1 | 89.6 | -1.8 | 0.008 | - |
| Pav_sc0000729.1_g180.1.br | 659.6 | 1518.9 | -1.2 | 0.046 | - |
| Pav_sc0000793.1_g110.1.br | 6.7 | 35.7 | -2.4 | 0.010 | - |
| Pav_sc0001797.1_g450.1.mk | 0.7 | 30.9 | -5.5 | 0.000 | - |
| Pav_sc0011059.1_g010.1.mk | 2.3 | 174.5 | -6.2 | 0.000 | - |

**Table S8** Explanation and predictability values of the principal component analysis (PCA) and partial least squares-discriminate analysis (PLS-DA)

| **Type** | **A** | **N** | **R^2^X(cum)** | **R^2^Y(cum)** | **Q^2^ (cum)** | **Title** |
| --- | --- | --- | --- | --- | --- | --- |
| PCA-X | 6 | 30 | 0.505 | - |  | TOTAL with QC |
| PCA-X | 5 | 24 | 0.503 | - |  | TOTAL |
| PCA-X | 4 | 12 | 0.569 | - |  | CCL-CSL |
| PCA-X | 4 | 12 | 0.592 | - |  | GCL-GSL |
| OPLS-DA | 1+1+0 | 12 | 0.304 | 0.997 | 0.767 | CCL-CSL |
| OPLS-DA | 1+1+0 | 12 | 0.317 | 0.961 | 0.509 | GCL-GSL |

**Table S9** Differential drought responded up regulated metabolites in DT

| **Metabolites** | **Mean CCL** | **Mean CSL** | **FC** | **VIP** | **P-value** |
| --- | --- | --- | --- | --- | --- |
| Melibiose | 0.4000 | 0.6473 | 1.6 | 1.045 | 0.023 |
| Flavin adenine degrad product | 0.4215 | 0.5502 | 1.3 | 1.034 | 0.015 |
| Salicin | 0.1064 | 0.1304 | 1.2 | 1.026 | 0.023 |
| Serine | 0.0813 | 0.0944 | 1.2 | 1.064 | 0.007 |
| 4-aminobutyric acid | 0.0717 | 0.0856 | 1.2 | 1.033 | 0.037 |
| D-erythro-sphingosine | 0.0522 | 0.0776 | 1.5 | 1.414 | 0.011 |
| Farnesal | 0.0468 | 0.0736 | 1.6 | 1.191 | 0.041 |
| Maltitol | 0.0470 | 0.0586 | 1.2 | 1.072 | 0.042 |
| Citrulline | 0.0205 | 0.0469 | 2.3 | 1.353 | 0.010 |
| Glycine | 0.0212 | 0.0440 | 2.1 | 1.062 | 0.030 |
| Citric acid | 0.0270 | 0.0419 | 1.6 | 1.056 | 0.046 |
| Itaconic acid | 0.0283 | 0.0397 | 1.4 | 1.298 | 0.016 |
| Ornithine | 0.0259 | 0.0348 | 1.3 | 1.025 | 0.036 |
| Valine | 0.0096 | 0.0314 | 3.3 | 1.316 | 0.046 |
| Ribose | 0.0160 | 0.0311 | 1.9 | 1.449 | 0.033 |
| Arbutin | 0.0067 | 0.0216 | 3.2 | 1.009 | 0.000 |
| Dihydrocoumarin | 0.0130 | 0.0178 | 1.4 | 1.067 | 0.038 |
| Lactic acid | 0.0153 | 0.0172 | 1.1 | 1.001 | 0.038 |
| 2-hydroxy-3-isopropylbutanedioic acid | 0.0100 | 0.0132 | 1.3 | 1.541 | 0.041 |
| Glucoheptonic acid | 0.0056 | 0.0120 | 2.1 | 1.807 | 0.018 |
| Lactitol | 0.0064 | 0.0105 | 1.6 | 1.187 | 0.019 |
| P-Coumaric acid | 0.0063 | 0.0275 | 4.4 | 1.032 | 0.032 |
| Galactonic acid | 0.0057 | 0.0103 | 1.8 | 1.076 | 0.021 |
| Cycloleucine | 0.0057 | 0.0099 | 1.7 | 1.058 | 0.029 |
| Lyxose | 0.0067 | 0.0095 | 1.4 | 1.346 | 0.013 |
| Maleimide | 0.0063 | 0.0092 | 1.5 | 1.515 | 0.029 |
| 2-Monopalmitin | 0.0057 | 0.0088 | 1.5 | 1.081 | 0.013 |
| Isoleucine | 0.0057 | 0.0085 | 1.5 | 1.520 | 0.032 |
| 2-Deoxytetronic acid | 0.0053 | 0.0085 | 1.6 | 1.179 | 0.028 |
| O-cresol | 0.0042 | 0.0076 | 1.8 | 2.177 | 0.026 |
| N-Acetyl-beta-D-mannosamine | 0.0027 | 0.0075 | 2.8 | 1.787 | 0.005 |
| Toluenesulfonic acid | 0.0011 | 0.0062 | 5.4 | 1.482 | 0.036 |
| Threo-beta-hyrdoxyaspartate | 0.0055 | 0.0059 | 1.1 | 1.172 | 0.024 |
| Cellobiose | 0.0038 | 0.0055 | 1.5 | 1.887 | 0.000 |
| Isopropyl-beta-D-thiogalactopyranoside | 0.0009 | 0.0040 | 4.7 | 1.201 | 0.020 |
| Methyl trans-cinnamate | 0.0018 | 0.0038 | 2.1 | 1.273 | 0.018 |
| Benzoic acid | 0.0023 | 0.0037 | 1.6 | 1.067 | 0.003 |
| Cis-gondoic acid | 0.0024 | 0.0033 | 1.4 | 1.769 | 0.038 |
| Tryptophan | 0.0012 | 0.0032 | 2.7 | 1.002 | 0.031 |
| Glutaconic acid | 0.0005 | 0.0032 | 5.9 | 1.351 | 0.006 |
| Linolenic acid | 0.0011 | 0.0029 | 2.6 | 1.285 | 0.041 |
| Melezitose | 0.0024 | 0.0029 | 1.2 | 1.368 | 0.031 |
| Neohesperidin | 0.0016 | 0.0027 | 1.7 | 1.068 | 0.018 |
| Beta-Alanine | 0.0014 | 0.0026 | 1.9 | 2.151 | 0.026 |
| 1,5-Anhydroglucitol | 0.0020 | 0.0025 | 1.3 | 1.859 | 0.001 |
| Maltose | 0.0021 | 0.0024 | 1.1 | 1.413 | 0.030 |
| **Metabolites** | **Mean CCL** | **Mean CSL** | **FC** | **VIP** | **P-value** |
| P-benzoquinone | 0.0000 | 0.0024 | 283784.1 | 1.195 | 0.026 |
| 1,2,4-Benzenetriol | 0.0002 | 0.0011 | 6.9 | 1.045 | 0.011 |
| 1-Aminocyclopropanecarboxylic acid | 0.0011 | 0.0011 | 1.0 | 1.432 | 0.023 |
| 3-Hydroxypropionic acid | 0.0009 | 0.0011 | 1.2 | 1.428 | 0.048 |
| 5-Aminovaleric acid | 0.0002 | 0.0011 | 7.1 | 2.136 | 0.001 |
| Sucrose-6-Phosphate | 0.0001 | 0.0010 | 12.9 | 1.266 | 0.022 |
| o-Hydroxyhippuric acid | 0.0007 | 0.0009 | 1.3 | 1.510 | 0.046 |
| 2,3-Dimethylsuccinic acid | 0.0001 | 0.0008 | 6.7 | 1.030 | 0.047 |
| (+)-catechin | 0.0002 | 0.0008 | 5.0 | 1.133 | 0.019 |
| Cytidine-monophosphate degr prod | 0.0002 | 0.0007 | 4.5 | 1.142 | 0.019 |
| Uridine | 0.0001 | 0.0006 | 6.0 | 1.002 | 0.035 |
| Guanine | 0.0000 | 0.0001 | - | 1.033 | 0.039 |
| 2-Oxoglutarate | 0.0000 | 0.0001 | - | 1.210 | 0.045 |

**CSL** and **CCL** represent drought treatment group and control group of DT leaves, respectively. **FC** (Fold change) indicates the ratio of peak amount of treatment samples (CSL) and control samples (CCL). Data difference significance *p* < 0.05. Since the relative content of substances was detected by non-target GC-MS, there is no unit.

**Table S10** Differential drought responded up regulated metabolites in DS

| **Metabolites** | **Mean GCL** | **Mean GSL** | **FC** | **VIP** | ***P*-value** |
| --- | --- | --- | --- | --- | --- |
| Maleic acid | 0.2536 | 0.3002 | 1.2 | 1.165 | 0.037 |
| Alanine | 0.1218 | 0.2001 | 1.6 | 1.456 | 0.010 |
| D-Glyceric acid | 0.0368 | 0.0502 | 1.4 | 1.076 | 0.033 |
| Malonic acid | 0.0292 | 0.0429 | 1.5 | 1.055 | 0.021 |
| Valine | 0.0190 | 0.0352 | 1.9 | 1.071 | 0.030 |
| Galactinol | 0.0197 | 0.0297 | 1.5 | 1.062 | 0.033 |
| Lactobionic acid | 0.0074 | 0.0163 | 2.2 | 1.085 | 0.024 |
| DL-dihydrosphingosine | 0.0103 | 0.0154 | 1.5 | 1.613 | 0.006 |
| Isoleucine | 0.0040 | 0.0146 | 3.6 | 1.349 | 0.012 |
| Saccharic acid | 0.0089 | 0.0108 | 1.2 | 1.273 | 0.049 |
| Caffeic acid | 0.0086 | 0.0132 | 1.5 | 1.004 | 0.025 |
| 5-aminovaleric acid lactam | 0.0049 | 0.0106 | 2.2 | 1.251 | 0.021 |
| Chlorogenic acid | 0.0032 | 0.0062 | 2.0 | 1.059 | 0.019 |
| D-erythronolactone | 0.0030 | 0.0046 | 1.5 | 1.412 | 0.039 |
| Threo-beta-hyrdoxyaspartate | 0.0020 | 0.0043 | 2.1 | 1.227 | 0.035 |
| Neohesperidin | 0.0018 | 0.0034 | 1.9 | 1.414 | 0.035 |
| Maltotriose | 0.0021 | 0.0028 | 1.3 | 1.423 | 0.045 |
| 4-hydroxy-3-methoxycinnamaldehyde | 0.0021 | 0.0027 | 1.3 | 1.202 | 0.041 |
| Mucic acid | 0.0030 | 0.0038 | 1.4 | 1.020 | 0.049 |

**GSL** and **GCL** represent drought treatment group and control group of DS leaves. FC (Fold change) indicates the ratio of peak amount of treatment samples (GSL) and control samples (GCL). Data difference significance *p* < 0.05. Since the relative content of substances was detected by non-target GC-MS, there is no unit.

**Table S11** Candidate DEGs involved in KEGG pathways that are related to differential metabolites

| **Pathway** | **Metabolites** | **Metabolites ID** | **Gene number** | **NCBI- Gene ID** | **Annotation** | **Fold Change** |
| --- | --- | --- | --- | --- | --- | --- |
| Cyanoamino acid metabolism | 3-Cyanoalanine | C02512 | Pav_co4061311.1_g010.1.br | 18770479 | Amygdalin hydrolase isoform AH I precursor | 2.65 |
|  | L-Asparagine | C00152 | Pav_sc0001485.1_g120.1.mk | 18778261 | Beta-glucosidase 41 isoform X1 | 1.89 |
|  | L-Phenylalanine | C00079 | Pav_sc0005750.1_g010.1.br | 18774021 | Beta-glucosidase 11-like | 2.05 |
|  | *glycine | C00037 | Pav_sc0002706.1_g010.1.mk | 18790921 | (R)-mandelonitrile lyase 1-like | 3.28 |
|  | *Serine | C00065 | Pav_sc0000308.1_g080.1.mk | 18790921 | Bifunctional dethiobiotin synthetase/7,8-diamino-pelargonic acid | 3.65 |
|  | ^#^alanine | C01401 | Pav_sc0001251.1_g440.1.mk | 18774793 | Lysosomal beta glucosidase-like | -1.27 |
|  | ^#^valine | C00183 | Pav_sc0000220.1_g180.1.mk | 18792862 | Beta-glucosidase 40-like | 2.26 |
|  | ^#^Isoleucine | C00407 | Pav_sc0002308.1_g040.1.br | 18775538 | Hypothetical protein PRUPE_ppa015887mg | 2.25 |
|  |  |  | Pav_sc0002308.1_g130.1.br | 18766146 | Hypothetical protein PRUPE_ppa015887mg | 2.26 |
|  |  |  | Pav_sc0000159.1_g200.1.br | 18774013 | Hypothetical protein PRUPE_ppa015161mg | 2.25 |
| Phenylpropanoid biosynthesis | phytosphingosine | C00079 | Pav_sc0000636.1_g260.1.mk | 18786755 | 4-coumarate-CoA ligase | 1.29 |
|  | quinic acid | C00296 | Pav_sc0001485.1_g120.1.mk | 18778261 | Beta-glucosidase 41 isoform X1 | 1.89 |
|  | p-Coumaric acid | C00811 | Pav_sc0005750.1_g010.1.br | 18774021 | Beta-glucosidase 11-like | 2.05 |
|  | *citric acid | C00158 | Pav_co4061311.1_g010.1.br | 18770479 | Amygdalin hydrolase isoform AH I precursor | 2.65 |
|  | ^#^Chlorogenic acid | C00852 | Pav_sc0000869.1_g590.1.mk | 18777009 | Aaffeoylshikimate esterase | -1.4 |
|  | ^#^4-hydroxy-3-methoxycinnamaldehyde | C02666 | Pav_sc0003326.1_g220.1.mk | 18783187 | Shikimate O-hydroxycinnamoyltransferase-like | -1.81 |
|  | ^#^Caffeic acid | C01197 | Novel01421 | 18783187 | Hypothetical protein PRUPE_ppa005910mg | -1.79 |
|  |  |  | Pav_sc0000625.1_g130.1.mk | 18767368 | Hypothetical protein PRUPE_ppa005719mg | -1.38 |
|  |  |  | Pav_sc0001502.1_g260.1.br | 18774869 | Cationic peroxidase 1-like | -3.91 |
|  |  |  | Pav_sc0001502.1_g290.1.mk | 18774869 | Cationic peroxidase 1-like | -3.72 |
|  |  |  | Pav_sc0002544.1_g080.1.mk | 18773443 | Peroxidase 73-like | -2.43 |
| **Pathway** | **Metabolites** | **Metabolites ID** | **Gene number** | **NCBI- Gene ID** | **Annotation** | **Fold Change** |
|  |  |  | Pav_sc0000220.1_g180.1.mk | 18792862 | Beta-glucosidase 40-like | 2.26 |
|  |  |  | Pav_sc0001323.1_g1090.1.mk | 18793937 | Peroxidase 43 | 2.08 |
|  |  |  | Pav_sc0001502.1_g290.1.mk | 18774869 | Cationic peroxidase 1-like | -1.98 |
|  |  |  | Pav_sc0003921.1_g050.1.mk | 18774869 | Probable mannitol dehydrogenase | -1.85 |
|  |  |  | Pav_sc0003079.1_g020.1.mk | 18773431 | Probable mannitol dehydrogenase | -1.81 |
|  |  |  | Pav_sc0001502.1_g370.1.mk | 18774869 | Cationic peroxidase 1-like | -1.97 |
|  |  |  | Pav_sc0003681.1_g080.1.mk | 18782933 | Shikimate O-hydroxycinnamoyltransferase-like | -2.27 |
| Phenylalanine, tyrosine and tryptophan biosynthesis | quinic acid | C00296 | Pav_sc0001189.1_g030.1.mk | 18774516 | Arogenate dehydratase/prephenate dehydratase 6, chloroplastic-like | -1.78 |
|  | phenylalanine | C00079 | Pav_sc0000002.1_g060.1.mk | 18773898 | Bifunctional 3-dehydroquinate dehydratase/shikimate dehydrogenase, chloroplastic-like isoform X1 | -1.98 |
|  |  |  | Pav_sc0006673.1_g020.1.mk | 18774550 | Phospho-2-dehydro-3-deoxyheptonate aldolase 2, chloroplastic-like | -1.83 |
| ABC transporters | Biotin | C00120 | Pav_sc0000766.1_g260.1.mk | 18782071 | ABC transporter B family member 19 | -1.5 |
|  | sulfuric acid | C00059 | Pav_co4069733.1_g010.1.mk | 18782071 | ABC transporter B family member 19 | -1.68 |
|  | proline | C00148 | Pav_sc0000493.1_g120.1.mk | 18788814 | ABC transporter B family member 26, chloroplastic isoform X1 | -2.13 |
|  | *glycine | C00037 | Pav_sc0000216.1_g510.1.mk | 18780866 | 2-oxoisovalerate dehydrogenase subunit alpha 2, mitochondrial | 1.13 |
|  | ^＃^Maltotriose | C01835 |  |  |  |  |
| Ascorbate and aldarate metabolism | *2-Oxoglutarate | C00026 | Pav_sc0006478.1_g020.1.mk | 18772834 | L-ascorbate oxidase-like | -1.08 |
|  | ^#^D-Glyceric acid | C00879 | Pav_sc0000023.1_g530.1.mk | 18783311 | UDP-glucose 6-dehydrogenase 1-like | -1.12 |
|  | ^#^mucic acid | C00879 |  |  |  |  |
| **Pathway** | **Metabolites** | **Metabolites ID** | **Gene number** | **NCBI- Gene ID** | **Annotation** | **Fold Change** |
| Arginine and proline metabolism | *4-aminobutyric acid | C00431 | Pav_co4065321.1_g010.1.mk | 18772536 | Delta-1-pyrroline-5-carboxylate synthase-like isoform X2 | 2.21 |
|  | *citrulline | C00334 | Pav_sc0000554.1_g290.1.mk | 18785402 | Delta-1-pyrroline-5-carboxylate synthase | 1.2 |
|  | ^#^5-aminovaleric acid lactam | C00327 | Pav_sc0000955.1_g090.1.mk | 18772536 | Delta-1-pyrroline-5-carboxylate synthase-like isoform X2 | 2.17 |
|  |  |  | Pav_sc0002148.1_g120.1.mk | 18787435 | Arginase 1, mitochondrial | -1.57 |
|  |  |  | Pav_co4060979.1_g010.1.mk | 18787435 | Arginase 1, mitochondrial | -3.63 |
|  |  |  | Pav_sc0001124.1_g400.1.mk | 18782544 | Probable polyamine oxidase 4 | -1.31 |
|  |  |  | Pav_sc0000069.1_g180.1.mk | 18789648 | Thermospermine synthase ACAULIS5-like | -1.56 |
|  |  |  | Pav_sc0002148.1_g090.1.mk | 18787435 | Arginase 1, mitochondrial | -1.7 |
|  |  |  | Pav_sc0000588.1_g480.1.mk | 18788515 | S-adenosylmethionine decarboxylase proenzyme | 1.52 |
| Purine metabolism | *guanine | C00242 | Pav_sc0000103.1_g1360.1.mk | 18766957 | Glutamate decarboxylase 4 isoform 2 | 1.36 |
|  |  |  | Pav_sc0000020.1_g070.1.mk | 18780660 | Allantoate deiminase | 1.36 |
|  |  |  | Pav_sc0000789.1_g290.1.mk | 18785374 | Adenylate kinase-like | 1.19 |
|  |  |  | Pav_sc0001365.1_g030.1.mk | 18780660 | Allantoate deiminase | 1.66 |
|  |  |  | Pav_sc0002207.1_g610.1.mk | 18769265 | 55 kDa erythrocyte membrane protein | -1.23 |
| Biosynthesis of amino acids | *citrulline | C00327 | Pav_sc0000582.1_g980.1.mk | 18767698 | Bifunctional aspartokinase/homoserine dehydrogenase 1, chloroplastic-like isoform X1 | 1.28 |
|  | *glycine | C00037 | Pav_sc0000414.1_g050.1.mk | 18771390 | Serine acetyltransferase 2 | 1.01 |
|  |  |  | Pav_sc0000955.1_g090.1.mk | 18772536 | Delta-1-pyrroline-5-carboxylate synthase-like isoform X2 | 2.17 |
|  |  |  | Pav_sc0000554.1_g290.1.mk | 18785402 | Delta-1-pyrroline-5-carboxylate synthase | 1.2 |
|  |  |  | Pav_co4065321.1_g010.1.mk | 18772536 | Delta-1-pyrroline-5-carboxylate synthase-like isoform X2 | 2.21 |
| **Pathway** | **Metabolites** | **Metabolites ID** | **Gene number** | **NCBI- Gene ID** | **Annotation** | **Fold Change** |
| Glycine, serine and threonine metabolism | L-Allothreonine | C05519 | Pav_sc0000582.1_g980.1.mk | 18767698 | Bifunctional aspartokinase/homoserine dehydrogenase 1, chloroplastic-like isoform X1 | 1.28 |
|  | *glycine | C00037 | Pav_sc0001080.1_g340.1.mk | 18782864 | Aspartokinase 2, chloroplastic isoform X1 | -1 |
|  | ^＃^D-Glyceric acid | C00258 | Pav_sc0000582.1_g980.1.mk | 18767698 | Bifunctional aspartokinase/homoserine dehydrogenase 1, chloroplastic-like isoform X1 | -1.76 |
| Pyrimidine metabolism | *Uridine | C00299 | Pav_sc0000103.1_g1360.1.mk | 18766957 | Glutamate decarboxylase 4 isoform 2 | 1.36 |
|  | ^＃^malonic acid | C00383 | Pav_sc0000009.1_g080.1.mk | 18782030 | Polyribonucleotide nucleotidyltransferase 2, mitochondrial | -1.78 |
|  |  |  | Pav_sc0000129.1_g180.1.mk | 18771206 | Acid phosphatase | -2.92 |
| Tyrosine metabolism | ^＃^maleic acid | C01384 | Pav_sc0003492.1_g410.1.mk | 18780245 | Homogentisate 1,2-dioxygenase | 1.1 |
| Sphingolipid metabolism | phytosphingosine | C12144 | Pav_sc0001030.1_g160.1.mk | 18788797 | Alpha-galactosidase-like | -1.63 |
| Terpenoid backbone biosynthesis | *Farnesal | C03461 | Pav_sc0000095.1_g1540.1.mk | 18778932 | Probable solanesyl-diphosphate synthase 3 | 1.32 |
| Glycolysis / Gluconeogenesis | *Arbutin | C06186 | Pav_sc0001371.1_g080.1.mk | 18783348 | Putative glucose-6-phosphate 1-epimerase | 1.51 |
|  |  |  | Pav_sc0001125.1_g130.1.mk | 18776406 | L-lactate dehydrogenase B-like | -1.6 |
|  |  |  | Pav_sc0000766.1_g340.1.mk | 18782310 | Aldose 1-epimerase-like | -1.17 |
| Plant hormone signal transduction | salicylic acid | C00805 | Pav_sc0002858.1_g200.1.mk | 18772600 | Probable protein phosphatase 2C 24 | 3.57 |
|  |  |  | Pav_sc0001335.1_g050.1.mk | 18785692 | Probable protein phosphatase 2C 51 | 4.23 |
|  |  |  | Pav_sc0000212.1_g830.1.mk | 18769799 | Protein phosphatase 2C 56-like | 1.82 |
|  |  |  | Pav_sc0003850.1_g010.1.br | 18785692 | Probable protein phosphatase 2C 51 | Inf |
|  |  |  | Pav_sc0000704.1_g940.1.mk | 18769697 | EIN3-binding F-box protein 1-like | 1.75 |
|  |  |  | Pav_sc0002858.1_g200.1.mk | 18772600 | Probable protein phosphatase 2C 24 | 3.57 |
| **Pathway** | **Metabolites** | **Metabolites ID** | **Gene number** | **NCBI- Gene ID** | **Annotation** | **Fold Change** |
|  |  |  | Pav_sc0001335.1_g050.1.mk | 18785692 | Probable protein phosphatase 2C 51 | 4.23 |
|  |  |  | Pav_sc0000212.1_g830.1.mk | 18769799 | Protein phosphatase 2C 56-like | 1.82 |
|  |  |  | Pav_sc0003850.1_g010.1.br | 18785692 | Probable protein phosphatase 2C 51 | Inf |
|  |  |  | pav_sc0002319.1_g030.1.mk | 18771542 | Two-component response regulator ARR8 | 1.87 |
|  |  |  | Pav_sc0001883.1_g190.1.mk | 18787601 | Two-component response regulator ARR9-like | 1.58 |
|  |  |  | Pav_sc0000716.1_g230.1.mk | 18782650 | Putative RING-H2 finger protein ATL21B | 1.69 |
|  |  |  | Pav_sc0003766.1_g190.1.br | 18766413 | Pathogenesis-related protein 1-like | 3.44 |
|  |  |  | Pav_sc0000568.1_g780.1.br | 18767807 | Pathogenesis-related protein 1-like | 3.74 |

Note: “*”indicates drought responded differential metabolites specifically induced in ‘CDR-1’, “^＃^”indicates drought responded differential metabolites specifically induced in ‘Gisela 5’, the others were common drought responded metabolites in both species; gene number in bold indicates drought responded DEGs specifically induced in ‘CDR-1’,others indicates drought responded DEGs specifically induced in ‘Gisela 5’; FC indicates log2 (Fold Change); “Inf” indicates gene was almost undetectable with low abundance under condition, while drought treatment induced its expression.
